# Genome-wide analysis of WRKY transcription factors in wheat (*Triticum aestivum* L.) and differential expression under water deficit condition

**DOI:** 10.7717/peerj.3232

**Published:** 2017-05-04

**Authors:** Pan Ning, Congcong Liu, Jingquan Kang, Jinyin Lv

**Affiliations:** 1 College of Science, Northwest Agriculture and Forestry University, Yangling, Shaanxi, China; 2 College of Animal Science and Technology, Huazhong Agricultural University, Wuhan, Hubei, China; 3 College of Life Science, Northwest Agriculture and Forestry University, Yangling, Shaanxi, China

**Keywords:** Wheat, WRKY, Water deficit, Expression

## Abstract

**Background:**

WRKY proteins, which comprise one of the largest transcription factor (TF) families in the plant kingdom, play crucial roles in plant development and stress responses. Despite several studies on WRKYs in wheat (*Triticum aestivum* L.), functional annotation information about wheat WRKYs is limited.

**Results:**

Here, 171 TaWRKY TFs were identified from the whole wheat genome and compared with proteins from 19 other species representing nine major plant lineages. A phylogenetic analysis, coupled with gene structure analysis and motif determination, divided these TaWRKYs into seven subgroups (Group I, IIa–e, and III). Chromosomal location showed that most *TaWRKY* genes were enriched on four chromosomes, especially on chromosome 3B. In addition, 85 (49.7%) genes were either tandem (5) or segmental duplication (80), which suggested that though tandem duplication has contributed to the expansion of TaWRKY family, segmental duplication probably played a more pivotal role. Analysis of *cis*-acting elements revealed putative functions of WRKYs in wheat during development as well as under numerous biotic and abiotic stresses. Finally, the expression of *TaWRKY* genes in flag leaves, glumes, and lemmas under water-deficit condition were analyzed. Results showed that different *TaWRKY* genes preferentially express in specific tissue during the grain-filling stage.

**Conclusion:**

Our results provide a more extensive insight on *WRKY* gene family in wheat, and also contribute to the screening of more candidate genes for further investigation on function characterization of WRKYs under various stresses.

## Introduction

Plants have developed a wide range of unique strategies to cope with various biotic and abiotic stresses through physical adaption, molecular, and cellular changes (Ahuja et al., 2010; Knight & Knight, 2001). Transcription regulation of gene expression in response to developmental and environment changes, mediated by the DNA-binding transcription factors (TFs), is an important regulatory mechanism in plants (Ahuja et al., 2010; Buscaill & Rivas, 2014). WRKYs, one of the largest families of regulators, play key roles in numerous stress responses and several development processes (Rushton et al., 2010). Since the first report of WRKY TFs identified in sweet potato (Ishiguro & Nakamura, 1994), WRKY proteins have been found throughout the plant lineage and also in a number of diplomonads, social amoebae, fungi incertae seais, and amoeboza in succession (Rinerson et al., 2015).

WRKY TFs are defined by the presence of one or two highly conserved WRKY domains (WDs) of 60 amino acid residues, including the almost invariant WRKYGQK heptapeptide at the N-terminus, followed by a C_2_H_2_ (C–X_4–5_–C–X_22–23_–H–X–H) or C_2_HC (C–X_7_–C–X _23_–H–X–C) zinc-finger structure at the C-terminus (Eulgem et al., 2000; Rushton et al., 2010). The WRKY family members are classified into three groups (I, II, and III) based on the number of WDs and the features of their zinc-finger-like motif (Eulgem et al., 2000; Rushton et al., 2010). Group I typically contains two WDs, including a C _2_H_2_zinc-finger structure, whereas Groups II and III are characterized by a single WD, including a C_2_H_2_ and C_2_HC zinc-finger motif, respectively. Group II can be further divided into five subgroups (IIa–IIe) based on phylogenetic analysis of the WDs (Eulgem et al., 2000; Rushton et al., 2010). Members of the WRKY family regulate gene expression by exclusively binding to the W-box (TTGACC/T), which is a *cis*-element in the promoter region of target genes (Bakshi & Oelmüller, 2014; Ulker & Somssich, 2004).

Recent studies have demonstrated that WRKY TFs, as important components of plant signaling web, regulate specific transcriptional programs during plant development, as well as in response to a variety of biotic and abiotic stimuli (Ahuja et al., 2010; Bakshi & Oelmüller, 2014; Rushton et al., 2012; Rushton et al., 2010). For example, 61 of the *PtrWRKY* genes in *Populus* are induced by biotic and abiotic treatments, such as *Marssonina bruuea*, salicylic acid (SA), methyl jasmonate (MeJA), wounding, cold and salinity (Jiang et al., 2014). In rice, expression of *OsWRKY*71 gene is induced by cold stress (Kim et al., 2016), while it also encodes a transcriptional repressor of GA signaling in aleurone cells (Zhang et al., 2004). Five transgenic broccoli lines over-expressing *BoWRKY6* demonstrated significant increase in resistance to downy mildew, with low to very high level of resistance (Jiang et al., 2016). In addition, WRKY TFs are also implicated to modulate plant development, such as seed development and germination (Raineri et al., 2016; Xie et al., 2007; Zhang & Wang, 2011), root growth (Ding et al., 2015; Ranjan & Sawant, 2014), stem elongation (Yu et al., 2012; Zhang et al., 2011), embryogenesis (Jimmy & Babu, 2015; Lagace & Matton, 2004), senescence (Ricachenevsky et al., 2010; Sakuraba et al., 2016), and trichome development (Johnson, 2002).

Wheat (*Triticum aestivum* L.), one of the world’s three main cereals with the highest monetary value (Keating et al., 2014), is affected by multiple environmental stresses, such as salinity, extreme temperature, and especially drought, thus limiting the global production of wheat (Mwadzingeni et al., 2016; Wang et al., 2015). However, the mechanism by which wheat responds to abiotic stress is poorly understood, which might be due to its large genome (approximately 17 GB). The identification and functional characterization of the WRKY family in wheat will contribute to elucidating the mechanism of stress response. Several studies on wheat WRKY identification have been reported in succession. A total of 43 and 92 putative TaWRKYs were previously identified from publicly available expressed sequence tags by Niu et al. (2012) and Zhu et al. (2013), respectively. Okay, Derelli & Unver (2014) characterized 160 TaWRKYs and their expression profiling in RNA-Seq libraries. Recently, Zhang et al. (2016) identified 116 WRKYs, and 13 of them were characterized as senescence-associated genes. Here, we provide extensive insights on TaWRKYs based on the whole genome sequence of wheat. A total of 1113 WRKY TFs were identified in 20 plants representing the nine major evolutionary lineages to gain preliminary insight into the evolution of the WRKY family in Plantae. In addition, we identified 171 TaWRKYs from wheat. Gene classification, physical and chemical parameters prediction, phylogenetic analysis, chromosomal location, duplication events, conserved motif determination, exon–intron structure and *cis-* acting element analysis were employed for the analysis. Finally, gene expression patterns of *TaWRKY* genes in flag leaf, glume, and lemma tissues under water deficit condition were further determined using qRT-PCR. These results will improve our understanding of the WRKY gene family in wheat, as well as contribute to screening more candidate genes for future functional investigation of TaWRKYs under various stresses.

## Materials and Methods

### Database search and identification of WRKYs

The protein sequences of 20 plants from nine different major taxonomic lineages were retrieved from several public databases. All of the amino acid sequences were obtained from the following sources: the eudicots *Arabidopsis thaliana* (At) and *Populus trichocarpa*, the monocots *Brachypodium distachyon*, *Oryza sativa*, *Sorghum bicolor*, *T. aestivum* (Ta), and *Zea mays*, basal magnoliophyta *Amborella trichopoda*, the bryophyte *Physcomitrella patens*, the lycophyte *Selaginella moellendorffii*, the chlorophytes *Ostreococcus lucimarinus*, and the rhodophytes *Cyanidioschyzon merolae* from the Ensembl Plants database (http://archive.plants.ensembl.org/info/website/ftp/index.html); the eudicots *Cucumis sativus* and chlorophytes *Coccomyxa subellipsoidea* C-169, *Micromonas pusilla* CCMP1545, and Volvox carteri and glaucophyte *Cyanophora paradoxa* from the JGI database (PhytozomeV9, http://genome.jgi.doe.gov/pages/dynamicOrganismDownload.jsf?organism=Phytozome#); the chlorophyte *Ostreococcus tauri*, gymnosperm *Picea sitchensis*, and rhodophyte *Galdieria sulphuraria* from NCBI (http://www.ncbi.nlm.nih.gov/protein/). The evolutionary relationship of these 20 species were obtained from NCBI (https://www.ncbi.nlm.nih.gov/Taxonomy/CommonTree/wwwcmt.cgi), and visually displayed by phylogenetic tree using FigTree v1.4.3 (http://tree.bio.ed.ac.uk/software/figtree/).

To identify the WRKY TFs in various species, the HMM profile of the WD (PF03106) downloaded from the Pfam database (http://pfam.xfam.org) (Finn et al., 2016) was applied as a query to search against the local protein database using HMMsearch program (HMMER3.0 software: http://hmmer.janelia.org/) (Finn, Clements & Eddy, 2011) with an *E* value cutoff of 1.0. The sequences obtained were then submitted to the Pfam database to detect the presence of WDs. The protein sequences containing complete or partial WDs, which may be pseudogenes, incomplete assemblies, sequencing errors, or mispredictions (Rinerson et al., 2015) were both considered as putative WRKYs. The physical and chemical properties including number of amino acids (NA), molecular weight (MW), theoretical p*I*, grand average of hydropathicity (GRAVY), aliphatic index (AI), and instability index (II) of putative TaWRKY proteins were calculated using the online ExPASy-ProtParam tool (http://web.expasy.org/protparam/).

### Phylogenetic analysis

MEGA7.0 program was employed to construct the unrooted phylogenetic tree of identified WRKY protein domains in *T. aestivum* L. and *A. thaliana* L. using the maximum likelihood method (Kumar, Stecher & Tamura, 2016). The parameters of the constructed trees were: test of phylogeny: bootstrap (1,000 replicates), gaps/missing data treatment: partial deletion, model/method LG model, rates among sites: gamma distributed with invariant sites (G). Only bootstrap values greater than 60 could be displayed on the tree.

### Chromosomal location of *TaWRKY* genes

To map the locations of *WRKY* gene transcripts in *T. aestivum* L., MapInspect software (http://www.softsea.com/download/MapInspect.html) was employed to visualize the chromosomal distribution of deduced *TaWRKY* genes according to their initial position and length of chromosome. The chromosomal location information of *TaWRKYs* was obtained from Ensembl Plants database (http://archive.plants.ensembl.org/Triticum_aestivum/Info/Index).

To detect the gene duplication, the CDS sequences of *WRKY* genes in wheat were blasted against each other (*E* value <1e^−10^, identity > 90%) (Song et al., 2014). Tandem duplicated *TaWRKY* genes were defined as two or more adjacent homologous genes located on a single chromosome, while homologous genes between different chromosomes were defined as segmental duplicated genes (Bi et al., 2016).

### Characterization of gene structure, conserved motif, and putative *cis*-acting elements

The exon–intron structures of TaWRKY genes were obtained by mapping the CDS to DNA sequences using the Gene Structure Display Server2.0 (http://gsds.cbi.pku.edu.cn/) (Hu et al., 2015). CDS and genomic sequences in *T. aestivum* L. were retrieved from Ensembl Plants database (ftp://ftp.ensemblgenomes.org/pub/release-31/plants/fasta/triticum_aestivum/).

To discover motifs in TaWRKY protein sequences, the online tool Multiple Expectation Maximization for Motif Elication (MEME) 4.11.2 (http://meme-suite.org/) was utilized to identify the conserved motifs in full-length TaWRKYs (Bailey et al., 2009). The optimized parameters were as follows: distribution of motifs, 0 or 1 occurrence per sequence; maximum number of motifs, 10; minimum sites, 6; maximum width 60.

The 1.5-kb upstream of the transcription start site (−1) of all identified *TaWRKY* transcripts was extracted as promoter to predict *cis*-acting elements using the PlantCARE online (http://bioinformatics.psb.ugent.be/webtools/plantcare/html/) (Lescot et al., 2002). Then statistics derived from hits of various *cis*-acting elements in all TaWRKY transcripts were constructed and displayed by diagram.

### Plant materials, water deficit condition, and qRT-PCR

A hexaploid winter wheat (*T. aestivum* L.) cv. Zhengyin1 (St1472/506) was taken in our experiment, carried out from October 2015 to June 2016 in a greenhouse. Seeds were sown in each plastic pot filled with 7 kg of soil, earth-cumuli-orthic Anthrosols collected in northwest China. An equivalent of 0.447 g (urea)/kg^−1^ (soil) and 0.2 g (K_2_HPO_3_)/kg^−1^(soil) were mixed in soil with a net water content of 29.2% at the largest field water capacity. Water control was carried out from anthesis (April 17, 2016). Normal water supply and artificial soil desiccation were implemented with 70% to 75% (control group) and 45% to 50% (moderate water-deficit stress) of the largest field capacity, respectively. Spikes and flag leaves of wheat collected at 0, 1, 3, 5, 10, 15, and 25 days after anthesis (DAA), were immediately frozen in liquid nitrogen and then stored at −80 °C for subsequent analysis. Spikes were separated as glume, lemma, grain, palea, and rachis, and only glume and lemma were used in the experiment.

Total RNA was extracted from wheat tissues using the Trizol reagent (Tiangen, Biotech, Beijing, China) following the manufacturer’s instruction, and then digested with RNase-free DNase I. The quantity and concentration of RNA was evaluated by UV spectrophotometry. The first-strand cDNA was generated using PrimeScriptTM RT Reagent Kit (TaKaRa, Dalian, Liaoning Sheng, China), and the synthesized cDNA products were diluted 1:9 with nuclease-free water to use in qRT-PCR. Primer Primer 5.0 and AllelelID 6.0 (http://www.premierbiosoft.com/index.html) were used to design gene-specific primers (Table S1). Wheat Tublin was used as the reference gene.

The qRT-PCR was carried out using SYBR GreenSYBR^®^ Premix Ex Taq™(TaKaRa, Kusatsu, Shiga, Japan) according to the manufacturer’s instructions with Bio Rad CFX96TM real-time PCR detection system (BioRad, Hercules, CA, USA). Reaction parameters for thermal cycling were: 95 °C for 30 s, followed by 39 cycles of 95 °C for 5 s and 60 °C for 30 s, and finally a melting curve (65 °C to 95 °C, at increments of 0.5 °C) generated to check the amplification. The gene expression levels were calculated with the 2^−ΔΔCT^ method (Livak & Schmittgen, 2001), and three biological replicates were used.

## Results

### Identification of WRKYs in wheat and comparative analysis

**Figure 1:**
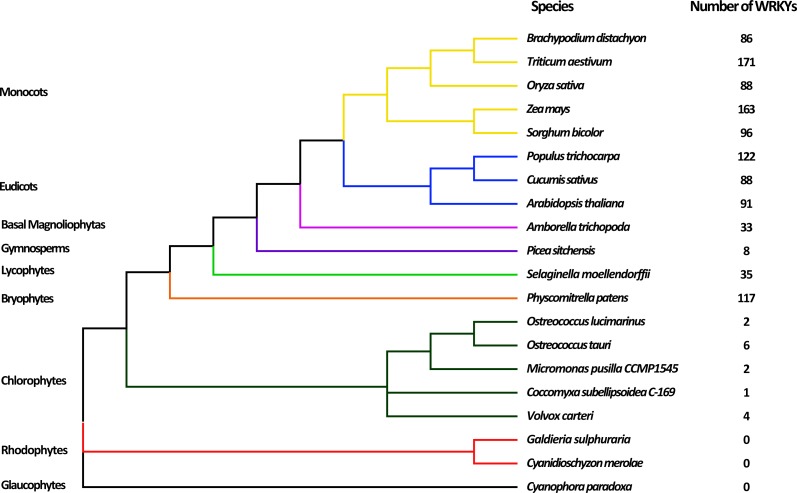
Evolutionary relationship of 20 species among nine lineages within the Plantae. The phylogenetic tree was constructed based on the evolutionary relationship of 20 species obtained from NCBI (https://www.ncbi.nlm.nih.gov/Taxonomy/CommonTree/wwwcmt.cgi) using FigTree v1.4.3. The numbers of putative WRKYs in each species are listed next to the tree.

To comprehensively analyze and identify WRKY TFs in plants, 20 plants representing the nine major evolutionary lineages were chosen for analysis. After searching by HMMER and detecting WDs by Pfam database, a total of 1113 WRKY TFs were obtained (Table S2). The evolutionary relationships of various species and the number of WRKY TFs are shown in Fig. 1. Most terrestrial plants, including Monocots, Eudicots, and Bryophytes, contained 86 to 171 WRKY proteins, while *Picea sitchenis*, belonging to the Gymnosperms, carried only eight WRKY TFs, which could be due to incomplete sequencing. However, six or less WRKY proteins were found in aquatic algae, of which no WRKY TFs were found in Rhodophytes and Glaucophytes. In general, the number of WRKY TFs in many higher plants was more than that in lower plants, which suggested that the WRKY TFs may play an important role in the process of plant evolution. The number of WRKY proteins increased as plants evolved, possibly because of genome duplication.

**Figure 2:**
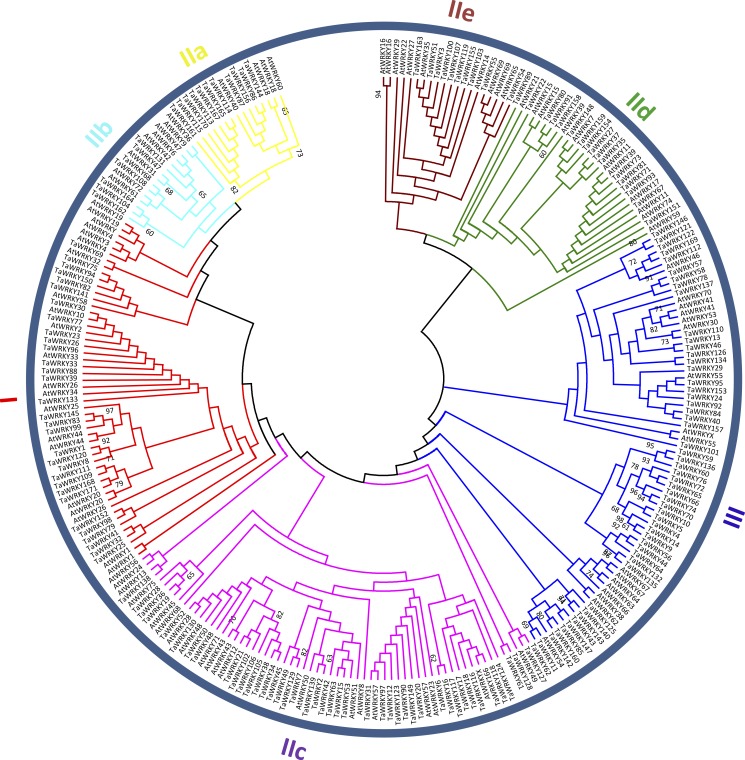
Phylogenetic tree of WRKY domains (WDs) from wheat and *Arabidopsis*. The unrooted maximum-likelihood (ML) tree was constructed based on the WDs from wheat (171) and *Arabidopsis* (91) using MEGA7.0 with 1,000 bootstrap replicates. Branches with less than 60% bootstrap support were collapsed. The names of groups (I , II a–e, and III) are shown outside of the circle. Branch lines of subtrees are colored, indicating different WRKY subgroups.

In wheat, a total of 174 WRKY proteins were searched using the HMM search program. Subsequently, all obtained sequences were verified by Pfam database, which resulted in the identification of 171 WRKY TFs (Table S3). Based on their chromosome locations, we named 115 TaWRKYs as TaWRKY1 to TaWRKY115, and another 56 sequences were called TaWRKY116 to TaWRKY171 as they were anchored in the scaffolds. Among the 171 TaWRKYs, manual inspection showed that some were partial sequences, in which WDs or zinc-finger structures were incomplete or nonexistent.

The parameters used to describe the TaWRKY proteins were shown in Table S3. Molecular weight, theoretical p*I*, and aliphatic index could not be computed in sequences containing several consecutive undefined amino acids. The lengths of TaWRKY proteins ranged from 44 (TaWRKY121) to 1,482 residues (TaWRKY78), whereas the PI ranged from 4.96 (TaWRKY164) to 10.73 (TaWRKY40). This suggested that different TaWRKYs might operate in various microenvironments (Wang et al., 2014). The values of grand average of hydropathicity were all negative, which indicated that TaWRKY proteins were all hydrophilic. Almost all TaWRKYs were defined as unstable proteins, and only 30 TaWRKYs with instability index less than 40 were considered to be stable proteins.

### Classification and phylogenetic analysis of TaWRKYs

To categorize and investigate the evolutionary relationship of the TaWRKY proteins in detail, we constructed an unrooted maximum-likelihood phylogenetic tree with 262 putative WDs in *Arabidopsis* and wheat (Fig. 2). Basing on the classification of AtWRKYs and primary amino acid structure feature of WRKY (Eulgem et al., 2000), we classified TaWRKYs into three major groups (Groups I, II, and III). The 30 TaWRKYs possessing two WDs and C _2_H_2_-type zinc finger motifs (C–X_3–4_–C–X_22–23_–H–X_1_–H) were classified into group I. Group II comprised 95 sequences, and each protein contained a single WD and C_2_H_2_-type zinc finger structure (C–X_4–5_–C–X_23_–H–X _1_–H). We further divided Group II into five subgroups, including IIa, IIb, IIc, IId, and IIe with 11, 7, 50, 17, and 10 members, respectively. Finally, 45 TaWRKYs with a single WD were assigned to Group III because of their C_2_HC zinc-finger structure (C–X_6–7_–C–X_23–28_–H–X _1_–C).

As shown in Table S3, besides the highly conserved WRKYGQK motifs, we found three variants in TaWRKYs, namely WRKYGKK (10), WRKYGEK (11), and WSKYGQK (1), which were distributed in subgroup IIc, III, and TaWRKY157, respectively. In addition, two zinc-finger form variants, C–X_6_–P–X_23_–H–X–C and C–X6–F–X23–H–X–C were identified in TaWRKY80 and TaWRKY166, respectively. TaWRKY157, the most unique among all putative TaWRKYs, contained two WDs but with C_2_HC-type zinc finger structure (C–X_7_–C–X_23–_–H–X_1_–C). The “Group I Hypothesis” sees all WRKY genes evolving from Group I C-terminal WDs (Rinerson et al., 2015). Therefore, TaWRKY157 could preliminarily be taken as an intermediate member of Groups I to III, although it was classified into Group III in the phylogenetic tree.

In this study, Group II was found to be the largest group of WRKY TFs in wheat*.* The members of Group II accounted for approximately 55.6% of all putative TaWRKYs, which was consistent with *Musa balbisiana* (Goel et al., 2016), pepper (Diao et al., 2016), and soybean (Song et al., 2016a). Subgroups IIa and IIb were separated from one clade, and IId and IIe were clustered to a branch, which is similar to previous studies in wheat (Okay, Derelli & Unver, 2014; Zhu et al., 2013).

### Chromosomal location of *TaWRKY* genes

**Figure 3:**
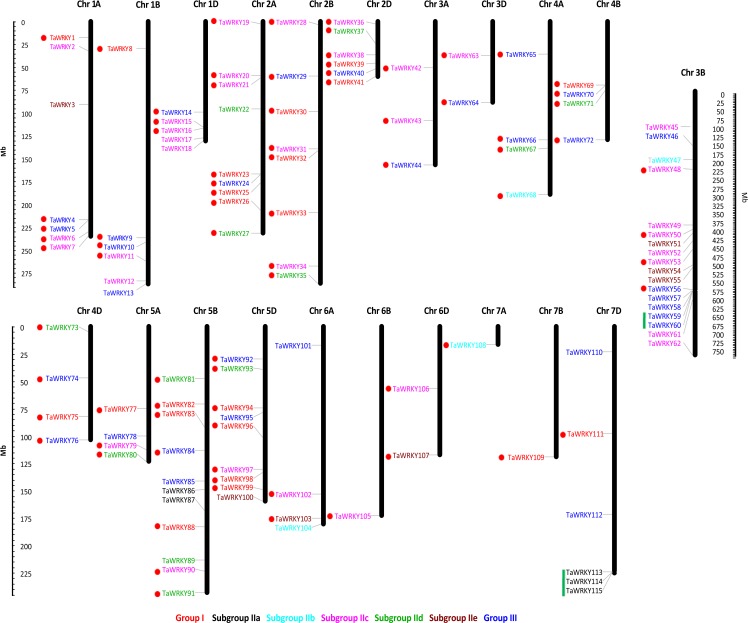
Chromosome distribution of Ta*WRKY* genes. The chromosomal position of each *TaWRKY* was mapped according to the wheat genome. The chromosome numbers were shown at the top of each chromosome. Fifty-six *TaWRKYs* on the scaffold (*TaWRKY115–171*) could not be anchored onto any specific chromosome. The location of each *WRKY* gene was indicated by a line. The scale is in mega bases (Mb). The green lines indicated the tandem duplication genes, and the segmental duplicated genes were shown with red dots.

Among the 171 *TaWRKY* genes, 115 were mapped onto the 21 wheat chromosomes, and the other 56 were anchored in the scaffolds (*TaWRKY116–171*) (Table S3, Fig. 3). More *TaWRKY* genes were relatively distributed in Chromosomes 3B (18, 15.7%), 5B (11, 9.57%), 2A (9, 7.8%), and 5D (9, 7.8%). In contrast, chromosomes 6B, 7A, and 7B contained only one *TaWRKY* gene (0.870%). In general, most identified *TaWRKY* genes were observed in distal regions of chromosomes and only a few were observed in proximal regions. This phenomenon suggested that the *TaWRKY* genes were mapped on the all chromosomes with a significantly non-random and uneven distribution. The *TaWRKY* genes density in each chromosome ranged from 0.004/Mb (7B) to 0.056/Mb (5D) (Fig. S1).

Duplication events of *WRKY* genes have been found universally in a number of plants, such as peanut (Song et al., 2016b), white pear (Huang et al., 2015), and *Brassica napus* (He et al., 2016). In this study, we identified 79 *TaWRKY* gene duplication pairs which corresponded to 85 genes (Table S4, Fig. 3). This phenomenon indicated that some of the *TaWRKY* genes have more than one duplicated gene, which could be due to the multiple rounds of whole genome duplication in wheat. As shown in Fig. 3, two WRKY tandem duplication clusters (*TaWRKY59*/*TaWRKY60*, *TaWRKY113*/*TaWRKY114*/*TaWRKY115*) were identified on chromosomes 3B and 7D, respectively. In addition, 80 genes were found to have undergone segmental duplication, which were paralogs of *WRKY* genes on different chromosomes (Bi et al., 2016).

### Gene structure analysis of *WRKY* genes in wheat

The exon–intron distribution was analyzed to further detect structural features of *TaWRKY* genes. Figure S2 showed that the number of introns in TaWRKY family genes varied from 0 to 5, while 0 to 8 in rice (Xie et al., 2005) and 0 to 22 in *Musa acuminate* (Goel et al., 2016), respectively. This phenomenon suggested that *WRKY*s in wheat show lower gene structure diversity. A total of 72 (42.11%) *TaWRKY* genes with two introns accounted for the largest proportion, followed by 44 (25.73%), 23 (13.45%), 15 (8.77%), 14 (8.19%), and 3 (1.75%) genes, possessing 1, 3, 4, 0, and 5 introns, respectively. The distribution pattern of introns and exons was group specific, which was similar to cassava (Wei et al., 2016) and carrot (Li et al., 2016), and *TaWRKY* gens belonging to the same subfamily shared a similar exon–intron structure. For example, *TaWRKYs* in Group III contained 0–5 introns, while approximately 91.11% (41/45) possessed 1–2 introns.

Two types of introns (V-type and R-type) were located in the WD characterized based on their splice site (Bi et al., 2016; Wang et al., 2014; Xie et al., 2005). V-type introns (phase 0) have a splice site before the V (valine) residue in C_2_H_2_ zinc finger structure, and R-type introns (phase 2) on the R (arginine) residue of the WD (Bi et al., 2016; Wang et al., 2014; Xie et al., 2005). In our study, all of the *TaWRKY* genes (17) in Groups IIa and IIb only contained V-type introns except *TaWRKY165*, which had no intron. However, R-type introns were mostly observed in all the other groups (Groups I, IIc, IId, IIe, and III) (Fig. S2). This phenomenon indicated that the intron phases were significantly conserved within the same group but remarkably different between groups (Chen, 2014). These results provided additional evidence to support the phylogenetic groupings and *TaWRKYs* classification.

### Motif composition analysis of TaWRKYs

**Figure 4:**
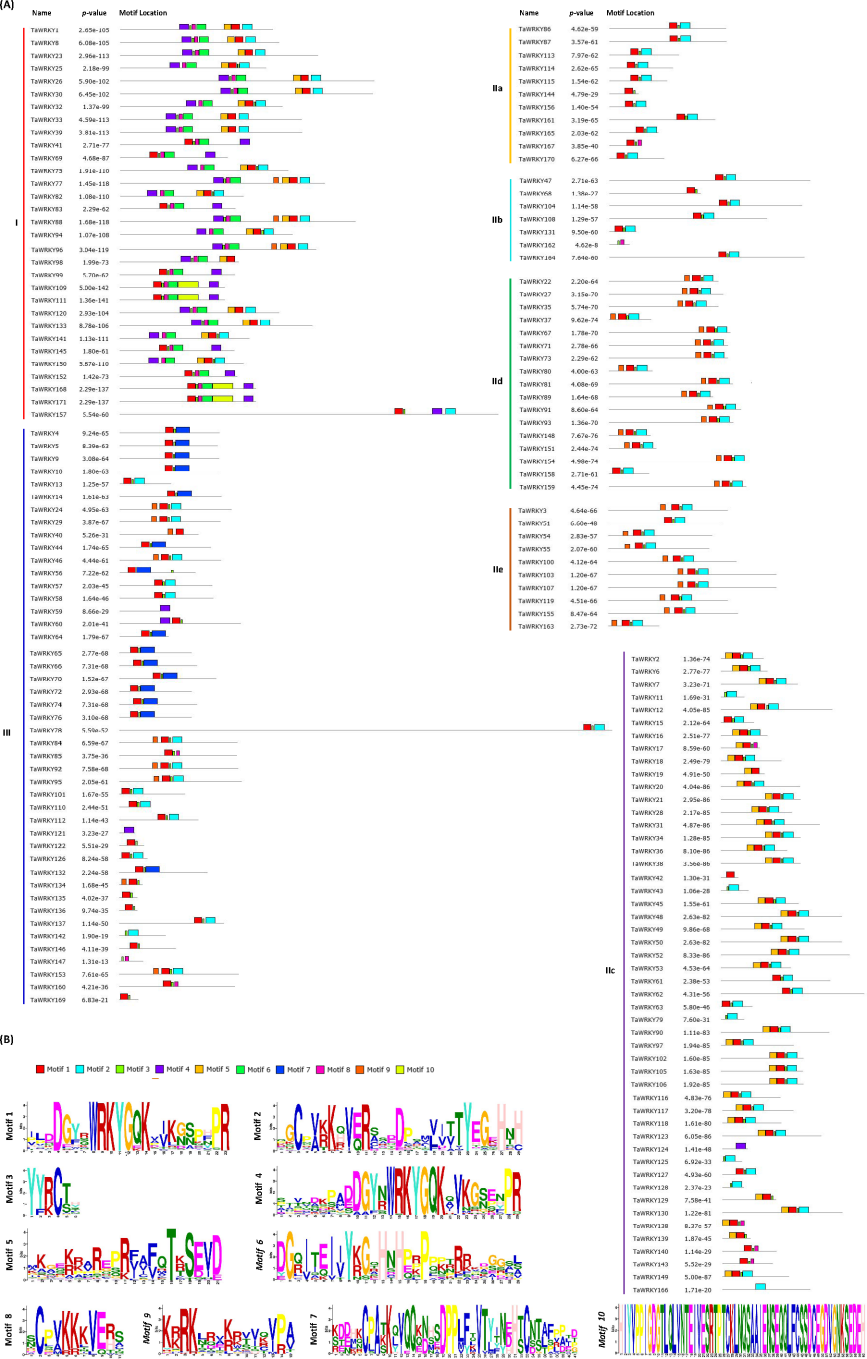
Schematic diagram of conserved motifs in TaWRKYs. (A) Distribution of conserved motifs of TaWRKYs from different groups and subgroups. The conserved motifs represented with boxes in the TaWRKY proteins using MEME. Box size indicated the length of motifs. Gray lines represented the non-conserved sequences. (B) Logo of each motif. The motifs, numbered 1–10, were displayed in different colored boxes.

The conserved motifs of WRKY proteins in wheat were analyzed to explore the similarity and diversity of motif compositions. A total of 10 distinct motifs, named motifs 1–10, were detected using the MEME online program (Fig. 4). Among these 10 motifs, motifs 1 and 4 contained a WRKYGQK sequence, which is a basic feature of TaWRKYs. At least one of them contained almost all deduced TaWRKYs, except several incomplete proteins, such as TaWRKY11, 43, and 162. Motif 1 was observed almost in all groups, whereas motif 4 dispersed in Group I mostly.

As displayed schematically in Fig. 4, TaWRKYs within the same group or subgroup shared similar motif compositions. For instance, motifs 6 and 10 were unique to Group I, whereas motif 7 is specific to Group III. The motif unique to a particular group is likely to be involved in specific biological process in plants. Therefore, each family or subfamily of WRKY genes might be responsible for the specific biological process (Goel et al., 2016; Lippok et al., 2003). Furthermore, members of subgroups IIa and IId showed almost identical motif distribution patterns, indicating functional similarity among them. Interestingly, these two subgroups were also clustered to a branch in the phylogenetic tree. Likewise, the same phenomenon was also observed in subgroup IId and IIe. These results further validated the categorization of TaWRKYs and phylogenetic relationships.

**Figure 5:**
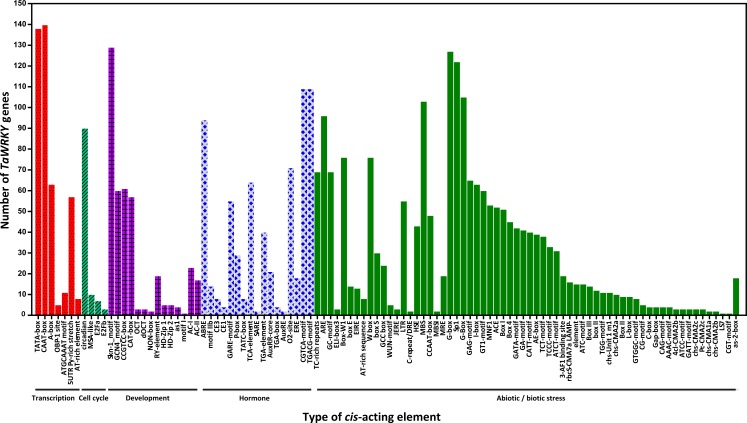
Number of *TaWRKY* genes containing various *cis*-actingelements. The *cis*-acting elements were identified with the online PlantCARE program using the 1.5-kb upstream from the transcription start site of *TaWRKY* genes. A graph was generated based on the presence of *cis*-acting elements responsive to specific elicitors/conditions/processes (*x*-axis) in WRKY gene family members ( *y*-axis).

### Variety of *cis*-acting elements in promoter regions of wheat *WRKY*  genes

*cis*-acting elements in the promoter are crucial to gene expression, which is an essential part of its function (Dehais, 1999; Lescot et al., 2002). The 1.5 kb upstream promoter regions of all *TaWRKYs* were used to predict *cis-* acting elements using the online database PlantCARE. Here, various *cis-* acting elements were found in 142 out of 171 *TaWRKY* genes, while the remaining WRKYs could not be detected because of short sequence in their upstream regions (Table S5 , Fig. 5). Many *cis-* acting elements were related to response of hormones and biotic stresses, including MeJA, abscisic acid (ABA), SA, gibberellins (GA), auxin, zein, and fungus. MeJA-responsive elements with the largest portion were found in the promoter regions of 109 *TaWRKY* genes. Additionally, some elements involved in various abiotic stresses, such as light, wound, cold, heat, anaerobic induction, and drought, were identified in a large number of *TaWRKY* genes. A total of 44 light-responsive elements were almost distributed in all of the *TaWRKY*s. Some elements also observed in genes may regulate expression of different tissues (seed, root, shoot, leaf, phloem/xylem, endosperm, and meristem) in wheat development. Interestingly, a total of 76 *TaWRKY*s contained W-box (TTGACC), which regulates gene expression by binding WRKY, indicating these genes may auto-regulated by itself or cross-regulated with others (Chi et al., 2013; Jiang et al., 2014). MBSI, a MYB binding site involved in flavonoid biosynthetic genes regulation, only existed in *TaWRKY87* and *TaWRKY142*, which suggested that these two genes may regulate flavonoid metabolism. Two unique genes were found, *TaWRKY58* and *TaWRKY94*, which might respond to cold and water-deficit stresses for containing a cold and dehydration responsive element, C-repeat/DRE. Another special MYB binding site MBS that participated in drought response, were identified in 103 genes, indicating that most *TaWRKY*s seem to be involved in drought stress response (Table S5). Notably, all members analyzed contained more than one *cis*-element. Our analysis and previous studies both suggested that *TaWRKY* genes are involved in transcriptional regulation of plant growth and stress responses (Bakshi & Oelmüller, 2014; Ding et al., 2015; Raineri et al., 2016; Rushton et al., 2010).

### Expression profiles of *TaWRKY* genes under water-deficit condition

**Figure 6:**
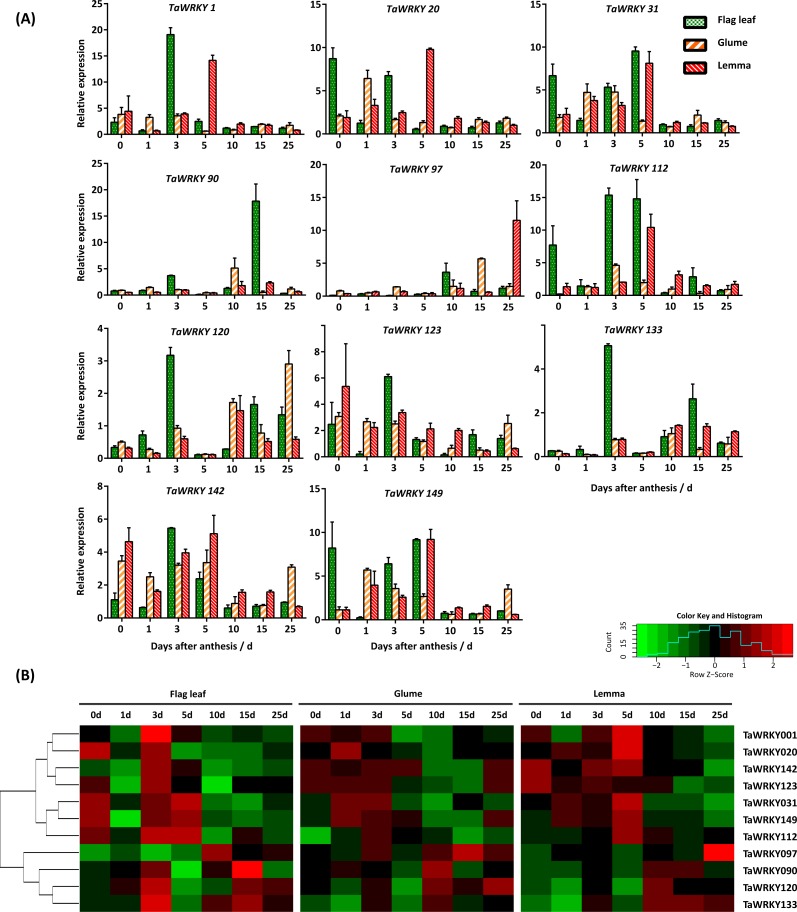
Expression pattern of *TaWRKY* genes in flag leaves, glumes, and lemmas during the grain-filling stage under drought stress. (A) Bar graphs showing the relative expression values of each *TaWRKY* genes after drought treatment. Samples were collected 0, 1, 3, 5, 10, 15, and 25 DAA, from which water control was performed. The mean ± SE of three biological replicates are presented. Relative fold changes were obtained by qRT-PCR using the 2^−ΔΔCT^ method. (B) Heatmap showing the expression profile of *TaWRKYs* in flag leaves, glumes, and lemmas under drought stress. Heatmap was generated based on log2-transformed count value from three replicates of qRT-PCR data using R language. Red and green boxes indicated high and low expression levels of genes, respectively.

With the exception of two shorter sequences (*TaWRKY122 and 169*), 12 out of 171 transcripts were selected as candidate drought responsive genes according to their orthologous *WRKYs* in *Arabidopsis*, which are involved in water deprivation, using the Biomart (http://plants.ensembl.org/index.html) (Table S6). The *AtWRKYs* responding to water-deficit stress were obtained based on function annotation in the TAIR database (http://www.arabidopsis.org/index.jsp). To validate these candidate drought-response genes, we determined their expression pattern in flag leaves, glumes, and lemmas using qRT-PCR. In our study, expression of genes could be detected at the transcript level in almost all selected tissues during the grain-filling period except *TaWRKY8* (Fig. 6).

As shown in Fig. 6, we found that *TaWRKY* genes in glumes and lemmas share a more similar expression pattern compared with that in flag leaves. A relatively large group of genes, including *TaWRKY1, 20, 31, 112, 123, 142,* and *149* were significantly up-regulated in flag leaves at 0, 3, or 5 DAA, which suggested that these genes were highly induced at the early grain-filling stage (0–8 DAA). Among them, some genes, like *TaWRKY123* and *142*, were slightly up-regulated initially and then were restrained followed by an increase in the last point under water-deficit condition in glumes. In addition, peaks in the expression of several members (*TaWRKY1, 20, 123,* and *142*) were mostly found at 5DAA in lemma, which lags behind other two tissues. Furthermore, water-deficit stress induced the most rapid up-regulation of some genes, and showed differences in three tissues. For example, both *TaWRKY31* and *TaWRKY149* were induced quickly in flag leaves after the onset of the water-deficit stress (0 DAA), approximately increasing up 6.67- and 8.22-fold, respectively. However, later induction was observed in glumes and lemmas. The immediate transcription response observed upon water-deficit stress appeared to be related to a more rapid perception of the drought (Eulgem et al., 2000; Rushton et al., 2010). Genes in another interesting cluster, composed of *TaWRKY90, 97, 120,* and *133*, were down-regulated or slightly changed in glumes and lemmas during the early grain-filling stage, and induced during the middle (9–15 DAA) or late grain-filling stage (16–25 DAA) under water-deficit stress. However, two genes (*TaWRKY120, 133*) in flag leaves were strongly induced 3 days after water-deficit stress. This phenomenon indicated that *TaWRKY120* and *TaWRKY 133* genes were predominantly expressed in flag leaves at the early-filling stage. Our data suggested that the tissue-specific expression of *TaWRKYs* existed in wheat, and it appeared to be consistent with their role in tissues.

## Discussion

WRKY TFs are one of the largest families of transcriptional regulators in plants, and form integral parts of signaling webs that regulate many plant processes (Rushton et al., 2010). Although some investigations on wheat WRKYs have been reported in succession, characterization and functional annotation information about TaWRKYs was still insufficient. Niu et al. (2012) identified 43 putative TaWRKYs, named TaWRKY1 to TaWRKY43, which were represented with the same names in another study performed by Okay, Derelli & Unver (2014). Zhu et al. (2013) identified 92 TaWRKYs from the NCBI dbEST and/or the DFCI gene index, and constructed a phylogeny map. A total of 160 TaWRKYs were characterized according to their HMM profiles, conserved domains, distribution among WRKY groups, and phylogenetic relationships, and some drought responsive members were validated in leaf and root tissues (Okay, Derelli & Unver, 2014). Recently, Zhang et al. (2016) identified 116 WRKYs, and 13 of them were characterized as senescence-associated genes.

Our present study improves our understanding of WRKYs in wheat, and provides a more comprehensive insight based on the wheat genome. We identified WRKYs in 20 species and characterized 171 wheat WRKYs in terms of gene classification, physical and chemical parameters prediction, phylogenetic analysis, chromosomal location, duplication events, conserved motif determination, exon–intron structure, and *cis-* acting element analysis, which might help to screen candidate stress-responsive genes in wheat for further study.

To understand the evolution of WRKY TF family, we identified a total of 1,113 WRKY proteins in wheat and other 19 species representing the nine major plant lineages. Interestingly, the number of WRKY TFs in many higher plants was more than that in lower plants, which implied that the WRKY TFs might play significant roles during evolution from simpler unicellular to more complex multicellular forms. The whole genome duplication can result in divergence and formation of species over time, accompanied with retention or loss of some duplicated genes (Dehal, 2005). Thus, we could preliminarily speculate that the number of WRKY proteins increased as plants evolved possibly because of genome duplication (Li et al., 2016).

Compared with the species analyzed in this study, the wheat (*T. aestivum* L.) genome contained the highest number of WRKY TFs (171). The expansion of *WRKY* gene family in wheat might be due to the following reasons: (1) *T. aestivum* L. is an allohexaploid, and originated from two recent hybridizations between three diploid progenitors, donors of the A, B, and D subgenomes (Glover et al., 2015). *T. aestivum* L. genome experienced the whole genome duplication events after two hybridizations at approximately 0.8 and 0.4 million years ago, respectively (Glover et al., 2015), which would produce a large number of paralogs (Conant & Wolfe, 2008). (2) Likewise, small-scale gene duplication, including segmental and tandem duplication (Zhu et al., 2014), might also be significant in the evolution of *WRKY* gene family in wheat. The origin of new genes during evolution is also dependent on gene duplication (Ohno, 1970). Gene duplication allows essential genes to undergo mutations in the duplicated copy, suggesting that similar genes would diverge over the long evolutionary time period, and then improve the expansion and evolution of the gene family (Conant & Wolfe, 2008; De, Lanave & Saccone, 2008). Segmental and tandem duplication events have been reported widely in different species. For examples, 16 MADS-box genes are located within the duplicated segments of the rice genome, and 20 seem to have evolved from tandem duplication (Arora et al., 2007); 96 *MATE* genes with tandem duplications and 70 with segmental duplications were observed in soybean, which contributed largely to the expansion of MATE family in the soybean genome (Liu et al., 2016). The current investigation showed that 85 of 171 (49.7%) *TaWRKY* genes evolved from either tandem or segmental duplication. Interestingly, 80 of *WRKY* genes were segmentally duplicated and only five were tandemly duplicated, implying that high segmental and low tandem duplications existed in *TaWRKY* genes, consistent with white pear (Huang et al., 2015), grapevine (Wang et al., 2014), and soybean (Song et al., 2016a). Our results showed that the number of duplicated genes was mainly determined by segmental events because genes generated from segmental duplication have more chances to be retained due to subfunctionalization or neofunctionalization (Huang et al., 2015; Lynch, 2000; Moore & Purugganan, 2005; Wang et al., 2005). Therefore, although tandem duplication contributed to the expansion of TaWRKY family, segmental duplication probably played a more pivotal role (Zhu et al., 2014). (3) The expansion of gene families along a specific lineage can be due to chance or the result of natural selection. Adaptive expansion of gene families occurs when natural selection would favor additional duplicated genes (Demuth & Hahn, 2009).

Increasing research suggests that the WRKY TFs are involved in various biological processes, including plant development, and responses to biotic and abiotic stresses (Eulgem & Somssich, 2007; Liu et al., 2015; Luo et al., 2013; Rushton et al., 2010; Zhao et al., 2015). Plant hormones, as essential endogenous signal molecules within the plant, can regulate cellular processes, plant growth, and development under severe stress conditions (Grove et al., 1979; Kermode, 2005; Ryu & Cho, 2015). Considerable evidence indicated that the expression of *WRKY* genes was affected after hormone treatment (Jiang et al., 2014; Yang et al., 2009). In wheat, a large amount of *cis-* acting elements responding to phytohormones, such as MeJA, ABA, SA, GA, etc., were detected in *TaWRKY* genes. This phenomenon suggested that these *WRKY* genes might regulate growth and development of wheat by functioning as key factors in regulating specific signaling pathways. In addition, WRKY TFs were involved in responses to abiotic stresses. For instance, *TaWRKY44* in transgenic tobacco confers multiple abiotic stress tolerances, including drought, salt, and osmotic stress (Wang et al., 2015). Twelve *GmWRKY* genes were differentially expressed under salt stress (Song et al., 2016a). In this work, a large number of *TaWRKY* genes contained several *cis*-acting elements associated with abiotic stresses, such as light, wound, cold, heat, anaerobic induction, and drought, implying that a number of *WRKY* genes in wheat participate in various abiotic stresses. In general, the results indicated that most *TaWRKY* genes were involved in multiple biotic and abiotic stresses, which was consistent with previous studies (Eulgem & Somssich, 2007; Jiang et al., 2014).

Drought is one of the most significant stresses resulting in reduction of wheat production (Keating et al., 2014). Enhancement of grain yield stability under water-deficit stress can be achieved initially by maximizing soil water capture through the root system (Blum, 2009). The grain-filling, an important process in yield formation, is mainly sustained by photosynthesis of flag leaves and spikes under drought treatment, and photosynthesis of spike is less sensitive to drought than that in flag leaves (Jia et al., 2015; Tambussi, Nogues & Araus, 2005). However, the investigation on *TaWRKY* genes was mostly focused on root and leaves, and limited in spikes. In this study, we determined the relative expression of *TaWRKY* genes in flag leaves, glumes, and lemmas during the grain-filling period upon water deficit using qRT-PCR. Obvious differences of gene expression pattern between flag leaves and spikes (glumes and lemmas) were observed in wheat. For example, *TaWRKY142* were up-regulated at 3 DAA in flag leaves, but induced in glumes and lemmas since the imposition of the water-deficit stress and maintained up-regulation during the early grain-filling stage, suggesting the putative role of *TaWRKY142* gene in spike tissues. In addition, *WRKYs* belonging to a group do not necessarily share a similar expression pattern as their roles are different in the physiology (Li et al., 2015). For instance, *TaWRKY1, 120,* and *133*, the orthologs to *AtWRKY3,* all belong to Group I. Both *TaWRKY120* and *TaWRKY133* were upregulated at 3DAA and the middle to late grain filling stage in flag leaves, while induction of TaWRKY1 was only observed at 3DAA, which indicated their different functions in the same tissue. The similar phenomenon was also reported in *Salvia miltiorrhiza*. Five genes (*SmWRKY2, 24, 39, 54,* and *55*), belonging to Group I, were predominantly expressed in roots, whereas the other Group I members, including *SmWRKY42, 13,* and *60* were mainly expressed in stems, leaves, and flowers, respectively (Li et al., 2015).

## Conclusions

In this study, we identified 171 TaWRKYs from the whole wheat genome. The phylogenetic relationship, classification, gene structure, composition of conserved motif, chromosomal location, and *cis*-acting elements were systematically analyzed. The expansion of the WRKY gene family in wheat was mainly due to gene duplication, and compared with tandem duplication, segmental duplication might play a more pivotal role. The *cis-* acting elements analysis suggested that most *TaWRKY* genes were involved in various processes during growth and development as well as stress responses in wheat, which will provide abundant resources for functional characterization of *TaWRKY* genes. Expression analysis showed that almost all *TaWRKY* genes validated in our experiment were involved in response to water-deficit stress. Comparing with flag leaves, we found that glumes and lemmas share a more similar expression pattern, and the tissue-specific expression of *TaWRKYs* existed in wheat. Taken together, our results will provide a more extensive insight on *TaWRKY* gene family, and also contribute to screen more appropriate candidate genes for further investigation on function characterization of WRKYs under various stresses.

##  Supplemental Information

10.7717/peerj.3232/supp-1 Figure S1 Chromosomal distribution of *WRKY* genes in wheat Bar graphs indicated the number of *TaWRKY* genes in each chromosome, and broken lines indicated the *WRKY* gene density per chromosome in wheat.

10.7717/peerj.3232/supp-2 Figure S2 Gene structure of *WRKY* gene family in wheat (A) The unrooted maximum-likelihood (ML) phylogenetic tree of TaWRKYs. The tree was constructed based on the WDs from wheat by MEGA 7.0 with 1000 bootstrap replicates. (B) Exon—intron composition of *TaWRKY* genes. (C) Intron number of each *TaWRKY* genes. See legends for detailed information.

10.7717/peerj.3232/supp-3 Table S1 List of primers for qRT-PCR experiments

10.7717/peerj.3232/supp-4 Table S2 WRKY TF family members in plants Raw data

10.7717/peerj.3232/supp-5 Table S3 Sequence features of WRKYs identified in wheat

10.7717/peerj.3232/supp-6 Table S4 Segmental and tandem duplication gene pairs identified in * TaWRKYs*

10.7717/peerj.3232/supp-7 Table S5 Putative *cis*-acting elements identified in the promoter regions of *TaWRKYs*

10.7717/peerj.3232/supp-8 Table S6 Drought responsive AtWRKYs in *Arabidopsis* and their putative orthologous *TaWRKYs* in wheat

## Additional Information and Declarations

### Data Availability

The following information was supplied regarding data availability: The raw data has been supplied as a Supplementary File.
