# Supplementary figures and images for: Genome-wide analysis of WRKY transcription factors in wheat (Triticum aestivum L.) and differential expression under water deficit condition
Source: PeerJ. 2017 May 4;5:e3232. doi: 10.7717/peerj.3232 (PMC5420200; doi:10.7717/peerj.3232)

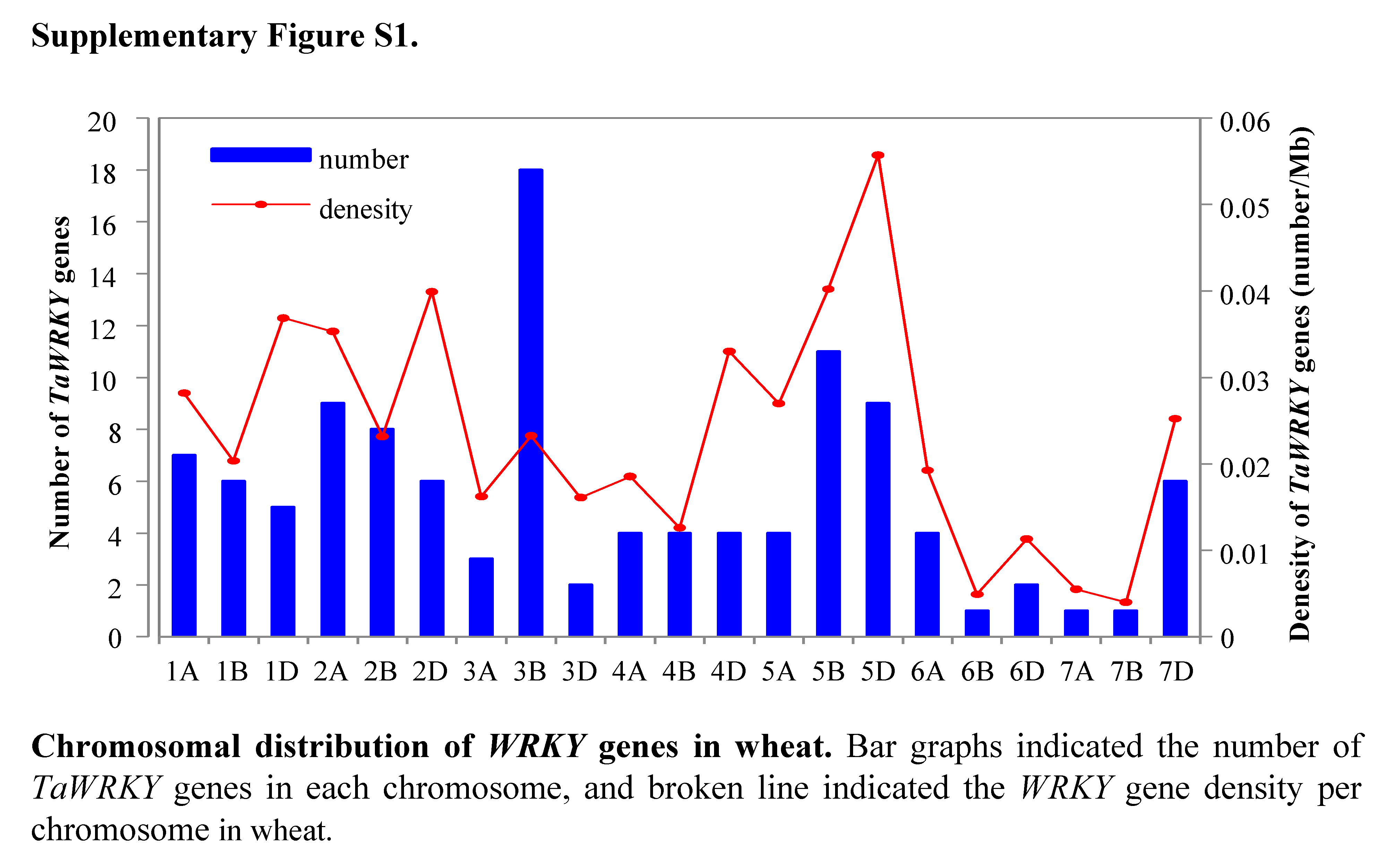

Supplement: Figure S1 — Bar graphs indicated the number of TaWRKY genes in each chromosome, and broken lines indicated the WRKY gene density per chromosome in wheat. [file peerj-05-3232-s001.png]
