# Supplementary material for: Genome-wide analysis of WRKY transcription factors in wheat (Triticum aestivum L.) and differential expression under water deficit condition
Source: PeerJ. 2017 May 4;5:e3232. doi: 10.7717/peerj.3232 (PMC5420200; doi:10.7717/peerj.3232)
Supplement: Table S1 [file peerj-05-3232-s003.pdf]

Supplemental Table S1. List of primers used for qRT-PCR experiments.

Transcript ID	Proposed name	Forward primer (5'→3')	Reverse primer (5'→3')
Traes_1AS_F3EAEC435.1	TaWRKY1	AGTTCCCTGCTATTCCATCTAAG	TTCTCGCTCGCCATCTCC
Traes_1BS_EF67E5A24.1	TaWRKY8	CAAGCCGCAAGTGACATTATC	TCGTTCAGCCTCCTTTATTCC
Traes_2AL_1B43EA59E.1	TaWRKY20	CACCACCACCACCACCTC	AGCAGCGACGACGACATC
Traes_2BL_A69F6C5DF.1	TaWRKY31	GCACACCACCACCACCTC	AGCAGCGACGACGACATC
Traes_5BL_C1D6B6B74.2	TaWRKY90	ACGAACTGCGAAGGTCAAC	TCCTGGAGGCACGATGTC
Traes_5DL_7E2053226.2	TaWRKY97	TCATCGTCATCGTTGGTTCC	GCTTGGTTATCCGCCTTCC
Traes_7DL_A9EF00572.1	TaWRKY112	GCACAGGTAGTCGCCAAC	AGCACAAGCACGCAGTAG
Traes_1DS_A6733B734.1	TaWRKY120	CTCTTTGGCTTCTCCTTTCAC	CTCTTGTTGCTCACTTCTACC
Traes_2DL_04535D371.1	TaWRKY123	GCACACCACCACCACCTC	AGCAGCGACGACGACATC
Traes_3B_990298FF5.1	TaWRKY133	GGCTTCAACGGCAACTTCG	ATGTCCTCCTCCCTCGGCTC
Traes_4AS_0DA136E0E.1	TaWRKY142	GCAAGCGAGATACTGACACG	GCAGCCTTGGTCTGATTTGT
Traes_5AL_06A6F9328.2	TaWRKY149	TTGTTTATTCTTCTGTCCCCTTTC	AACTTCGCTCTTGGTCACG
