# Supplementary material for: Genome-wide analysis of WRKY transcription factors in wheat (Triticum aestivum L.) and differential expression under water deficit condition
Source: PeerJ. 2017 May 4;5:e3232. doi: 10.7717/peerj.3232 (PMC5420200; doi:10.7717/peerj.3232)
Supplement: Table S2 — Raw data [file peerj-05-3232-s004.pdf]

Supplemental Table S2. The WRKY transcription factor family members in plants.

The data were downloaded from Ensembl Plants (http://plants.ensembl.org/info/website/ftp/ index.html), PhytozomeV9 (http:/genome.jgi.doe. gov/pages/dynamicOrganismDownload. jsf?organism=PhytozomeV9) and NCBI (https://www.ncbi.nlm. nih. gov/ protein/).

Lineages	Transcript ID	Alignment		Envelope		Hmm name	Hmm		Hmm length	Bit score	E-value	Number of WRKY Domains
		Start	End	Start	End		Start	End				
**Monocots**	***Brachypodium distachyon* (86 WRKYs)**											
	BRADI1G02327.1	119	174	118	174	WRKY	2	60	59	85.6	1.9E-24	2
		276	330	276	331	WRKY	1	59	59	80.5	7.8E-23	
	BRADI1G07970.1	191	247	191	247	WRKY	1	60	60	76.5	1.3E-21	2
		324	381	324	381	WRKY	1	60	60	83.1	1.2E-23	
	BRADI1G08106.1	130	187	130	187	WRKY	1	60	60	90.9	4.3E-26	1
	BRADI1G08106.2	130	187	130	187	WRKY	1	60	60	90.9	4.3E-26	1
	BRADI1G09170.1	357	413	356	414	WRKY	2	59	58	91.8	2.2E-26	1
	BRADI1G13207.1	42	97	42	98	WRKY	1	59	59	73.5	1.1E-20	1
	BRADI1G14300.1	276	332	275	333	WRKY	2	59	58	94.9	2.5E-27	1
	BRADI1G14300.2	276	332	275	333	WRKY	2	59	58	94.9	2.5E-27	1
	BRADI1G14300.3	275	331	274	332	WRKY	2	59	58	94.9	2.5E-27	1
	BRADI1G16120.1	206	262	206	262	WRKY	1	60	60	82.8	1.5E-23	2
		375	432	375	432	WRKY	1	60	60	83.7	7.4E-24	
	BRADI1G17660.1	128	188	128	188	WRKY	1	60	62	82.1	2.3E-23	1
	BRADI1G22680.1	177	231	177	232	WRKY	1	59	59	80.4	8.3E-23	2
		342	398	342	399	WRKY	1	59	59	84.4	4.8E-24	
	BRADI1G23340.1	227	283	227	283	WRKY	1	60	60	86.3	1.2E-24	2
		401	458	401	458	WRKY	1	60	60	91.9	2.1E-26	
	BRADI1G23340.2	151	207	151	207	WRKY	1	60	60	86.5	9.9E-25	2
		325	382	325	382	WRKY	1	60	60	92.2	1.7E-26	
	BRADI1G30870.1	186	243	186	244	WRKY	1	59	59	86	1.5E-24	1
	BRADI1G47690.1	119	174	118	174	WRKY	2	60	59	85.9	1.5E-24	2
		273	293	273	294	WRKY	1	21	21	23.4	0.000053	
	BRADI1G48770.1	113	173	113	173	WRKY	1	60	62	90.8	4.5E-26	1
	BRADI1G51030.1	268	343	268	343	WRKY	1	60	77	78.9	2.3E-22	1
	BRADI1G59180.1	123	180	123	180	WRKY	1	60	60	90.7	4.9E-26	1
	BRADI1G63220.1	114	172	113	173	WRKY	2	59	60	86.9	7.5E-25	1
	BRADI1G63910.1	33	93	33	94	WRKY	1	59	61	84.7	3.7E-24	1
	BRADI1G63910.2	11	71	11	72	WRKY	1	59	61	85	3E-24	1
	BRADI2G00280.1	141	198	141	198	WRKY	1	60	60	84.9	3.2E-24	2
		281	338	281	338	WRKY	1	60	60	88.5	2.4E-25	
	BRADI2G05234.1	150	206	150	207	WRKY	1	59	59	85.6	1.9E-24	1
	BRADI2G05500.1	262	320	262	320	WRKY	1	60	60	91.3	3.1E-26	1
	BRADI2G05510.1	149	206	149	206	WRKY	1	60	60	80.4	8.4E-23	1
	BRADI2G08620.1	363	420	362	420	WRKY	2	60	59	93.2	8.4E-27	1
	BRADI2G11170.1	231	288	230	288	WRKY	2	60	59	91.7	2.4E-26	1
	BRADI2G15360.1	161	218	161	218	WRKY	1	60	60	90.8	4.7E-26	1
	BRADI2G15405.1	167	224	167	225	WRKY	1	59	59	78.5	3.2E-22	1
	BRADI2G15877.1	98	157	98	158	WRKY	1	59	61	89.1	1.6E-25	1
	BRADI2G16150.1	326	383	325	383	WRKY	2	60	59	93	9.6E-27	1

BRADI2G16357.1	208	265	208	265	WRKY	1	60	60	92	2E-26	1
BRADI2G18530.1	157	213	157	214	WRKY	1	59	59	86	1.4E-24	1
BRADI2G19070.1	160	217	160	217	WRKY	1	60	60	90.5	5.9E-26	1
BRADI2G22230.1	138	199	138	199	WRKY	1	60	62	76.5	1.3E-21	1
BRADI2G22440.1	184	240	184	240	WRKY	1	60	60	86.9	7.5E-25	2
	347	404	347	404	WRKY	1	60	60	90.1	7.8E-26	
BRADI2G30695.1	108	171	108	171	WRKY	1	60	64	74.2	6.9E-21	1
BRADI2G30800.1	158	217	158	218	WRKY	1	59	61	85.6	2E-24	1
BRADI2G33540.1	95	151	95	152	WRKY	1	59	59	83.6	8.2E-24	1
BRADI2G44035.1	181	238	180	238	WRKY	2	60	59	78.9	2.4E-22	1
BRADI2G44090.1	196	253	196	253	WRKY	1	60	60	88.8	2E-25	1
BRADI2G44270.1	101	161	101	161	WRKY	1	60	62	79.4	1.7E-22	1
BRADI2G45480.1	136	195	136	196	WRKY	1	59	61	83.7	7.6E-24	1
BRADI2G45900.1	184	241	184	241	WRKY	1	60	60	89.9	9.1E-26	1
BRADI2G48090.1	132	188	132	189	WRKY	1	59	59	82.3	2.1E-23	2
	65	121	64	122	WRKY	2	59	58	90.8	4.5E-26	
BRADI2G48907.1	65	121	64	122	WRKY	2	59	58	90.8	4.5E-26	1
BRADI2G49020.1	144	201	144	201	WRKY	1	60	60	90.6	5.5E-26	1
BRADI2G49906.1	86	143	85	143	WRKY	2	60	59	90.3	6.8E-26	1
BRADI2G53480.1	52	72	51	73	WRKY	2	22	21	30.9	2.4E-07	1
BRADI2G53495.1	68	127	67	128	WRKY	2	59	60	78.8	2.6E-22	1
BRADI2G53500.1	124	187	124	187	WRKY	1	60	64	74.5	5.5E-21	1
BRADI2G53510.1	42	106	41	106	WRKY	2	60	65	75.5	2.7E-21	1
BRADI2G53520.1	111	169	111	170	WRKY	1	59	60	81.5	3.8E-23	1
BRADI2G53760.1	229	284	229	285	WRKY	1	59	59	84.7	3.6E-24	2
	398	455	398	455	WRKY	1	60	60	89	1.7E-25	
BRADI2G54720.1	111	147	104	148	WRKY	21	59	39	41.7	9.6E-11	1
BRADI2G62130.1	141	162	141	163	WRKY	1	22	22	30.9	2.4E-07	1
BRADI3G06070.1	186	243	186	244	WRKY	1	59	59	86.1	1.3E-24	1
BRADI3G09810.1	169	225	168	226	WRKY	2	59	58	87.1	6.6E-25	1
BRADI3G18580.1	240	296	239	297	WRKY	2	59	58	90.3	6.5E-26	1
BRADI3G19640.1	259	313	258	314	WRKY	2	59	58	76.5	1.4E-21	2
	430	486	429	486	WRKY	2	60	59	89.4	1.3E-25	
BRADI3G34567.1	183	240	182	240	WRKY	2	60	59	90.4	6.4E-26	1
BRADI3G34850.1	119	179	119	179	WRKY	1	60	62	93.2	8.2E-27	1
BRADI3G39340.1	256	312	256	312	WRKY	1	60	60	87.5	4.9E-25	2
	373	429	373	430	WRKY	1	59	59	91.2	3.6E-26	
BRADI3G50360.1	156	212	156	213	WRKY	1	59	59	85.8	1.7E-24	1
BRADI3G52420.1	227	284	226	284	WRKY	2	60	59	89.2	1.4E-25	1
BRADI3G57710.1	331	389	331	389	WRKY	1	60	60	94.9	2.5E-27	1
BRADI4G01950.1	160	217	160	217	WRKY	1	60	60	86.3	1.2E-24	2
	316	373	316	373	WRKY	1	60	60	91	4E-26	
BRADI4G02680.1	287	343	286	344	WRKY	2	59	58	92	1.9E-26	1
BRADI4G06690.1	230	286	230	286	WRKY	1	60	60	85.1	2.8E-24	1
BRADI4G09890.1	837	893	837	893	WRKY	1	60	60	76.4	1.5E-21	1
BRADI4G19060.1	117	174	117	174	WRKY	1	60	61	94.5	3.2E-27	1
BRADI4G25717.1	51	111	51	112	WRKY	1	59	61	87.3	5.7E-25	1
BRADI4G30360.1	180	238	180	238	WRKY	1	60	60	84.2	5.4E-24	1
BRADI4G30370.1	102	142	102	153	WRKY	1	41	41	56.3	2.8E-15	1

BRADI4G33370.1	286	342	286	342	WRKY	1	60	60	80.1	1E-22	2
	505	562	505	562	WRKY	1	60	60	88.3	2.8E-25	
BRADI4G44342.1	123	192	122	192	WRKY	2	60	70	77.4	7E-22	1
BRADI4G44350.1	131	197	130	198	WRKY	2	59	67	77.5	6.5E-22	1
BRADI4G44360.1	122	188	121	189	WRKY	2	59	67	79.8	1.2E-22	1
BRADI4G44370.1	111	179	110	179	WRKY	2	60	69	85.3	2.5E-24	1
BRADI4G45290.1	118	173	117	173	WRKY	2	60	59	85.7	1.9E-24	2
	275	329	275	330	WRKY	1	59	59	80.5	7.8E-23	
BRADI5G04817.1	237	293	236	294	WRKY	2	59	58	88.6	2.3E-25	1
BRADI5G13090.1	289	345	289	345	WRKY	1	60	60	82.6	1.7E-23	2
	523	580	523	580	WRKY	1	60	60	88.2	3E-25	
BRADI5G17395.1	118	174	118	175	WRKY	1	59	59	88.2	2.9E-25	1
BRADI5G20290.1	140	197	139	197	WRKY	2	60	59	90.5	5.9E-26	1
BRADI5G20700.1	224	280	223	281	WRKY	2	59	58	91.8	2.3E-26	1
***Triticum aestivum* (171 WRKYs)**											
Traes_1AL_0404BC790.1	57	114	57	114	WRKY	1	60	60	93.1	9.00E-27	1
Traes_1AL_180B9CF01.1	63	120	63	120	WRKY	1	60	60	89.7	1.00E-25	1
Traes_1AL_309623B48.1	136	197	136	197	WRKY	1	60	62	73.5	1.20E-20	1
Traes_1AL_4E924201A.1	184	241	183	241	WRKY	2	60	59	90.4	6.10E-26	1
Traes_1AL_9ADA7A031.1	133	189	133	190	WRKY	1	59	59	85.5	2.00E-24	1
Traes_1AL_B24F28600.1	135	195	135	196	WRKY	1	59	61	77.2	8.00E-22	1
Traes_1AL_F64E07A92.1	27	84	27	84	WRKY	1	60	60	91.1	3.60E-26	1
Traes_1AS_1432A2F79.1	36	92	36	93	WRKY	1	59	59	85.9	1.50E-24	1
Traes_1AS_F3EAEC435.1	179	236	179	236	WRKY	1	60	60	86.2	1.30E-24	2
	324	381	324	381	WRKY	1	60	60	88.6	2.30E-25	
Traes_1BL_1D865A8CC.1	9	46	6	46	WRKY	21	60	40	48.7	6.40E-13	1
Traes_1BL_46340D685.1	134	195	134	195	WRKY	1	60	62	73.5	1.20E-20	1
Traes_1BL_73811B853.1	14	73	14	74	WRKY	1	59	61	89.5	1.20E-25	1
Traes_1BL_794E99FF5.1	183	240	182	240	WRKY	2	60	59	90.4	6.10E-26	1
Traes_1BL_9AFA4B870.1	108	167	108	167	WRKY	1	60	62	82.9	1.30E-23	1
Traes_1BL_B15990028.1	39	96	39	96	WRKY	1	60	60	89.6	1.10E-25	1
Traes_1BL_B4AFDB663.1	134	194	134	195	WRKY	1	59	61	79	2.30E-22	1
Traes_1BS_EF67E5A24.1	186	243	186	243	WRKY	1	60	60	86.1	1.40E-24	2
	334	391	334	391	WRKY	1	60	60	88.1	3.30E-25	
Traes_1DL_46428511F.1	43	100	43	100	WRKY	1	60	60	89.5	1.20E-25	1
Traes_1DL_5BAB0B6BC.1	57	114	57	114	WRKY	1	60	60	93.1	9.00E-27	1
Traes_1DL_D1EC7DEA6.1	66	111	66	112	WRKY	1	48	48	71.4	5.40E-20	1
Traes_1DL_D550418641.2	11	67	11	68	WRKY	1	59	59	87.9	3.60E-25	1
Traes_1DL_DFE1721E0.1	141	202	141	202	WRKY	1	60	62	72.9	1.80E-20	1
Traes_1DS_A6733B734.1	184	241	184	241	WRKY	1	60	60	86	1.40E-24	2
	338	395	338	395	WRKY	1	60	60	88.5	2.50E-25	
Traes_2AL_15A7BB684.1	236	292	235	293	WRKY	2	59	58	91.2	3.60E-26	1
Traes_2AL_1B43EA59E.1	69	126	69	126	WRKY	1	60	60	91.5	2.90E-26	1
Traes_2AL_409AB7647.1	294	350	294	350	WRKY	1	60	60	82.6	1.70E-23	2
	523	580	523	580	WRKY	1	60	60	88.2	3.00E-25	
Traes_2AL_434E9F101.1	243	299	242	300	WRKY	2	59	58	91	4.10E-26	1
Traes_2AL_B1270662B.1	152	208	152	209	WRKY	1	59	59	87.8	3.90E-25	1
Traes_2AS_0186B9E4F.2	152	206	152	207	WRKY	1	59	59	75.6	2.60E-21	2
	316	373	316	373	WRKY	1	60	60	87.4	5.30E-25	

Traes_2AS_1AFFE8DA6.1	23	44	22	44	WRKY	2	23	22	32.9	5.40E-08	1
Traes_2AS_6269D889E.1	120	180	120	180	WRKY	1	60	62	94.1	4.30E-27	1
Traes_2AS_C407071E4.2	188	244	188	244	WRKY	1	60	60	84.5	4.20E-24	2
	362	419	362	419	WRKY	1	60	60	92	1.90E-26	
Traes_2AS_D0C21ADB5.1	91	118	91	126	WRKY	1	28	28	38.4	1.10E-09	1
Traes_2BL_2BA3A755A.1	299	355	299	355	WRKY	1	60	60	82.6	1.70E-23	2
	528	585	528	585	WRKY	1	60	60	88.2	3.00E-25	
Traes_2BL_6B75B32E3.1	223	279	222	280	WRKY	2	59	58	91.3	3.30E-26	1
Traes_2BL_A5BFA97B9.1	152	208	152	209	WRKY	1	59	59	87.8	3.90E-25	1
Traes_2BL_A69F6C5DF.1	127	184	127	184	WRKY	1	60	60	90.4	6.10E-26	1
Traes_2BS_380EC4D1E.1	199	253	199	254	WRKY	1	59	59	78	4.60E-22	2
	363	420	363	420	WRKY	1	60	60	87.2	6.10E-25	
Traes_2BS_B65714572.1	119	179	119	179	WRKY	1	60	62	95.4	1.70E-27	1
Traes_2BS_D435A8999.1	141	197	141	197	WRKY	1	60	60	84.7	3.80E-24	2
	314	371	314	371	WRKY	1	60	60	92.2	1.70E-26	
Traes_2BS_F3097F116.1	112	169	112	169	WRKY	1	60	61	94.6	3.00E-27	1
Traes_2DL_04535D371.1	127	184	127	184	WRKY	1	60	60	89.2	1.40E-25	1
Traes_2DL_362A1F535.1	24	69	24	70	WRKY	1	45	47	55.4	5.10E-15	1
Traes_2DL_4F9F8F1F0.1	152	208	152	209	WRKY	1	59	59	87.8	3.90E-25	1
Traes_2DL_F600B5FDF.1	30	86	29	87	WRKY	2	59	58	93.9	4.90E-27	1
Traes_2DS_0F2500A60.1	99	156	99	156	WRKY	1	60	61	94.8	2.70E-27	1
Traes_2DS_97E3E7CFC.1	141	197	141	197	WRKY	1	60	60	84.7	3.80E-24	2
	315	372	315	372	WRKY	1	60	60	92.2	1.70E-26	
Traes_2DS_AD8820C42.1	166	186	166	188	WRKY	1	21	21	27.1	3.50E-06	1
Traes_2DS_F6FBC974C.2	189	243	189	244	WRKY	1	59	59	75.8	2.20E-21	2
	353	373	353	373	WRKY	1	21	21	28.9	1.00E-06	
Traes_3AL_140B829CB.2	194	251	194	251	WRKY	1	60	60	88.8	2.00E-25	1
Traes_3AL_1B73D2C12.1	13	51	9	52	WRKY	19	59	41	44.6	1.20E-11	1
Traes_3AL_2297D6E18.1	68	127	67	128	WRKY	2	59	60	81.6	3.50E-23	1
Traes_3AL_3160E1F30.1	2	57	1	58	WRKY	2	59	58	98.1	2.50E-28	1
Traes_3AL_4769A72F1.1	1	38	1	38	WRKY	21	60	40	49.9	2.80E-13	1
Traes_3AL_67ECA2932.1	18	39	18	45	WRKY	1	22	22	31.5	1.50E-07	1
Traes_3AL_6E92D4E1F.1	9	68	9	69	WRKY	1	59	61	91.5	2.80E-26	1
Traes_3AL_AB2BAE660.1	120	155	120	155	WRKY	1	37	37	53.6	1.90E-14	1
Traes_3AL_DED8A29EC.1	48	68	48	70	WRKY	1	21	21	31.9	1.10E-07	1
Traes_3AL_F326C5B8E.1	41	97	41	98	WRKY	1	59	59	90.4	6.10E-26	1
Traes_3AS_5CD024A9E.1	19	76	18	76	WRKY	2	60	59	99	1.30E-28	1
Traes_3B_8B0D448D8.1	50	109	49	110	WRKY	2	59	60	84.1	5.90E-24	1
Traes_3B_990298FF5.1	217	272	217	273	WRKY	1	59	59	85.7	1.80E-24	2
	381	438	381	438	WRKY	1	60	60	88.4	2.50E-25	
TRAES3BF001300030CFD_t1	105	162	104	163	WRKY	2	59	59	83.7	7.60E-24	1
TRAES3BF003800010CFD_t1	307	364	306	364	WRKY	2	60	59	93.3	7.40E-27	2
	105	162	104	163	WRKY	2	59	59	83.5	8.50E-24	
TRAES3BF005100020CFD_t1	128	147	127	147	WRKY	2	21	20	27.2	3.40E-06	1
TRAES3BF005100030CFD_t1	128	148	127	150	WRKY	2	22	21	27	3.80E-06	2
	157	222	156	222	WRKY	2	60	66	72.4	2.60E-20	
TRAES3BF021100090CFD_t1	195	252	195	252	WRKY	1	60	60	89.7	1.00E-25	1
TRAES3BF021300010CFD_t1	195	252	195	252	WRKY	1	60	60	89.7	1.00E-25	1
TRAES3BF029000080CFD_t1	80	137	79	137	WRKY	2	60	59	88.7	2.00E-25	2

	177	233	177	234	WRKY	1	59	59	86.5	1.00E-24	
TRAES3BF051200110CFD_t1	123	182	123	183	WRKY	1	59	61	85.6	2.00E-24	1
TRAES3BF058500060CFD_t1	167	224	167	224	WRKY	1	60	60	89.9	8.70E-26	1
TRAES3BF066700160CFD_t1	129	185	129	186	WRKY	1	59	59	78.6	3.00E-22	1
TRAES3BF073300120CFD_t1	75	131	74	132	WRKY	2	59	58	88.2	2.90E-25	1
TRAES3BF081400030CFD_t1	28	83	27	84	WRKY	2	59	58	68.2	5.20E-19	1
TRAES3BF090100100CFD_t1	151	206	150	207	WRKY	2	59	58	91.7	2.40E-26	1
TRAES3BF111700140CFD_t1	116	172	116	173	WRKY	1	59	59	83.6	8.20E-24	1
TRAES3BF180700010CFD_t1	163	220	162	220	WRKY	2	60	59	77.1	8.70E-22	1
TRAES3BF267200010CFD_t1	119	175	119	176	WRKY	1	59	59	83.2	1.10E-23	1
Traes_3DL_2551BF2C1.1	20	52	19	52	WRKY	2	32	33	43.4	2.80E-11	1
Traes_3DL_48F7A19D2.1	68	127	67	128	WRKY	2	59	60	84.6	4.00E-24	1
Traes_3DL_678D51EAD.1	31	68	31	68	WRKY	1	36	38	55.4	5.40E-15	1
Traes_3DL_7456F61A3.1	12	52	11	53	WRKY	2	40	41	66	2.60E-18	1
Traes_3DL_DF0D3F3FE.1	6	62	6	63	WRKY	1	59	59	84.1	5.80E-24	1
Traes_3DS_9A02CF31D.1	219	278	219	279	WRKY	1	59	61	88.2	3.10E-25	1
Traes_3DS_F6B1E6078.1	24	62	24	63	WRKY	1	40	40	64.9	5.60E-18	1
Traes_4AL_234E1CDF6.1	65	120	65	121	WRKY	1	59	59	72.6	2.20E-20	1
Traes_4AL_2EEECCC4B.1	94	150	94	150	WRKY	1	60	60	80.4	8.00E-23	2
	261	318	261	318	WRKY	1	60	60	87.8	4.00E-25	
Traes_4AL_3E11167D9.1	225	262	225	262	WRKY	1	38	38	59.1	3.80E-16	1
Traes_4AL_98B1C762B.1	283	339	282	340	WRKY	2	59	58	94.8	2.50E-27	1
Traes_4AL_9E0D1CFA6.1	42	102	42	103	WRKY	1	59	61	85.8	1.70E-24	1
Traes_4AL_C2A825B6D.1	43	81	43	81	WRKY	1	40	40	60.4	1.40E-16	1
Traes_4AS_0DA136E0E.1	5	56	3	56	WRKY	10	60	53	58	8.30E-16	1
Traes_4AS_70DF607CC.1	33	93	33	94	WRKY	1	59	61	84.7	3.60E-24	1
Traes_4BL_A8C6FBEB6.1	33	93	33	94	WRKY	1	59	61	84.7	3.60E-24	1
Traes_4BL_EFEC50B26.2	88	144	88	144	WRKY	1	60	60	80.9	5.70E-23	2
	255	278	255	283	WRKY	1	24	24	34.5	1.70E-08	
Traes_4BS_A6D9EB0E5.1	98	158	98	159	WRKY	1	59	61	85.3	2.50E-24	1
Traes_4BS_CE839571B.2	273	326	272	329	WRKY	2	56	55	83.8	7.30E-24	1
Traes_4DL_3140A8240.1	33	93	33	94	WRKY	1	59	61	84.7	3.60E-24	
Traes_4DS_3BE557D5C.2	274	326	273	327	WRKY	2	55	54	81.5	3.80E-23	1
Traes_4DS_CFC487CE5.2	53	108	53	109	WRKY	1	59	59	72.7	2.10E-20	1
Traes_4DS_DC3C9DC42.2	203	259	203	259	WRKY	1	60	60	79.9	1.20E-22	2
	370	427	370	427	WRKY	1	60	60	87.3	5.90E-25	
Traes_4DS_FE38A59D0.1	42	102	42	103	WRKY	1	59	61	85.8	1.70E-24	1
Traes_5AL_06A6F9328.2	31	88	31	88	WRKY	1	60	60	91.6	2.60E-26	1
Traes_5AL_69A969FF4.1	34	106	33	106	WRKY	2	60	73	82.1	2.30E-23	1
Traes_5AL_6F7D1D441.1	53	109	52	110	WRKY	2	59	58	80.6	7.00E-23	1
Traes_5AL_6FDB440FB.1	4	50	2	50	WRKY	20	60	47	43	3.90E-11	1
Traes_5AL_7164FEAC3.1	46	83	46	83	WRKY	1	38	38	56	3.50E-15	1
Traes_5AL_A3653B781.1	52	108	51	109	WRKY	2	59	58	95.4	1.70E-27	1
Traes_5AL_B4E8A3115.2	1333	1392	1333	1393	WRKY	1	59	61	87	7.20E-25	1
Traes_5AL_E566BD64E.1	1	48	1	48	WRKY	11	60	50	61.2	8.10E-17	1
Traes_5AL_E644A6A0B.1	117	172	116	172	WRKY	2	60	59	85.8	1.70E-24	2
	277	329	275	330	WRKY	3	59	57	78.1	4.40E-22	
Traes_5AL_ED3ADED51.3	278	334	278	334	WRKY	1	60	60	87.8	4.10E-25	2
	491	548	491	548	WRKY	1	60	60	88.6	2.30E-25	

Traes_5AS_433D3E526.1	70	126	69	127	WRKY	2	59	58	93.6	6.40E-27	1
Traes_5AS_9C6171380.1	92	148	92	148	WRKY	1	60	60	85.8	1.70E-24	2
	249	306	249	306	WRKY	1	60	60	91.6	2.50E-26	
Traes_5BL_0A3D332A8.1	122	181	122	182	WRKY	1	59	61	93.8	5.30E-27	1
Traes_5BL_175E7FC38.1	133	197	132	197	WRKY	2	60	65	79.4	1.70E-22	1
Traes_5BL_17A712C94.1	278	334	278	334	WRKY	1	60	60	87.5	5.00E-25	2
	491	548	491	548	WRKY	1	60	60	88.3	2.70E-25	
Traes_5BL_8688F70C9.1	234	290	233	291	WRKY	2	59	58	93.7	5.70E-27	1
Traes_5BL_8BEF7F9CD.1	172	230	172	230	WRKY	1	60	60	86.5	1.00E-24	1
Traes_5BL_90757F0CC.1	173	231	173	231	WRKY	1	60	60	86.5	1.00E-24	1
Traes_5BL_A522C62D1.1	117	172	116	172	WRKY	2	60	59	85.8	1.70E-24	2
	277	329	275	330	WRKY	3	59	57	78.1	4.40E-22	
Traes_5BL_AEF9FE805.2	333	389	332	390	WRKY	2	59	58	92.2	1.70E-26	1
Traes_5BL_B9DD3E76F.1	193	250	192	250	WRKY	2	60	59	88.7	2.10E-25	1
Traes_5BL_C1D6B6B74.2	143	200	143	200	WRKY	1	60	60	90.4	6.10E-26	1
Traes_5BL_D3C383CF5.1	191	247	191	247	WRKY	1	60	60	76.4	1.50E-21	2
	315	337	315	339	WRKY	1	23	23	34	2.50E-08	
Traes_5BL_E294922A9.2	308	364	307	365	WRKY	2	59	58	86.6	9.60E-25	1
Traes_5BL_F853EA802.1	125	184	125	185	WRKY	1	59	61	92.2	1.70E-26	1
Traes_5BS_C46781248.1	87	143	87	143	WRKY	1	60	60	85.8	1.70E-24	2
	244	301	244	301	WRKY	1	60	60	91.7	2.50E-26	
Traes_5BS_E0345D5DF.2	289	345	288	346	WRKY	2	59	58	90	8.30E-26	1
Traes_5DL_09F1F8F79.1	130	194	129	194	WRKY	2	60	65	77.7	5.80E-22	1
Traes_5DL_1733FB4DA.1	802	861	802	862	WRKY	1	59	61	76.9	1.00E-21	2
	917	976	917	977	WRKY	1	59	61	88	3.40E-25	
Traes_5DL_21F7C6BF7.2	278	334	278	334	WRKY	1	60	60	87.8	4.00E-25	2
	491	548	491	548	WRKY	1	60	60	88.6	2.20E-25	
Traes_5DL_2553A6C33.1	14	70	13	71	WRKY	2	59	58	89.8	9.40E-26	1
Traes_5DL_32D78D06A.1	47	105	47	105	WRKY	1	60	60	88.3	2.80E-25	1
Traes_5DL_46E3AC8D6.1	187	243	187	243	WRKY	1	60	60	76.4	1.50E-21	2
	314	340	314	342	WRKY	1	27	27	39.1	6.40E-10	
Traes_5DL_4BA2CC560.2	117	172	116	172	WRKY	2	60	59	85.8	1.70E-24	2
	278	330	276	331	WRKY	3	59	57	78.1	4.40E-22	
Traes_5DL_5C93510D5.1	125	184	125	185	WRKY	1	59	61	89.7	9.90E-26	1
Traes_5DL_7E2053226.2	37	94	37	94	WRKY	1	60	60	91.4	3.00E-26	1
Traes_5DL_A54ED44C9.2	327	383	326	384	WRKY	2	59	58	92.2	1.70E-26	1
Traes_5DL_C93641E43.1	121	180	121	181	WRKY	1	59	61	93.8	5.40E-27	1
Traes_5DL_E4A6D1889.2	188	245	187	245	WRKY	2	60	59	88.7	2.10E-25	1
Traes_5DS_5DEA5C9E3.1	229	285	229	285	WRKY	1	60	60	85.1	2.80E-24	2
	386	443	386	443	WRKY	1	60	60	91	4.10E-26	
Traes_5DS_D83DEA9B0.1	290	346	289	347	WRKY	2	59	58	91	3.90E-26	1
Traes_6AL_0C0899C15.1	317	375	317	375	WRKY	1	60	60	93.1	8.60E-27	1
Traes_6AL_A5FB7CFA5.1	230	287	229	287	WRKY	2	60	59	90.9	4.40E-26	1
Traes_6AL_BA4636569.1	162	219	162	219	WRKY	1	60	60	87.9	3.80E-25	1
Traes_6AS_68775100B.1	9	68	9	69	WRKY	1	59	61	90.2	7.30E-26	1
Traes_6AS_DA75BB1FD.1	193	250	193	251	WRKY	1	59	59	88.7	2.10E-25	1
Traes_6BL_B92FA1D38.1	2	58	2	58	WRKY	1	58	58	95.2	1.90E-27	1
Traes_6BL_DD840863A.1	161	218	161	218	WRKY	1	60	60	87.9	3.80E-25	1
Traes_6BL_EEAA2A7E3.1	42	99	41	99	WRKY	**2**	60	59	93.8	5.50E-27	1

Traes_6DL_AB95B0CE0.1	315	373	315	373	WRKY	1	60	60	93.1	8.70E-27	1
Traes_6DL_D29E210A1.1	162	219	162	219	WRKY	1	60	60	87.9	3.80E-25	1
Traes_6DL_D4F2CDDDC.1	230	287	229	287	WRKY	2	60	59	90.9	4.40E-26	1
Traes_6DS_8F684013D.2	77	134	77	135	WRKY	1	59	59	91.1	3.90E-26	1
Traes_6DS_BF71C1557.2	91	125	83	126	WRKY	25	59	36	24.4	2.60E-05	1
Traes_7AL_48C81DE03.1	201	255	200	256	WRKY	2	59	58	77.4	7.10E-22	2
	369	389	368	394	WRKY	2	22	21	28.4	1.40E-06	
Traes_7AL_48C81DE031.1	201	255	200	256	WRKY	2	59	58	77.4	7.10E-22	2
	369	389	368	394	WRKY	2	22	21	28.4	1.40E-06	
Traes_7AL_AC56CC184.1	51	91	51	91	WRKY	1	41	41	61.7	5.50E-17	1
Traes_7AS_C9DF68E53.2	243	301	243	301	WRKY	1	60	60	91.6	2.50E-26	1
Traes_7BL_53AA25AA1.1	5	54	5	55	WRKY	1	49	51	64.4	7.90E-18	1
Traes_7BL_A46F1A830.2	101	155	100	156	WRKY	2	59	58	78.3	3.60E-22	2
	269	290	268	297	WRKY	2	23	22	32	1.10E-07	
Traes_7DL_5968FA56C.1	62	119	62	120	WRKY	1	59	59	84.6	4.00E-24	1
Traes_7DL_A9EF00572.1	112	171	112	172	WRKY	1	59	61	75.9	2.10E-21	1
Traes_7DL_B09854286.1	52	109	52	110	WRKY	1	59	59	86.8	8.00E-25	1
Traes_7DL_B86B36F67.1	23	80	23	81	WRKY	1	59	59	86.4	1.10E-24	1
Traes_7DL_F849918EA.2	62	119	62	120	WRKY	1	59	59	83.6	8.10E-24	1
Traes_7DS_01F74C6F3.1	29	88	29	89	WRKY	1	59	61	93.3	7.50E-27	1
Traes_7DS_24C563960.1	101	155	100	156	WRKY	2	59	58	78.7	2.90E-22	2
	269	290	268	297	WRKY	2	23	22	32	1.10E-07	
***Oryza sativa* (88 WRKYs)**											
OS01T0182700-01	204	260	204	261	WRKY	1	59	59	87.5	4.90E-25	1
OS01T0185900-01	238	296	238	296	WRKY	1	60	60	91.9	2.10E-26	1
OS01T0186000-01	81	137	81	138	WRKY	1	59	59	82.8	1.40E-23	1
OS01T0289600-00	137	194	137	194	WRKY	1	60	60	85.4	2.30E-24	1
OS01T0584900-01	125	182	124	182	WRKY	2	60	59	91.4	3.10E-26	1
OS01T0586800-00	33	90	32	90	WRKY	2	60	59	87.3	5.60E-25	1
OS01T0624700-01	178	235	177	235	WRKY	2	60	59	80	1.10E-22	1
OS01T0626400-01	205	262	205	262	WRKY	1	60	60	88.6	2.30E-25	1
OS01T0656400-00	101	160	101	161	WRKY	1	59	61	88.6	2.20E-25	1
OS01T0714800-01	160	216	160	217	WRKY	1	59	59	82.9	1.30E-23	1
OS01T0730700-01	95	151	94	152	WRKY	2	59	58	91.1	3.60E-26	1
OS01T0734000-01	176	233	176	233	WRKY	1	60	60	90.9	4.30E-26	1
OS01T0750100-01	99	156	98	156	WRKY	2	60	59	91.6	2.60E-26	1
OS01T0750100-02	99	151	98	152	WRKY	2	55	54	80.5	7.40E-23	1
OS01T0750100-03	99	156	98	156	WRKY	2	60	59	91.3	3.20E-26	1
OS01T0820400-00	71	130	70	131	WRKY	2	59	60	83.9	6.60E-24	1
OS01T0820700-00	16	75	15	76	WRKY	2	59	60	87.4	5.30E-25	1
OS01T0821600-01	114	174	113	175	WRKY	2	59	61	79.4	1.60E-22	1
OS01T0826400-01	220	275	220	276	WRKY	1	59	59	88.8	2.00E-25	2
	385	442	385	442	WRKY	1	60	60	89.8	9.30E-26	
OS01T0842801-00	166	186	165	190	WRKY	2	22	21	33.1	4.90E-08	1
OS01T0972800-01	160	216	160	217	WRKY	1	59	59	87.4	5.40E-25	1
OS02T0265200-01	176	232	175	233	WRKY	2	59	58	90.8	4.50E-26	1
OS02T0462800-01	169	225	168	226	WRKY	2	59	58	86.4	1.10E-24	1
OS02T0652100-01	240	297	239	297	WRKY	2	60	59	89.6	1.10E-25	1
OS02T0698800-01	138	159	138	163	WRKY	1	22	22	31.7	1.30E-07	1

OS02T0770500-00	415	473	415	473	WRKY	1	60	60	92.7	1.20E-26	1
OS03T0321700-01	137	193	137	193	WRKY	1	60	60	86	1.50E-24	1
OS03T0335200-01	304	325	304	328	WRKY	1	22	22	32	1.10E-07	1
OS03T0444900-00	33	93	33	94	WRKY	1	59	61	84	6.30E-24	2
	92	152	92	152	WRKY	1	60	62	84.2	5.30E-24	
OS03T0657400-00	322	378	321	379	WRKY	2	59	58	93.2	8.50E-27	1
OS03T0741400-01	48	105	48	106	WRKY	1	59	59	71.8	4.00E-20	1
OS03T0758000-01	52	109	52	109	WRKY	1	60	60	91.6	2.60E-26	1
OS03T0758950-00	200	257	199	257	WRKY	2	60	59	89.6	1.10E-25	2
	194	250	194	250	WRKY	1	60	60	78	4.50E-22	
OS03T0855100-01	340	397	340	397	WRKY	1	60	60	85.3	2.40E-24	1
OS04T0287400-01	523	579	523	579	WRKY	1	60	60	82	2.50E-23	1
OS04T0471700-00	755	812	755	812	WRKY	1	60	60	87.1	6.40E-25	2
	248	304	247	305	WRKY	2	59	58	90.9	4.40E-26	
OS04T0545000-01	164	221	163	221	WRKY	2	60	59	90	8.50E-26	1
OS04T0597300-01	170	226	170	227	WRKY	1	59	59	87.4	5.30E-25	1
OS04T0605100-01	223	279	222	280	WRKY	2	59	58	92.3	1.60E-26	1
OS05T0129800-01	40	60	39	63	WRKY	2	22	21	27.9	2.00E-06	2
	123	164	119	164	WRKY	18	60	43	44.8	1.10E-11	
OS05T0137500-00	222	279	221	279	WRKY	2	60	59	92.3	1.60E-26	1
OS05T0183100-01	114	170	114	171	WRKY	1	59	59	81.6	3.40E-23	1
OS05T0321900-01	228	287	228	288	WRKY	1	59	61	86.6	9.30E-25	1
OS05T0322900-01	118	178	118	178	WRKY	1	60	62	77.8	5.50E-22	1
OS05T0322900-02	118	138	118	138	WRKY	1	21	21	25.7	1.00E-05	1
OS05T0343400-01	190	247	190	247	WRKY	1	60	60	88.4	2.60E-25	2
	352	409	352	409	WRKY	1	60	60	89.3	1.40E-25	
OS05T0474800-01	154	213	154	214	WRKY	1	59	61	90.8	4.50E-26	2
	219	275	219	275	WRKY	1	60	60	86.8	8.00E-25	
OS05T0478400-00	392	449	392	449	WRKY	1	60	60	90.6	5.40E-26	1
OS05T0478700-00	136	196	136	197	WRKY	1	59	61	70.7	8.80E-20	1
OS05T0478800-00	21	86	21	86	WRKY	1	60	66	84.4	4.60E-24	1
OS05T0537100-00	135	191	135	192	WRKY	1	59	59	87.6	4.50E-25	1
OS05T0565900-00	201	258	201	258	WRKY	1	60	60	91.4	3.10E-26	1
OS05T0571200-01	106	165	106	166	WRKY	1	59	61	87.6	4.60E-25	1
OS05T0583000-01	188	245	188	245	WRKY	1	60	60	93.8	5.20E-27	1
OS06T0146250-00	256	314	256	314	WRKY	1	60	60	94.7	2.90E-27	1
OS06T0158100-00	144	204	144	204	WRKY	1	60	62	93.1	8.80E-27	1
OS06T0504900-00	217	272	216	274	WRKY	2	58	57	77.1	8.80E-22	1
OS06T0649000-01	220	277	220	278	WRKY	1	59	59	87.5	4.80E-25	1
OS06T0649000-02	194	251	194	252	WRKY	1	59	59	87.7	4.30E-25	1
OS07T0111400-00	124	181	124	181	WRKY	1	60	60	93.7	5.80E-27	1
OS07T0583700-01	236	292	236	292	WRKY	1	60	60	84.8	3.50E-24	2
	410	467	410	467	WRKY	1	60	60	91.7	2.50E-26	
OS07T0596900-00	170	226	170	226	WRKY	1	60	60	79.9	1.20E-22	2
	339	395	339	396	WRKY	1	59	59	87.8	4.20E-25	
OS07T0680400-01	66	121	65	121	WRKY	2	60	59	55.4	5.20E-15	1
OS08T0198100-00	280	336	279	337	WRKY	2	59	58	90.6	5.40E-26	1
OS08T0235800-00	229	284	228	284	WRKY	2	60	59	81	5.50E-23	1
OS08T0276200-01	277	332	277	332	WRKY	1	60	60	82.1	2.40E-23	1

OS08T0386200-01	488	545	488	545	WRKY	1	60	60	88.4	2.70E-25	1
OS08T0499300-01	130	190	130	190	WRKY	1	60	62	92.8	1.10E-26	2
	126	185	126	186	WRKY	1	59	61	91.4	3.00E-26	
OS08T0499300-02	166	224	166	224	WRKY	1	60	60	85	2.90E-24	2
	1	41	1	41	WRKY	16	60	45	46.1	4.10E-12	
OS09T0334500-01	197	254	197	254	WRKY	1	60	60	89.5	1.20E-25	1
OS09T0417600-01	166	224	166	224	WRKY	1	60	60	85	2.90E-24	1
OS09T0417600-02	434	491	434	491	WRKY	1	60	60	88.5	2.50E-25	1
OS09T0417800-01	144	201	144	202	WRKY	1	59	59	82.6	1.70E-23	1
OS09T0481700-01	434	491	434	491	WRKY	1	60	60	88.5	2.50E-25	1
OS09T0481700-02	113	177	113	177	WRKY	1	60	65	77.2	8.20E-22	1
OS11T0116900-01	146	214	145	215	WRKY	2	59	69	79.3	1.80E-22	1
OS11T0117400-00	44	104	44	105	WRKY	1	59	61	87.6	4.50E-25	1
OS11T0117500-01	131	150	130	152	WRKY	2	21	20	28.4	1.40E-06	1
OS11T0117600-00	137	194	137	194	WRKY	1	60	61	95.1	2.10E-27	1
OS11T0490900-01	3	31	1	32	WRKY	30	59	30	30.7	2.70E-07	1
OS11T0685700-00	69	129	69	129	WRKY	1	60	62	77.9	5.00E-22	2
	12	54	9	55	WRKY	14	59	46	50.9	1.30E-13	
OS11T0686250-00	42	102	42	103	WRKY	1	59	61	87.7	4.40E-25	1
OS12T0116400-00	73	137	73	137	WRKY	1	60	65	77.6	6.10E-22	1
OS12T0116600-01	147	215	146	216	WRKY	2	59	69	79	2.30E-22	1
OS12T0116700-01	129	185	129	185	WRKY	1	60	60	86.4	1.10E-24	1
OS12T0507300-00	286	343	286	343	WRKY	1	60	60	91.5	2.70E-26	2
	297	353	296	354	WRKY	2	59	58	93.9	5.00E-27	
OS12T0597700-01	122	182	122	183	WRKY	1	59	62	80.8	6.30E-23	1
OS12T0597700-02	197	253	196	254	WRKY	2	59	58	94.6	3.00E-27	1
***Zea mays* (163 WRKYs)**											
AC165171.2_FGP002	129	185	129	186	WRKY	1	59	59	87.7	4.50E-25	1
AC193630.3_FGP003	248	285	248	300	WRKY	1	38	38	61.7	5.60E-17	1
AC194362.3_FGP003	117	136	116	146	WRKY	2	21	20	29.6	5.90E-07	1
AC198725.4_FGP009	198	255	198	255	WRKY	1	60	60	88.7	2.00E-25	1
AC205562.3_FGP002	116	176	116	176	WRKY	1	60	62	91.1	3.60E-26	1
AC208110.2_FGP001	238	259	237	263	WRKY	2	23	22	25.2	1.40E-05	2
	301	338	298	338	WRKY	22	60	39	47.9	1.20E-12	
AC209050.3_FGP003	331	388	331	389	WRKY	1	59	59	86.4	1.10E-24	1
GRMZM2G003551_P01	141	201	141	201	WRKY	1	60	62	88.5	2.50E-25	1
GRMZM2G004060_P01	168	228	168	228	WRKY	1	60	62	77.6	6.10E-22	1
GRMZM2G005207_P01	112	174	111	175	WRKY	2	59	63	81.4	4.00E-23	1
GRMZM2G006497_P01	150	207	149	207	WRKY	2	60	59	83.7	7.90E-24	1
GRMZM2G008029_P01	220	276	220	276	WRKY	1	60	60	79.2	2.00E-22	2
	350	407	350	407	WRKY	1	60	60	84.3	5.00E-24	
GRMZM2G012724_P01	204	261	204	261	WRKY	1	60	60	86	1.50E-24	2
	366	423	366	423	WRKY	1	60	60	88.6	2.30E-25	
GRMZM2G012724_P02	202	259	202	259	WRKY	1	60	60	86	1.50E-24	2
	364	421	364	421	WRKY	1	60	60	88.6	2.30E-25	
GRMZM2G012724_P03	131	188	131	188	WRKY	1	60	60	90	8.20E-26	1
GRMZM2G012724_P04	204	261	204	261	WRKY	1	60	60	86.8	8.40E-25	1
GRMZM2G013391_P01	82	139	81	139	WRKY	2	60	59	89.9	9.00E-26	1
GRMZM2G015433_P01	143	200	143	200	WRKY	1	60	60	90.2	7.30E-26	1

GRMZM2G018487_P01	333	389	332	390	WRKY	2	59	58	93.1	8.80E-27	1
GRMZM2G018487_P02	333	389	332	390	WRKY	2	59	58	93.1	8.80E-27	1
GRMZM2G018721_P01	132	189	132	189	WRKY	1	60	60	90.8	4.70E-26	1
GRMZM2G020254_P01	132	186	131	187	WRKY	2	59	58	87.4	5.30E-25	1
GRMZM2G020254_P02	112	166	111	167	WRKY	2	59	58	87.7	4.50E-25	1
GRMZM2G020254_P03	160	214	159	215	WRKY	2	59	58	87.1	6.60E-25	1
GRMZM2G024898_P01	244	301	243	301	WRKY	2	60	59	90	8.10E-26	1
GRMZM2G025895_P01	139	198	139	199	WRKY	1	59	61	88.2	3.00E-25	1
GRMZM2G027972_P01	277	333	277	333	WRKY	1	60	60	85.5	2.10E-24	2
	537	576	533	576	WRKY	19	60	42	47.9	1.10E-12	
GRMZM2G029282_P01	118	180	118	180	WRKY	1	60	63	76.2	1.70E-21	1
GRMZM2G029292_P02	66	108	60	109	WRKY	19	59	43	47.5	1.50E-12	1
GRMZM2G029292_P03	54	113	53	114	WRKY	2	59	60	87.3	5.70E-25	1
GRMZM2G030272_P01	30	90	30	91	WRKY	1	59	61	86.5	1.00E-24	1
GRMZM2G031963_P01	282	338	282	338	WRKY	1	60	60	82.7	1.50E-23	2
	498	555	498	555	WRKY	1	60	60	87.5	5.00E-25	
GRMZM2G034421_P01	134	195	134	195	WRKY	1	60	62	70	1.40E-19	1
GRMZM2G036703_P01	246	301	246	302	WRKY	1	59	59	87.6	4.70E-25	2
	412	469	412	469	WRKY	1	60	60	87.1	6.40E-25	
GRMZM2G038158_P01	173	229	172	230	WRKY	2	59	58	85	2.90E-24	1
GRMZM2G040298_P01	78	134	77	135	WRKY	2	59	58	87.4	5.20E-25	1
GRMZM2G048450_P01	179	235	178	236	WRKY	2	59	58	89.2	1.50E-25	1
GRMZM2G052671_P01	374	431	374	431	WRKY	1	60	60	87.8	3.90E-25	1
GRMZM2G052671_P02	374	431	374	431	WRKY	1	60	60	87.8	3.90E-25	1
GRMZM2G054125_P01	138	195	138	195	WRKY	1	60	61	94.5	3.30E-27	1
GRMZM2G057011_P01	192	250	192	250	WRKY	1	60	60	85.6	2.00E-24	1
GRMZM2G057116_P01	108	164	108	165	WRKY	1	59	59	85.8	1.70E-24	1
GRMZM2G057116_P02	36	73	14	74	WRKY	20	59	40	46	4.50E-12	1
GRMZM2G059562_P01	109	167	108	167	WRKY	2	60	60	83.3	1.00E-23	1
GRMZM2G060918_P01	30	83	30	87	WRKY	1	52	54	75.2	3.50E-21	1
GRMZM2G061408_P01	139	198	139	199	WRKY	1	59	61	88.2	3.00E-25	1
GRMZM2G063216_P01	44	104	44	105	WRKY	1	59	61	87.4	5.40E-25	1
GRMZM2G063216_P02	2	30	1	31	WRKY	31	59	29	32.1	1.00E-07	1
GRMZM2G063216_P03	44	66	44	71	WRKY	1	23	23	29.1	8.80E-07	1
GRMZM2G063880_P01	96	155	96	156	WRKY	1	59	61	83.7	7.80E-24	1
GRMZM2G065290_P01	72	131	71	132	WRKY	2	59	60	77.8	5.20E-22	1
GRMZM2G070211_P01	287	343	286	344	WRKY	2	59	58	92.3	1.50E-26	1
GRMZM2G071907_P01	213	269	212	270	WRKY	2	59	58	92.6	1.30E-26	1
GRMZM2G071907_P03	200	256	199	257	WRKY	2	59	58	92.7	1.20E-26	1
GRMZM2G073272_P01	249	305	248	306	WRKY	2	59	58	87.6	4.60E-25	1
GRMZM2G076657_P01	228	284	228	284	WRKY	1	60	60	86	1.50E-24	2
	385	441	385	442	WRKY	1	59	59	87.6	4.70E-25	
GRMZM2G076657_P02	228	284	228	284	WRKY	1	60	60	86.1	1.40E-24	2
	385	441	385	442	WRKY	1	59	59	87.6	4.50E-25	
GRMZM2G083350_P01	280	337	279	337	WRKY	2	60	59	90.6	5.20E-26	1
GRMZM2G083717_P01	192	249	191	249	WRKY	2	60	59	90	8.40E-26	1
GRMZM2G090594_P01	235	291	234	292	WRKY	2	59	58	93.1	9.00E-27	1
GRMZM2G091331_P01	226	282	225	283	WRKY	2	59	58	92.2	1.70E-26	1
GRMZM2G091331_P02	226	245	225	245	WRKY	2	21	20	27.6	2.50E-06	1

GRMZM2G092694_P01	266	301	265	304	WRKY	2	37	36	55.9	3.60E-15	1
GRMZM2G099593_P01	131	194	131	195	WRKY	1	59	64	81.9	2.70E-23	1
GRMZM2G101405_P01	129	185	129	186	WRKY	1	59	59	84.3	5.10E-24	1
GRMZM2G102583_P02	253	309	252	310	WRKY	2	59	58	91.9	2.00E-26	1
GRMZM2G105140_P01	176	233	175	233	WRKY	2	60	59	79.1	2.10E-22	1
GRMZM2G106560_P01	129	186	129	186	WRKY	1	60	61	94.4	3.50E-27	1
GRMZM2G106560_P02	2	42	1	42	WRKY	18	60	44	55.4	5.30E-15	1
GRMZM2G111354_P01	149	206	149	206	WRKY	1	60	60	91.3	3.30E-26	1
GRMZM2G111711_P01	201	258	201	259	WRKY	1	59	59	86.3	1.20E-24	1
GRMZM2G120320_P01	234	291	234	292	WRKY	1	59	59	87.3	5.90E-25	1
GRMZM2G123387_P01	205	261	205	262	WRKY	1	59	59	86.4	1.10E-24	1
GRMZM2G123387_P02	24	80	24	81	WRKY	1	59	59	89.4	1.30E-25	1
GRMZM2G125653_P01	154	212	154	212	WRKY	1	60	60	87	7.00E-25	1
GRMZM2G125653_P02	67	125	67	125	WRKY	1	60	60	87.8	3.90E-25	1
GRMZM2G127064_P01	218	277	218	278	WRKY	1	59	61	83.7	7.50E-24	1
GRMZM2G130374_P01	307	363	306	364	WRKY	2	59	58	90.7	4.80E-26	1
GRMZM2G130854_P01	229	284	229	285	WRKY	1	59	59	83	1.30E-23	2
	402	459	402	459	WRKY	1	60	60	91.7	2.40E-26	
GRMZM2G137802_P01	105	161	105	162	WRKY	1	59	59	86.5	1.00E-24	1
GRMZM2G138683_P01	213	270	212	270	WRKY	2	60	59	90.2	7.00E-26	1
GRMZM2G139815_P01	109	168	109	169	WRKY	1	59	61	93	9.60E-27	1
GRMZM2G141299_P01	90	147	89	147	WRKY	2	60	59	90.4	6.20E-26	1
GRMZM2G141299_P02	45	102	44	102	WRKY	2	60	59	90.8	4.70E-26	1
GRMZM2G141299_P03	3	41	1	41	WRKY	21	60	40	49.9	2.70E-13	1
GRMZM2G143204_P01	139	195	139	196	WRKY	1	59	59	92.1	1.80E-26	1
GRMZM2G143204_P02	139	191	139	192	WRKY	1	59	59	69.9	1.50E-19	1
GRMZM2G143204_P03	139	195	139	196	WRKY	1	59	59	92.1	1.80E-26	1
GRMZM2G143765_P01	230	286	230	286	WRKY	1	60	60	86	1.50E-24	2
	387	444	387	444	WRKY	1	60	60	89.4	1.30E-25	
GRMZM2G145554_P01	207	264	207	264	WRKY	1	60	60	87.7	4.40E-25	1
GRMZM2G147880_P01	331	387	330	388	WRKY	2	59	58	93.1	8.80E-27	1
GRMZM2G148087_P01	217	272	217	273	WRKY	1	59	59	87.8	3.90E-25	2
	384	441	384	441	WRKY	1	60	60	88.4	2.50E-25	
GRMZM2G148561_P01	242	298	241	299	WRKY	2	59	58	92.4	1.50E-26	1
GRMZM2G149219_P01	3	60	3	61	WRKY	1	59	59	72.3	2.80E-20	1
GRMZM2G149683_P01	173	230	172	230	WRKY	2	60	59	78.2	3.90E-22	1
GRMZM2G151407_P01	134	191	134	191	WRKY	1	60	60	89.8	9.80E-26	1
GRMZM2G151407_P02	387	444	387	444	WRKY	1	60	60	88.6	2.20E-25	1
GRMZM2G151444_P01	216	272	216	273	WRKY	1	59	59	87.4	5.20E-25	1
GRMZM2G151763_P01	201	258	201	258	WRKY	1	60	60	94.4	3.40E-27	1
GRMZM2G156529_P01	134	191	133	191	WRKY	2	60	59	85.1	2.90E-24	1
GRMZM2G158328_P01	122	184	121	184	WRKY	2	60	63	77.4	7.00E-22	1
GRMZM2G161411_P01	48	105	48	106	WRKY	1	59	59	73.4	1.30E-20	1
GRMZM2G163054_P01	102	125	102	148	WRKY	1	24	24	28.2	1.60E-06	1
GRMZM2G163054_P02	101	157	101	158	WRKY	1	59	59	85	3.00E-24	1
GRMZM2G163054_P03	144	166	144	189	WRKY	1	23	23	27.7	2.40E-06	1
GRMZM2G163054_P04	102	158	102	159	WRKY	1	59	59	85	3.00E-24	1
GRMZM2G163418_P01	134	193	134	194	WRKY	1	59	61	90.7	4.90E-26	1
GRMZM2G163418_P02	134	154	134	157	WRKY	1	21	21	30	4.50E-07	1

GRMZM2G164082_P01	348	404	347	405	WRKY	2	59	58	89.9	9.00E-26	1
GRMZM2G169149_P01	103	160	102	160	WRKY	2	60	59	84.5	4.20E-24	1
GRMZM2G169564_P01	166	235	165	236	WRKY	2	59	70	78.1	4.30E-22	1
GRMZM2G169966_P01	51	107	51	107	WRKY	1	60	60	89.5	1.20E-25	2
	229	286	229	286	WRKY	1	60	60	81.7	3.30E-23	
GRMZM2G171428_P01	212	268	212	268	WRKY	1	60	60	83.9	6.70E-24	2
	381	438	381	438	WRKY	1	60	60	89.5	1.20E-25	
GRMZM2G171428_P02	212	268	212	268	WRKY	1	60	60	84.3	5.10E-24	2
	381	402	381	404	WRKY	1	22	22	30.6	2.90E-07	
GRMZM2G173680_P01	301	357	300	358	WRKY	2	59	58	92.3	1.60E-26	1
GRMZM2G173680_P02	301	357	300	358	WRKY	2	59	58	92.3	1.60E-26	1
GRMZM2G176489_P01	289	346	288	346	WRKY	2	60	59	83.3	1.00E-23	1
GRMZM2G304573_P01	134	192	134	192	WRKY	1	60	60	83.6	8.00E-24	1
GRMZM2G324999_P01	130	190	130	190	WRKY	1	60	62	94.4	3.40E-27	1
GRMZM2G327349_P01	294	351	293	351	WRKY	2	60	59	88.8	1.90E-25	1
GRMZM2G354384_P01	3	60	2	60	WRKY	2	60	60	41.5	1.10E-10	1
GRMZM2G366795_P01	313	370	312	370	WRKY	2	60	59	93.2	8.50E-27	1
GRMZM2G366795_P02	311	368	310	368	WRKY	2	60	59	93.2	8.50E-27	1
GRMZM2G377217_P01	160	216	160	217	WRKY	1	59	59	85.5	2.00E-24	1
GRMZM2G377217_P02	160	181	160	183	WRKY	1	22	22	31.2	1.90E-07	1
GRMZM2G381378_P01	132	193	132	193	WRKY	1	60	62	81	5.30E-23	1
GRMZM2G382350_P01	53	112	52	113	WRKY	2	59	60	86.8	8.20E-25	1
GRMZM2G383594_P01	210	267	209	267	WRKY	2	60	59	87.7	4.30E-25	1
GRMZM2G391892_P01	2	30	1	31	WRKY	31	59	29	29.5	6.30E-07	1
GRMZM2G398506_P01	174	230	174	230	WRKY	1	60	60	85.4	2.30E-24	2
	348	405	348	405	WRKY	1	60	60	92.7	1.20E-26	
GRMZM2G398506_P02	230	286	230	286	WRKY	1	60	60	85.2	2.60E-24	2
	404	461	404	461	WRKY	1	60	60	92.5	1.30E-26	
GRMZM2G400559_P01	162	234	161	235	WRKY	2	59	73	78	4.70E-22	1
GRMZM2G401521_P01	212	269	212	269	WRKY	1	60	60	91.3	3.20E-26	1
GRMZM2G401521_P02	212	234	212	246	WRKY	1	23	23	30.8	2.60E-07	1
GRMZM2G408462_P01	130	189	130	190	WRKY	1	59	61	86.1	1.40E-24	1
GRMZM2G411766_P01	126	185	126	186	WRKY	1	59	61	86.2	1.20E-24	1
GRMZM2G414315_P01	181	237	180	238	WRKY	2	59	58	73.9	9.10E-21	1
GRMZM2G425430_P01	118	174	118	174	WRKY	1	60	60	82.9	1.40E-23	2
	287	302	287	303	WRKY	1	16	16	21.1	0.00026	
GRMZM2G432583_P01	125	185	125	186	WRKY	1	59	62	82	2.70E-23	1
GRMZM2G441031_P01	124	187	123	187	WRKY	2	60	64	71.8	3.90E-20	1
GRMZM2G448605_P01	359	416	358	416	WRKY	2	60	59	89.7	1.00E-25	1
GRMZM2G449681_P01	200	257	200	257	WRKY	1	60	60	86.1	1.30E-24	2
	365	422	365	422	WRKY	1	60	60	90.7	5.10E-26	
GRMZM2G449681_P02	8	65	8	65	WRKY	1	60	60	93.7	5.60E-27	1
GRMZM2G449681_P03	8	65	8	65	WRKY	1	60	60	93.7	5.60E-27	1
GRMZM2G451035_P01	132	151	131	153	WRKY	2	21	20	23.4	5.20E-05	2
	169	215	158	216	WRKY	19	59	47	38.8	7.80E-10	
GRMZM2G453571_P01	187	244	187	244	WRKY	1	60	60	91.4	3.00E-26	1
GRMZM2G461648_P01	132	198	132	199	WRKY	1	59	68	78.9	2.40E-22	1
GRMZM2G468056_P01	98	138	94	138	WRKY	20	60	42	43.7	2.40E-11	1
GRMZM2G475984_P01	145	201	145	202	WRKY	1	59	59	84.1	5.90E-24	1

GRMZM2G501775_P01	270	316	261	317	WRKY	18	59	47	37.9	1.50E-09	1
GRMZM2G516301_P01	121	177	121	178	WRKY	1	59	59	83.8	7.30E-24	1
GRMZM2G549512_P01	251	306	250	306	WRKY	2	60	59	79.2	1.90E-22	2
	418	474	417	474	WRKY	2	60	59	89.2	1.50E-25	
GRMZM5G812272_P01	5	43	3	43	WRKY	20	60	41	50.2	2.20E-13	1
GRMZM5G812272_P02	199	256	199	256	WRKY	1	60	60	93.9	5.10E-27	1
GRMZM5G816457_P01	283	339	283	339	WRKY	1	60	60	82.2	2.20E-23	2
	491	548	491	548	WRKY	1	60	60	86	1.50E-24	
GRMZM5G816457_P02	283	339	283	339	WRKY	1	60	60	82.2	2.20E-23	2
	491	548	491	548	WRKY	1	60	60	86	1.50E-24	
GRMZM5G823157_P01	223	280	222	280	WRKY	2	60	59	90.2	7.40E-26	1
GRMZM5G863420_P01	135	191	135	192	WRKY	1	59	59	86.4	1.10E-24	1
GRMZM5G871347_P01	153	210	152	210	WRKY	2	60	59	96.8	6.10E-28	1
GRMZM5G871347_P02	277	334	276	334	WRKY	2	60	59	96	1.10E-27	1
GRMZM5G871347_P03	320	377	319	377	WRKY	2	60	59	95.8	1.20E-27	1
GRMZM5G880069_P02	299	355	298	356	WRKY	2	59	58	92.3	1.60E-26	1
GRMZM5G880069_P03	299	355	298	356	WRKY	2	59	58	92.3	1.60E-26	1
***Sorghum bicolor* (96 WRKYs)**											
Sb01g000696.1	198	255	197	255	WRKY	2	60	59	89.9	8.60E-26	1
Sb01g005070.1	222	278	221	279	WRKY	2	59	58	90.6	5.20E-26	1
Sb01g007480.1	194	250	194	250	WRKY	1	60	60	79	2.20E-22	2
	318	375	318	375	WRKY	1	60	60	79.5	1.60E-22	
Sb01g007570.1	147	204	147	204	WRKY	1	60	60	89.6	1.10E-25	1
Sb01g008550.1	342	398	341	399	WRKY	2	59	58	93.1	9.10E-27	1
Sb01g012870.1	3	60	3	61	WRKY	1	59	60	73.8	9.40E-21	1
Sb01g014180.1	286	342	285	343	WRKY	2	59	58	93.2	8.30E-27	1
Sb01g027770.1	194	251	193	251	WRKY	2	60	59	89.2	1.40E-25	1
Sb01g032120.1	215	271	215	271	WRKY	1	60	60	83.4	9.60E-24	2
	384	441	384	441	WRKY	1	60	60	89	1.80E-25	
Sb01g036180.1	110	170	110	170	WRKY	1	60	62	85.5	2.10E-24	1
Sb01g036870.1	30	90	30	91	WRKY	1	59	61	87.5	4.80E-25	1
Sb02g000960.1	175	213	169	213	WRKY	20	60	41	37	3.00E-09	1
Sb02g008880.1	809	868	809	869	WRKY	1	59	61	52.2	5.20E-14	1
Sb02g011050.1	16	73	15	73	WRKY	2	60	59	85.2	2.60E-24	1
Sb02g021226.1	1354	1412	1354	1412	WRKY	1	60	60	78.5	3.20E-22	2
	1487	1548	1487	1548	WRKY	1	60	62	69.7	1.80E-19	
Sb02g022280.1	107	166	107	167	WRKY	1	59	61	90.4	6.30E-26	1
Sb02g022290.1	142	201	142	202	WRKY	1	59	61	91.5	2.80E-26	1
Sb02g024760.1	167	225	167	225	WRKY	1	60	60	86.4	1.10E-24	1
Sb02g024765.1	88	146	88	146	WRKY	1	60	60	86.4	1.10E-24	1
Sb02g027950.1	405	462	405	462	WRKY	1	60	60	88.5	2.30E-25	1
Sb02g037660.1	230	286	230	286	WRKY	1	60	60	85.5	2.10E-24	2
	404	461	404	461	WRKY	1	60	60	92.6	1.30E-26	
Sb02g043030.1	126	186	126	186	WRKY	1	60	62	80.9	5.60E-23	1
Sb03g000240.1	286	343	285	343	WRKY	2	60	59	95.4	1.70E-27	1
Sb03g003360.1	138	194	138	195	WRKY	1	59	59	84.8	3.40E-24	1
Sb03g003370.1	288	345	287	345	WRKY	2	60	59	89	1.60E-25	1
Sb03g003640.1	218	274	218	275	WRKY	1	59	59	87.4	5.30E-25	1
Sb03g011800.1	293	350	292	350	WRKY	2	60	59	88.8	1.90E-25	1

Sb03g026170.1	138	195	138	195	WRKY	1	60	60	86.2	1.30E-24	1
Sb03g026280.1	150	208	150	208	WRKY	1	60	60	87.6	4.50E-25	1
Sb03g028440.1	245	302	244	302	WRKY	2	60	59	78.7	2.80E-22	1
Sb03g028530.1	213	270	213	270	WRKY	1	60	60	88.7	2.10E-25	1
Sb03g029920.1	144	203	144	204	WRKY	1	59	61	86.9	7.70E-25	1
Sb03g030480.1	209	266	209	266	WRKY	1	60	60	91.9	2.10E-26	1
Sb03g032800.1	139	195	139	196	WRKY	1	59	59	82.5	1.90E-23	1
Sb03g033640.1	95	151	94	152	WRKY	2	59	58	87.1	6.50E-25	1
Sb03g033780.1	168	225	168	225	WRKY	1	60	60	91.1	3.90E-26	1
Sb03g034670.1	99	156	98	156	WRKY	2	60	59	89.7	1.00E-25	1
Sb03g038170.1	53	112	52	113	WRKY	2	59	60	87.3	5.80E-25	1
Sb03g038180.1	68	127	67	128	WRKY	2	59	60	76.3	1.60E-21	1
Sb03g038190.1	120	186	120	186	WRKY	1	60	67	76.8	1.10E-21	1
Sb03g038200.1	124	184	123	184	WRKY	2	60	61	78	4.50E-22	1
Sb03g038210.1	116	174	115	174	WRKY	2	60	60	83.1	1.20E-23	1
Sb03g038510.1	248	303	248	304	WRKY	1	59	59	87.7	4.20E-25	2
	409	466	409	466	WRKY	1	60	60	89.7	1.00E-25	
Sb03g039550.1	163	218	162	219	WRKY	2	59	58	89	1.60E-25	1
Sb03g047350.1	141	197	141	198	WRKY	1	59	59	85.4	2.30E-24	1
Sb04g005520.1	194	251	194	252	WRKY	1	59	59	87.7	4.30E-25	1
Sb04g009800.1	171	227	170	228	WRKY	2	59	58	88.8	2.00E-25	1
Sb04g016540.1	164	220	163	221	WRKY	2	59	58	88.6	2.20E-25	1
Sb04g030930.1	233	290	232	290	WRKY	2	60	59	90.1	7.70E-26	1
Sb04g033240.1	159	215	159	216	WRKY	1	59	59	85.5	2.00E-24	1
Sb04g034440.1	319	377	319	377	WRKY	1	60	60	92.9	1.00E-26	1
Sb05g001170.1	132	193	132	194	WRKY	1	59	62	83	1.30E-23	1
Sb05g001175.1	25	69	19	70	WRKY	17	59	45	49.8	2.90E-13	1
Sb05g001180.1	117	171	116	172	WRKY	2	59	61	43.7	2.40E-11	1
Sb05g001200.1	118	167	104	167	WRKY	17	60	50	46	4.40E-12	1
Sb05g001210.1	98	118	97	119	WRKY	2	22	21	29.5	6.60E-07	1
Sb05g001220.1	121	191	120	191	WRKY	2	60	71	80	1.10E-22	1
Sb05g017130.1	134	191	134	191	WRKY	1	60	61	94.3	3.60E-27	1
Sb06g013835.1	146	205	146	206	WRKY	1	59	61	88.6	2.20E-25	1
Sb06g019710.1	278	334	278	334	WRKY	1	60	60	82.5	1.80E-23	2
	502	559	502	559	WRKY	1	60	60	87.7	4.40E-25	
Sb06g024220.1	169	225	169	226	WRKY	1	59	59	88	3.50E-25	1
Sb06g027290.1	278	335	277	335	WRKY	2	60	59	89.5	1.20E-25	1
Sb06g027710.1	232	288	231	289	WRKY	2	59	58	92.2	1.60E-26	1
Sb07g005740.1	61	117	61	117	WRKY	1	60	60	42.7	4.70E-11	1
Sb07g006230.1	367	421	366	422	WRKY	2	59	58	84.4	4.50E-24	1
Sb07g006980.1	250	306	249	307	WRKY	2	59	58	94.1	4.40E-27	1
Sb07g016330.1	254	309	253	309	WRKY	2	60	59	79.2	1.90E-22	2
	421	477	420	477	WRKY	2	60	59	89.2	1.50E-25	
Sb07g019400.1	131	191	131	191	WRKY	1	60	62	94.1	4.40E-27	1
Sb07g028430.1	89	145	89	145	WRKY	1	60	60	83	1.20E-23	2
	299	356	299	356	WRKY	1	60	60	89	1.70E-25	
Sb08g002520.1	118	182	117	183	WRKY	2	59	65	81.2	4.70E-23	1
Sb08g002540.1	104	152	94	152	WRKY	18	60	49	39.6	4.60E-10	1
Sb08g002560.1	150	214	149	214	WRKY	2	60	65	77.9	5.00E-22	1

	Sb08g002570.1	96	160	95	160	WRKY	2	60	65	73.3	1.30E-20	1
	Sb08g002590.1	42	98	42	99	WRKY	1	59	59	73.1	1.50E-20	1
	Sb08g005080.1	111	171	111	171	WRKY	1	60	62	78.3	3.70E-22	1
	Sb08g016240.1	231	287	231	287	WRKY	1	60	60	85.1	2.80E-24	2
		388	445	388	445	WRKY	1	60	60	91	4.10E-26	
	Sb08g020270.1	305	361	304	362	WRKY	2	59	58	94	4.60E-27	1
	Sb08g021870.1	1010	1050	1002	1051	WRKY	21	59	41	25.9	8.80E-06	1
	Sb09g005700.1	110	166	110	167	WRKY	1	59	59	85.8	1.70E-24	1
	Sb09g010210.1	191	212	191	214	WRKY	1	22	22	29.8	5.10E-07	1
	Sb09g015900.1	218	275	218	275	WRKY	1	60	60	86.1	1.30E-24	2
		387	444	387	444	WRKY	1	60	60	89.2	1.50E-25	
	Sb09g015900.2	217	274	217	274	WRKY	1	60	60	86.1	1.30E-24	2
		386	443	386	443	WRKY	1	60	60	89.2	1.50E-25	
	Sb09g023270.1	240	296	240	296	WRKY	1	60	60	88.3	2.80E-25	2
		416	473	416	473	WRKY	1	60	60	86.7	8.90E-25	
	Sb09g023500.1	128	189	128	189	WRKY	1	60	62	68.1	5.90E-19	1
	Sb09g026350.1	155	212	155	212	WRKY	1	60	60	91.1	3.60E-26	1
	Sb09g026830.1	133	189	133	190	WRKY	1	59	59	87.5	4.90E-25	1
	Sb09g028660.1	194	251	194	251	WRKY	1	60	60	91.3	3.10E-26	1
	Sb09g028750.1	329	387	329	387	WRKY	1	60	60	91.9	2.10E-26	1
	Sb09g029050.1	111	170	111	171	WRKY	1	59	61	83.4	9.30E-24	1
	Sb09g029810.1	195	252	195	252	WRKY	1	60	60	91.5	2.90E-26	1
	Sb09g029850.1	162	218	161	219	WRKY	2	59	58	74.5	5.50E-21	1
	Sb10g004000.1	131	191	131	191	WRKY	1	60	62	88.8	1.90E-25	1
	Sb10g019923.1	85	141	84	141	WRKY	2	60	59	75.2	3.60E-21	1
	Sb10g020010.1	149	206	148	206	WRKY	2	60	59	83.1	1.20E-23	1
	Sb10g025590.1	191	248	191	249	WRKY	1	59	59	88.5	2.30E-25	1
	Sb10g025600.1	189	246	189	247	WRKY	1	59	59	88.6	2.30E-25	1
**Eudicots**	***Populus_trichocarpa*（122 WRKYs）**											
	POPTR_0001s00350.1	244	300	243	301	WRKY	2	59	58	89.4	1.30E-25	1
	POPTR_0001s03620.1	164	222	164	222	WRKY	1	60	60	85.6	2.00E-24	1
	POPTR_0001s03970.1	36	90	36	91	WRKY	1	59	59	70.5	1.00E-19	1
	POPTR_0001s09900.1	158	214	157	215	WRKY	2	59	58	88.3	2.80E-25	1
	POPTR_0001s10490.1	131	190	131	191	WRKY	1	59	61	92.7	1.20E-26	1
	POPTR_0001s13600.1	84	104	84	104	WRKY	1	21	21	32.1	1.00E-07	1
	POPTR_0001s13610.1	28	66	20	66	WRKY	20	60	41	52.1	5.60E-14	1
	POPTR_0001s21580.1	263	317	263	317	WRKY	1	60	60	75.5	2.70E-21	1
	POPTR_0001s33560.1	107	164	107	164	WRKY	1	60	60	93.2	8.40E-27	1
	POPTR_0001s34520.1	170	227	170	227	WRKY	1	60	60	92.9	1.00E-26	1
	POPTR_0001s37260.1	213	269	213	269	WRKY	1	60	60	85.9	1.60E-24	2
		378	435	378	435	WRKY	1	60	60	91.3	3.30E-26	
	POPTR_0001s46490.1	209	266	208	266	WRKY	2	60	59	89.9	8.70E-26	1
	POPTR_0001s47670.1	313	367	311	367	WRKY	3	60	58	73.1	1.60E-20	2
		523	580	523	580	WRKY	1	60	60	87.1	6.60E-25	
	POPTR_0001s47670.2	313	367	311	367	WRKY	3	60	58	73.1	1.50E-20	2
		523	580	523	580	WRKY	1	60	60	85.2	2.60E-24	
	POPTR_0002s04440.1	285	342	284	342	WRKY	2	60	59	95.9	1.20E-27	1
	POPTR_0002s06000.1	182	239	182	239	WRKY	1	60	60	90.9	4.40E-26	1
	POPTR_0002s12480.1	286	342	285	343	WRKY	2	59	58	92.4	1.50E-26	1

POPTR_0002s13980.1	130	186	130	187	WRKY	1	59	59	88.5	2.50E-25	1
POPTR_0002s16590.1	225	282	224	282	WRKY	2	60	59	88.3	2.80E-25	1
POPTR_0002s16590.2	197	254	196	254	WRKY	2	60	59	88.5	2.40E-25	1
POPTR_0002s16640.1	47	104	47	104	WRKY	1	60	60	90.7	4.80E-26	1
POPTR_0002s17010.1	129	189	129	189	WRKY	1	60	62	91	4.00E-26	1
POPTR_0002s18770.1	267	324	266	324	WRKY	2	60	59	91.8	2.30E-26	1
POPTR_0002s19390.1	159	216	159	216	WRKY	1	60	60	93	9.20E-27	1
POPTR_0002s19630.1	53	110	52	110	WRKY	2	60	59	87.9	3.60E-25	1
POPTR_0002s21330.1	340	393	339	393	WRKY	2	60	59	72	3.40E-20	1
POPTR_0002s22180.1	104	160	104	160	WRKY	1	60	60	80.3	8.70E-23	2
	219	275	219	276	WRKY	1	59	59	90.2	7.40E-26	
POPTR_0003s11120.1	240	296	239	297	WRKY	2	59	58	93.5	6.40E-27	1
POPTR_0003s13250.1	154	210	153	211	WRKY	2	59	58	84	5.90E-24	1
POPTR_0003s13840.1	131	190	131	191	WRKY	1	59	61	94.5	3.30E-27	1
POPTR_0003s16750.1	81	138	81	138	WRKY	1	60	60	91.5	2.70E-26	1
POPTR_0003s18060.1	165	220	165	220	WRKY	1	60	60	73.1	1.60E-20	1
POPTR_0003s20860.1	25	79	25	80	WRKY	1	59	59	69.4	2.20E-19	1
POPTR_0004s00890.1	318	375	317	375	WRKY	2	60	59	92.3	1.60E-26	1
POPTR_0004s05920.1	76	133	75	133	WRKY	2	60	59	90.2	7.20E-26	1
POPTR_0004s07050.1	195	253	195	253	WRKY	1	60	60	90.2	7.00E-26	1
POPTR_0004s12000.1	209	265	209	265	WRKY	1	60	60	89.8	9.40E-26	2
	369	426	369	426	WRKY	1	60	60	92.1	1.80E-26	
POPTR_0005s05700.1	286	342	285	343	WRKY	2	59	58	92.2	1.80E-26	1
POPTR_0005s08720.1	115	172	115	172	WRKY	1	60	60	81	5.40E-23	1
POPTR_0005s08860.1	145	164	145	164	WRKY	1	20	20	26.9	4.10E-06	1
POPTR_0005s08870.1	18	56	9	57	WRKY	19	59	41	54.2	1.30E-14	1
POPTR_0005s18490.1	260	316	259	317	WRKY	2	59	58	92.9	9.90E-27	1
POPTR_0005s22480.1	184	241	184	241	WRKY	1	60	60	91.7	2.30E-26	1
POPTR_0005s24100.1	281	338	280	338	WRKY	2	60	59	95.9	1.10E-27	1
POPTR_0006s07170.1	223	279	222	280	WRKY	2	59	58	91.1	3.80E-26	1
POPTR_0006s08730.1	115	171	115	172	WRKY	1	59	59	88.6	2.20E-25	1
POPTR_0006s10600.1	268	323	268	324	WRKY	1	59	59	85	3.00E-24	2
	430	487	430	487	WRKY	1	60	60	88.2	3.00E-25	
POPTR_0006s10950.1	133	193	133	193	WRKY	1	60	62	82.8	1.50E-23	1
POPTR_0006s13550.1	196	250	195	251	WRKY	2	59	58	87.9	3.70E-25	2
	395	451	394	451	WRKY	2	60	59	89.6	1.10E-25	
POPTR_0006s19850.1	96	151	95	151	WRKY	2	60	59	61.6	6.00E-17	2
	282	304	281	307	WRKY	2	24	23	34.9	1.40E-08	
POPTR_0006s24050.1	105	162	105	162	WRKY	1	60	60	87.8	4.00E-25	1
POPTR_0006s27950.1	165	221	164	222	WRKY	2	59	58	83.6	8.30E-24	1
POPTR_0006s27990.1	32	88	31	88	WRKY	2	60	59	74	7.90E-21	2
	120	176	120	177	WRKY	1	59	59	85.3	2.50E-24	
POPTR_0007s06720.1	101	158	101	158	WRKY	1	60	60	78	4.50E-22	1
POPTR_0007s06930.1	157	213	157	214	WRKY	1	59	59	88.8	2.00E-25	1
POPTR_0007s06930.2	157	213	157	214	WRKY	1	59	59	88.8	2.00E-25	1
POPTR_0007s10670.1	264	320	263	321	WRKY	2	59	58	93.4	7.10E-27	1
POPTR_0007s10670.2	263	319	262	320	WRKY	2	59	58	93.4	7.00E-27	1
POPTR_0008s09140.1	222	278	222	278	WRKY	1	60	60	92.3	1.60E-26	2
	391	448	391	448	WRKY	1	60	60	89.1	1.50E-25	

POPTR_0008s09360.1	154	211	154	211	WRKY	1	60	60	90	8.00E-26	1
POPTR_0008s10280.1	216	273	216	273	WRKY	1	60	60	94.4	3.50E-27	1
POPTR_0010s15750.1	223	280	223	280	WRKY	1	60	60	92.3	1.60E-26	1
POPTR_0010s16760.1	154	211	154	211	WRKY	1	60	60	89.8	9.70E-26	1
POPTR_0010s17040.1	223	279	223	279	WRKY	1	60	60	90.2	7.30E-26	2
	394	451	394	451	WRKY	1	60	60	91.3	3.30E-26	
POPTR_0011s02790.1	185	242	185	242	WRKY	1	60	60	91.3	3.20E-26	1
POPTR_0011s06990.1	76	133	75	133	WRKY	2	60	59	89.8	9.30E-26	1
POPTR_0011s09030.1	209	265	209	265	WRKY	1	60	60	82.9	1.30E-23	2
	376	395	376	395	WRKY	1	20	20	25.2	1.40E-05	
POPTR_0011s16050.1	231	288	230	288	WRKY	2	60	59	90.3	6.80E-26	1
POPTR_0011s17230.1	310	366	310	366	WRKY	1	60	60	85.7	1.90E-24	2
	522	579	522	579	WRKY	1	60	60	87.1	6.50E-25	
POPTR_0012s01380.1	134	193	134	194	WRKY	1	59	61	94.2	4.20E-27	1
POPTR_0012s10290.1	100	157	100	157	WRKY	1	60	60	95.9	1.20E-27	1
POPTR_0013s03990.1	287	343	286	344	WRKY	2	59	58	94.6	3.10E-27	1
POPTR_0013s08380.1	156	212	156	212	WRKY	1	60	60	82.4	1.90E-23	2
	278	335	278	335	WRKY	1	60	60	88.7	2.10E-25	
POPTR_0013s09460.1	173	233	173	233	WRKY	1	60	62	87.2	6.40E-25	1
POPTR_0013s09470.1	132	192	132	192	WRKY	1	60	62	79.9	1.20E-22	1
POPTR_0013s10780.1	9	48	6	48	WRKY	20	60	41	51	1.20E-13	1
POPTR_0013s14960.1	258	314	258	314	WRKY	1	60	60	89.2	1.50E-25	2
	431	488	431	488	WRKY	1	60	60	90.1	7.90E-26	
POPTR_0014s00690.1	28	58	23	59	WRKY	28	59	32	27.5	2.80E-06	1
POPTR_0014s02420.1	316	372	315	373	WRKY	2	59	58	92.2	1.60E-26	1
POPTR_0014s04890.1	155	175	155	175	WRKY	1	21	21	29.7	5.70E-07	1
POPTR_0014s04900.1	13	50	5	51	WRKY	20	59	40	50.4	1.90E-13	1
POPTR_0014s08600.1	165	222	164	222	WRKY	2	60	59	88.6	2.20E-25	1
POPTR_0014s08600.2	154	211	153	211	WRKY	2	60	59	88.8	2.00E-25	1
POPTR_0014s08640.1	39	96	39	96	WRKY	1	60	60	92.5	1.40E-26	1
POPTR_0014s09190.1	129	188	129	189	WRKY	1	59	61	93.4	7.20E-27	1
POPTR_0014s10750.1	267	324	266	324	WRKY	2	60	59	92.5	1.30E-26	1
POPTR_0014s11350.1	158	215	158	215	WRKY	1	60	60	93.3	7.90E-27	1
POPTR_0014s11490.1	53	110	52	110	WRKY	2	60	59	87.5	4.80E-25	1
POPTR_0014s15320.1	352	409	351	409	WRKY	2	60	59	93.2	8.30E-27	1
POPTR_0014s16220.1	106	162	106	162	WRKY	1	60	60	79.2	2.00E-22	2
	276	331	275	332	WRKY	2	59	58	90.2	7.20E-26	
POPTR_0014s16220.2	106	162	106	162	WRKY	1	60	60	79.2	2.00E-22	2
	276	331	275	332	WRKY	2	59	58	90.2	7.20E-26	
POPTR_0014s16220.3	106	162	106	162	WRKY	1	60	60	79.2	2.00E-22	2
	276	331	275	332	WRKY	2	59	58	90.2	7.20E-26	
POPTR_0014s16220.4	7	63	7	63	WRKY	1	60	60	79.6	1.40E-22	2
	177	232	176	233	WRKY	2	59	58	90.7	5.10E-26	
POPTR_0014s16220.5	106	162	106	162	WRKY	1	60	60	79.2	2.00E-22	2
	276	331	275	332	WRKY	2	59	58	90.2	7.20E-26	
POPTR_0015s07530.1	209	267	209	267	WRKY	1	60	60	92.8	1.10E-26	1
POPTR_0015s11130.1	100	157	100	157	WRKY	1	60	60	93.8	5.40E-27	1
POPTR_0015s11130.2	100	122	100	125	WRKY	1	23	23	34.7	1.50E-08	1
POPTR_0016s08440.1	187	241	186	242	WRKY	2	59	58	88.4	2.70E-25	2

	302	358	301	358	WRKY	2	60	59	90.7	5.00E-26	
POPTR_0016s08440.2	187	241	186	242	WRKY	2	59	58	88.4	2.70E-25	2
	302	358	301	358	WRKY	2	60	59	90.7	5.00E-26	
POPTR_0016s10610.1	119	174	118	175	WRKY	2	59	58	88.7	2.20E-25	1
POPTR_0016s13600.1	249	305	249	305	WRKY	1	60	60	89.4	1.20E-25	2
	410	467	410	467	WRKY	1	60	60	89.3	1.30E-25	
POPTR_0016s14490.1	130	190	130	190	WRKY	1	60	62	80.8	6.20E-23	1
POPTR_0017s00960.1	222	280	222	280	WRKY	1	60	60	90.6	5.50E-26	1
POPTR_0017s09750.1	108	165	108	165	WRKY	1	60	60	91.6	2.60E-26	1
POPTR_0017s11570.1	208	266	208	266	WRKY	1	60	60	90.9	4.20E-26	1
POPTR_0017s12420.1	1	37	1	37	WRKY	22	60	39	46.6	3.00E-12	1
POPTR_0017s12430.1	240	295	239	295	WRKY	2	60	59	90.2	6.90E-26	2
	412	434	412	446	WRKY	1	23	23	32.9	5.50E-08	
POPTR_0018s02470.1	165	222	165	223	WRKY	1	59	59	88.2	3.00E-25	1
POPTR_0018s02470.2	163	220	163	221	WRKY	1	59	59	88.2	3.00E-25	1
POPTR_0018s02480.1	103	161	103	161	WRKY	1	60	60	82.7	1.50E-23	1
POPTR_0018s02550.1	83	138	82	139	WRKY	2	59	58	73	1.70E-20	1
POPTR_0018s03450.1	256	312	255	313	WRKY	2	59	58	92.6	1.30E-26	1
POPTR_0018s11620.1	1	37	1	37	WRKY	22	60	39	38.3	1.20E-09	1
POPTR_0018s11630.1	216	270	215	271	WRKY	2	59	58	58	8.30E-16	2
	388	410	387	419	WRKY	2	24	23	31.9	1.20E-07	
POPTR_0018s11630.2	209	263	208	264	WRKY	2	59	58	58	8.10E-16	2
	381	403	380	412	WRKY	2	24	23	31.9	1.10E-07	
POPTR_0018s11630.3	212	266	211	267	WRKY	2	59	58	58	8.20E-16	2
	384	406	383	415	WRKY	2	24	23	31.9	1.20E-07	
POPTR_0018s13600.1	222	278	221	279	WRKY	2	59	58	91.5	2.90E-26	1
POPTR_0019s08110.1	264	320	264	320	WRKY	1	60	60	80.7	6.80E-23	2
	431	488	431	488	WRKY	1	60	60	82.3	2.20E-23	
POPTR_0019s08730.1	183	243	183	243	WRKY	1	60	62	89	1.70E-25	1
POPTR_0019s14460.1	258	314	258	314	WRKY	1	60	60	83.7	7.80E-24	2
	433	490	433	490	WRKY	1	60	60	90.1	7.40E-26	
POPTR_0021s00280.1	335	392	334	392	WRKY	2	60	59	91.6	2.60E-26	1
POPTR_0810s00200.1	185	242	185	242	WRKY	1	60	60	91.4	3.00E-26	1
***Cucumis sativu* (88 WRKYs)**											
Cucsa.000200.1	8	66	8	66	WRKY	1	60	60	91.9	2.10E-26	2
	161	218	161	218	WRKY	1	60	60	83.7	7.60E-24	
Cucsa.012940.1	143	202	143	203	WRKY	1	59	61	93.1	9.00E-27	1
Cucsa.041700.1	331	387	331	387	WRKY	1	60	60	87	7.30E-25	2
	546	603	546	603	WRKY	1	60	60	86.8	8.40E-25	
Cucsa.041700.2	331	387	331	387	WRKY	1	60	60	87	7.20E-25	2
	546	603	546	603	WRKY	1	60	60	86.8	8.30E-25	
Cucsa.042320.1	210	266	210	266	WRKY	1	60	60	85.4	2.30E-24	2
	386	443	386	443	WRKY	1	60	60	90	8.50E-26	
Cucsa.042330.2	138	194	138	194	WRKY	1	60	60	85.7	1.90E-24	2
	314	371	314	371	WRKY	1	60	60	90.2	6.90E-26	
Cucsa.042340.3	138	194	138	194	WRKY	1	60	60	85.7	1.90E-24	2
	314	371	314	371	WRKY	1	60	60	90.2	6.90E-26	
Cucsa.042350.4	210	266	210	266	WRKY	1	60	60	86.2	1.20E-24	1
Cucsa.045040.1	353	410	352	410	WRKY	2	60	59	91.9	2.10E-26	1

Cucsa.047510.1	116	172	116	173	WRKY	1	59	59	91	4.10E-26	1
Cucsa.048520.1	261	318	260	318	WRKY	2	60	59	89.5	1.20E-25	1
Cucsa.049620.1	177	235	177	235	WRKY	1	60	60	94.6	3.00E-27	1
Cucsa.054040.1	91	147	91	148	WRKY	1	59	59	92.4	1.50E-26	1
Cucsa.056150.1	21	56	15	56	WRKY	19	56	38	37.9	1.50E-09	1
Cucsa.066300.1	159	215	159	216	WRKY	1	59	59	90.2	7.30E-26	1
Cucsa.073190.1	223	279	222	280	WRKY	2	59	58	95.4	1.70E-27	1
Cucsa.073190.2	222	278	221	279	WRKY	2	59	58	95.4	1.70E-27	1
Cucsa.078810.1	102	160	101	161	WRKY	2	59	60	90.7	5.00E-26	1
Cucsa.087850.1	50	107	50	107	WRKY	1	60	60	90.3	6.50E-26	1
Cucsa.093390.1	195	250	194	250	WRKY	2	60	59	63.8	1.20E-17	2
	369	424	368	425	WRKY	2	59	58	83.8	7.00E-24	
Cucsa.093390.2	194	249	193	249	WRKY	2	60	59	63.8	1.20E-17	2
	368	423	367	424	WRKY	2	59	58	83.8	7.00E-24	
Cucsa.095090.1	136	193	136	193	WRKY	1	60	60	91	3.90E-26	1
Cucsa.095090.2	136	192	136	192	WRKY	1	60	60	80.6	7.00E-23	1
Cucsa.096680.1	54	111	53	111	WRKY	2	60	59	88.7	2.00E-25	1
Cucsa.096680.2	54	107	53	107	WRKY	2	60	59	67.6	8.40E-19	1
Cucsa.096680.3	9	66	8	66	WRKY	2	60	59	89.2	1.50E-25	1
Cucsa.097890.1	182	240	182	240	WRKY	1	60	60	92.5	1.40E-26	1
Cucsa.100440.1	112	168	112	168	WRKY	1	60	60	80.3	8.90E-23	2
	286	342	286	343	WRKY	1	59	59	92.2	1.70E-26	
Cucsa.100440.2	112	168	112	168	WRKY	1	60	60	80.3	8.90E-23	2
	286	342	286	343	WRKY	1	59	59	92.2	1.70E-26	
Cucsa.100440.3	112	168	112	168	WRKY	1	60	60	80.3	8.90E-23	2
	286	342	286	343	WRKY	1	59	59	92.2	1.70E-26	
Cucsa.100440.4	112	168	112	168	WRKY	1	60	60	80.6	7.20E-23	2
	286	342	286	343	WRKY	1	59	59	92.5	1.30E-26	
Cucsa.101460.1	163	219	163	220	WRKY	1	59	59	90.1	7.50E-26	1
Cucsa.101530.1	84	141	84	141	WRKY	1	60	60	84	6.10E-24	1
Cucsa.102600.1	223	279	222	280	WRKY	2	59	58	93.9	4.90E-27	1
Cucsa.106960.1	140	196	139	197	WRKY	2	59	58	91.1	3.80E-26	1
Cucsa.106970.1	122	179	122	179	WRKY	1	60	60	87.5	5.10E-25	1
Cucsa.106970.2	122	175	122	175	WRKY	1	60	60	64.9	5.80E-18	1
Cucsa.107310.1	131	190	131	191	WRKY	1	59	61	90.9	4.20E-26	1
Cucsa.111910.1	233	289	233	289	WRKY	1	60	60	89.2	1.40E-25	2
	408	465	408	465	WRKY	1	60	60	89.8	9.30E-26	
Cucsa.111910.2	233	289	233	289	WRKY	1	60	60	89.8	9.60E-26	2
	408	428	408	428	WRKY	1	21	21	29	8.90E-07	
Cucsa.118200.1	79	135	79	136	WRKY	1	59	59	95.2	2.00E-27	1
Cucsa.143830.1	121	183	121	183	WRKY	1	60	63	80.1	9.90E-23	1
Cucsa.143830.2	120	182	120	182	WRKY	1	60	63	80.2	9.80E-23	1
Cucsa.143840.1	159	219	159	219	WRKY	1	60	62	91	4.10E-26	1
Cucsa.148640.1	197	254	196	254	WRKY	2	60	59	93	9.60E-27	1
Cucsa.158040.1	233	289	233	289	WRKY	1	60	60	90.6	5.50E-26	2
	393	450	393	450	WRKY	1	60	60	89	1.70E-25	
Cucsa.162810.1	146	204	146	204	WRKY	1	60	60	80.3	8.60E-23	1
Cucsa.162820.1	90	146	90	147	WRKY	1	59	59	83.8	6.90E-24	1
Cucsa.167500.1	198	255	197	255	WRKY	2	60	59	89.1	1.60E-25	1

Cucsa.168370.1	118	175	117	175	WRKY	2	60	59	96.7	6.70E-28	1
Cucsa.176780.1	171	226	171	227	WRKY	1	59	59	87.9	3.80E-25	2
	321	377	320	377	WRKY	2	60	59	88.2	3.00E-25	
Cucsa.176780.2	170	225	170	226	WRKY	1	59	59	87.9	3.80E-25	2
	320	376	319	376	WRKY	2	60	59	88.2	2.90E-25	
Cucsa.177060.1	123	182	123	183	WRKY	1	59	61	85.5	2.10E-24	1
Cucsa.177230.1	160	218	160	218	WRKY	1	60	60	85.8	1.70E-24	1
Cucsa.177230.2	109	167	109	167	WRKY	1	60	60	86.2	1.30E-24	1
Cucsa.195730.1	183	240	182	240	WRKY	2	60	59	86.4	1.10E-24	1
Cucsa.197550.1	268	326	268	326	WRKY	1	60	60	91.9	2.20E-26	1
Cucsa.197550.2	239	297	239	297	WRKY	1	60	60	92	2.00E-26	1
Cucsa.201710.1	176	233	175	233	WRKY	2	60	59	91.1	3.80E-26	1
Cucsa.204920.1	150	207	150	207	WRKY	1	60	60	91	4.00E-26	1
Cucsa.212870.1	78	134	77	135	WRKY	2	59	58	85.9	1.60E-24	1
Cucsa.239590.1	157	214	157	214	WRKY	1	60	60	89.5	1.20E-25	1
Cucsa.247040.1	163	220	163	220	WRKY	1	60	60	95.5	1.50E-27	1
Cucsa.250310.1	170	226	170	227	WRKY	1	59	59	85.8	1.70E-24	1
Cucsa.250350.1	128	185	128	185	WRKY	1	60	60	83.4	9.80E-24	1
Cucsa.250350.2	128	148	128	148	WRKY	1	21	21	27.6	2.50E-06	1
Cucsa.252440.1	154	211	154	211	WRKY	1	60	60	89.7	1.00E-25	1
Cucsa.258700.1	160	217	159	217	WRKY	2	60	59	89.9	8.90E-26	1
Cucsa.258730.1	94	151	94	151	WRKY	1	60	60	86.9	7.60E-25	1
Cucsa.259110.1	129	188	129	189	WRKY	1	59	61	92.1	1.80E-26	1
Cucsa.260060.1	146	202	146	203	WRKY	1	59	59	88.1	3.20E-25	1
Cucsa.271570.1	168	225	168	226	WRKY	1	59	59	84.1	5.70E-24	1
Cucsa.282040.1	213	270	212	270	WRKY	2	60	59	92.9	1.00E-26	1
Cucsa.284730.1	199	255	199	255	WRKY	1	60	60	91.4	3.00E-26	2
	379	436	379	436	WRKY	1	60	60	91.6	2.60E-26	
Cucsa.300610.1	157	214	157	214	WRKY	1	60	60	91.7	2.40E-26	1
Cucsa.300610.2	157	214	157	214	WRKY	1	60	60	91.8	2.20E-26	1
Cucsa.300610.3	157	214	157	214	WRKY	1	60	60	92.1	1.90E-26	1
Cucsa.300610.4	69	116	68	116	WRKY	11	60	50	47.5	1.50E-12	1
Cucsa.307270.1	76	131	75	131	WRKY	2	60	59	89	1.70E-25	2
	273	327	272	328	WRKY	3	59	57	80.6	7.10E-23	
Cucsa.307270.2	76	131	75	131	WRKY	2	60	59	89	1.70E-25	2
	273	326	272	327	WRKY	3	59	57	70.2	1.30E-19	
Cucsa.311060.1	272	328	271	329	WRKY	2	59	58	92.2	1.80E-26	1
Cucsa.327760.1	152	209	152	209	WRKY	1	60	60	85.9	1.60E-24	1
Cucsa.352410.1	321	377	320	378	WRKY	2	59	58	91.1	3.70E-26	1
Cucsa.352410.2	320	376	319	377	WRKY	2	59	58	91.1	3.60E-26	1
Cucsa.352410.3	321	340	320	341	WRKY	2	21	20	27.9	2.00E-06	1
Cucsa.356720.1	80	135	80	136	WRKY	1	59	59	87.7	4.30E-25	2
	268	325	268	325	WRKY	1	60	60	86.2	1.30E-24	
Cucsa.388650.1	286	343	285	343	WRKY	2	60	59	94.6	2.90E-27	1
Cucsa.388650.2	189	246	188	246	WRKY	2	60	59	95.4	1.70E-27	1
***Arabidopsis thaliana* (91 WRKYs)**											
AT1G13960.1	229	285	229	285	WRKY	1	60	60	91.9	2.10E-26	2
	409	466	409	466	WRKY	1	60	60	90.4	6.10E-26	
AT1G13960.2	202	258	202	258	WRKY	1	60	60	92	2.00E-26	2

	382	439	382	439	WRKY	1	60	60	90.5	5.70E-26	
AT1G18860.1	191	249	191	249	WRKY	1	60	60	90.6	5.40E-26	1
AT1G29280.1	75	132	74	132	WRKY	2	60	59	92.7	1.20E-26	1
AT1G29860.1	136	193	136	193	WRKY	1	60	60	93	9.50E-27	1
AT1G30650.1	218	275	217	275	WRKY	2	60	59	90.1	7.50E-26	1
AT1G55600.1	307	364	307	364	WRKY	1	60	60	80.9	5.80E-23	1
AT1G62300.1	313	370	312	370	WRKY	2	60	59	94	4.70E-27	1
AT1G64000.1	114	171	114	171	WRKY	1	60	60	91.5	2.80E-26	1
AT1G66550.1	108	168	108	168	WRKY	1	60	62	78.6	2.90E-22	1
AT1G66550.2	103	163	103	163	WRKY	1	60	62	78.7	2.80E-22	1
AT1G66560.1	103	163	103	163	WRKY	1	60	62	81.4	3.90E-23	1
AT1G66600.1	103	163	103	163	WRKY	1	60	62	79.6	1.40E-22	1
AT1G68150.1	235	293	235	293	WRKY	1	60	60	95.7	1.40E-27	1
AT1G69310.1	147	204	147	204	WRKY	1	60	60	91.7	2.50E-26	1
AT1G69310.2	147	204	147	204	WRKY	1	60	60	91.7	2.50E-26	1
AT1G69810.1	203	262	203	262	WRKY	1	60	60	87.1	6.70E-25	1
AT1G80590.1	86	145	85	145	WRKY	2	60	61	77	9.60E-22	1
AT1G80840.1	146	204	146	204	WRKY	1	60	60	81.5	3.80E-23	1
AT2G03340.1	250	306	250	306	WRKY	1	60	60	90.5	5.80E-26	2
	415	472	415	472	WRKY	1	60	60	89	1.70E-25	
AT2G04880.1	111	167	111	167	WRKY	1	60	60	83.6	8.50E-24	2
	307	363	307	364	WRKY	1	59	59	91.2	3.40E-26	
AT2G04880.2	111	167	111	167	WRKY	1	60	60	83.7	7.90E-24	2
	283	339	283	340	WRKY	1	59	59	91.3	3.20E-26	
AT2G21900.1	109	166	109	166	WRKY	1	60	60	78.1	4.40E-22	1
AT2G23320.1	241	297	240	298	WRKY	2	59	58	94.2	3.90E-27	1
AT2G23320.2	241	260	240	262	WRKY	2	21	20	28.9	1.00E-06	1
AT2G24570.1	244	301	243	301	WRKY	2	60	59	91.9	2.20E-26	1
AT2G25000.1	146	203	146	204	WRKY	1	59	59	83.6	8.30E-24	1
AT2G30250.1	166	222	166	222	WRKY	1	60	60	78.5	3.20E-22	2
	329	385	328	385	WRKY	2	60	59	87.7	4.40E-25	
AT2G30590.1	314	371	313	371	WRKY	2	60	59	92	2.00E-26	1
AT2G34830.1	216	273	215	273	WRKY	2	60	59	89.9	8.80E-26	1
AT2G37260.1	166	220	165	221	WRKY	2	59	58	86.3	1.20E-24	2
	349	406	349	406	WRKY	1	60	60	90.2	7.00E-26	
AT2G37260.2	84	138	83	139	WRKY	2	59	58	86.7	8.70E-25	2
	267	324	267	324	WRKY	1	60	60	90.7	5.10E-26	
AT2G38470.1	184	240	184	240	WRKY	1	60	60	87.8	3.90E-25	2
	362	419	362	419	WRKY	1	60	60	88.8	2.00E-25	
AT2G40740.1	173	232	173	233	WRKY	1	59	61	87.9	3.80E-25	1
AT2G40740.2	146	188	139	189	WRKY	18	59	44	50.5	1.80E-13	1
AT2G40750.1	152	211	152	212	WRKY	1	59	61	85.7	1.80E-24	1
AT2G44745.1	145	201	145	202	WRKY	1	59	59	88.1	3.20E-25	1
AT2G46130.1	30	87	30	87	WRKY	1	60	60	91.3	3.30E-26	1
AT2G46130.2	18	71	18	71	WRKY	1	60	60	70.1	1.30E-19	1
AT2G46400.1	104	163	104	164	WRKY	1	59	61	80.7	6.50E-23	1
AT2G47260.1	174	230	174	231	WRKY	1	59	59	90.1	7.70E-26	1
AT3G01080.1	167	222	167	223	WRKY	1	59	59	86.1	1.30E-24	2
	306	363	306	363	WRKY	1	60	60	89.2	1.50E-25	

AT3G01970.1	65	121	65	122	WRKY	1	59	59	88.5	2.40E-25	1
AT3G04670.1	263	319	262	320	WRKY	2	59	58	92.5	1.40E-26	1
AT3G04670.2	263	286	262	288	WRKY	2	25	24	34	2.60E-08	1
AT3G32090.1	90	116	82	116	WRKY	33	60	28	21.4	0.00021	1
AT3G56400.1	120	179	120	180	WRKY	1	59	61	85.4	2.30E-24	1
AT3G58710.1	72	129	71	129	WRKY	2	60	59	86.4	1.10E-24	1
AT3G58710.2	71	128	70	128	WRKY	2	60	59	86.4	1.10E-24	1
AT3G62340.1	118	175	118	175	WRKY	1	60	60	86.7	9.10E-25	1
AT4G01250.1	129	186	128	186	WRKY	2	60	59	90.2	7.00E-26	1
AT4G01720.1	239	297	239	297	WRKY	1	60	60	92.3	1.50E-26	1
AT4G04450.1	293	350	292	350	WRKY	2	60	59	92.2	1.70E-26	1
AT4G11070.1	141	200	141	201	WRKY	1	59	61	89.7	1.10E-25	1
AT4G11070.2	109	168	109	169	WRKY	1	59	61	89.9	8.80E-26	1
AT4G12020.1	468	524	468	524	WRKY	1	60	60	75.7	2.40E-21	2
	641	661	641	663	WRKY	1	21	21	28.7	1.10E-06	
AT4G12020.2	468	524	468	524	WRKY	1	60	60	75.6	2.60E-21	2
	641	661	641	663	WRKY	1	21	21	28.6	1.20E-06	
AT4G12020.3	468	524	468	524	WRKY	1	60	60	75.6	2.60E-21	2
	641	661	641	663	WRKY	1	21	21	28.6	1.20E-06	
AT4G18170.1	172	229	172	229	WRKY	1	60	60	92	2.00E-26	1
AT4G22070.1	298	355	297	355	WRKY	2	60	59	93.4	7.30E-27	1
AT4G23550.1	135	192	134	192	WRKY	2	60	59	89.2	1.50E-25	1
AT4G23810.1	158	217	158	218	WRKY	1	59	61	89.6	1.10E-25	1
AT4G24240.1	282	338	281	339	WRKY	2	59	58	90.5	5.80E-26	1
AT4G26440.1	178	233	178	234	WRKY	1	59	59	84.8	3.50E-24	2
	372	428	372	429	WRKY	1	59	59	84.6	3.90E-24	
AT4G26640.1	139	195	139	195	WRKY	1	60	60	85.6	2.00E-24	2
	309	365	309	366	WRKY	1	59	59	88.5	2.40E-25	
AT4G26640.2	211	267	211	267	WRKY	1	60	60	85.3	2.40E-24	2
	381	437	381	438	WRKY	1	59	59	88.3	2.90E-25	
AT4G30935.1	169	224	168	224	WRKY	2	60	59	62.2	3.90E-17	2
	332	388	331	388	WRKY	2	60	59	83.7	7.80E-24	
AT4G31550.1	247	303	246	304	WRKY	2	59	58	93.3	7.60E-27	1
AT4G31550.2	246	302	245	303	WRKY	2	59	58	93.3	7.60E-27	1
AT4G31800.1	176	233	176	234	WRKY	1	59	59	84.2	5.30E-24	1
AT4G31800.2	175	232	175	233	WRKY	1	59	59	84.2	5.30E-24	1
AT4G39410.1	223	279	223	280	WRKY	1	59	59	87.8	4.10E-25	1
AT5G01900.1	111	170	110	170	WRKY	2	60	61	79.2	1.90E-22	1
AT5G07100.1	117	174	117	174	WRKY	1	60	60	84.7	3.80E-24	2
	234	291	234	291	WRKY	1	60	60	86.8	8.40E-25	
AT5G07100.2	24	81	24	81	WRKY	1	60	60	85.5	2.00E-24	2
	141	198	141	198	WRKY	1	60	60	87.6	4.50E-25	
AT5G13080.1	67	124	67	124	WRKY	1	60	60	91.4	3.00E-26	1
AT5G15130.1	227	284	227	285	WRKY	1	59	59	93.1	9.10E-27	1
AT5G22570.1	111	170	110	170	WRKY	2	60	61	74	8.00E-21	1
AT5G24110.1	113	172	113	173	WRKY	1	59	61	87.4	5.30E-25	1
AT5G26170.1	113	169	113	170	WRKY	1	59	59	90.4	6.10E-26	1
AT5G28650.1	263	319	262	320	WRKY	2	59	58	91.5	2.80E-26	1
AT5G41570.1	98	155	98	155	WRKY	1	60	60	89.5	1.20E-25	1

	AT5G43290.1	114	170	114	171	WRKY	1	59	59	84.6	3.90E-24	1
	AT5G45050.1	1181	1238	1180	1238	WRKY	2	60	59	81.2	4.50E-23	1
	AT5G45050.2	1153	1210	1152	1210	WRKY	2	60	59	81.3	4.40E-23	1
	AT5G45260.1	1209	1268	1208	1268	WRKY	2	60	61	80.9	5.80E-23	1
	AT5G46350.1	183	240	183	240	WRKY	1	60	60	88.7	2.10E-25	1
	AT5G49520.1	221	278	221	278	WRKY	1	60	60	94.8	2.70E-27	1
	AT5G52830.1	166	223	165	223	WRKY	2	60	59	90.8	4.50E-26	1
	AT5G56270.1	273	328	273	329	WRKY	1	59	59	84.3	5.10E-24	2
		487	544	487	544	WRKY	1	60	60	86.7	8.90E-25	
	AT5G64810.1	110	167	110	167	WRKY	1	60	60	78.7	2.80E-22	1
**Basal**	***Amborella trichopoda* (33WRKYs)**											
**Magnoliophytas**	ERM93535	8	65	8	65	WRKY	1	60	60	78.6	3.00E-22	1
	ERM95579	249	305	249	305	WRKY	1	60	60	89.4	1.20E-25	2
		419	476	419	476	WRKY	1	60	60	89	1.70E-25	
	ERM95680	193	247	192	248	WRKY	2	59	58	86.9	7.70E-25	2
		388	445	388	445	WRKY	1	60	60	88.7	2.10E-25	
	ERM97637	89	146	88	146	WRKY	2	60	59	90.2	6.90E-26	1
	ERM99620	259	315	259	315	WRKY	1	60	60	80.5	7.80E-23	2
		437	494	437	494	WRKY	1	60	60	91.9	2.10E-26	
	ERM99929	139	196	139	196	WRKY	1	60	60	89.9	9.10E-26	1
	ERN00365	2	38	1	40	WRKY	21	58	38	41.7	1.00E-10	1
	ERN00368	235	290	234	292	WRKY	2	58	57	65.9	2.80E-18	1
	ERN01414	239	295	238	296	WRKY	2	59	58	95.8	1.30E-27	1
	ERN01868	199	255	199	255	WRKY	1	60	60	89.3	1.30E-25	2
		415	472	415	472	WRKY	1	60	60	87.2	6.00E-25	
	ERN02047	283	340	282	340	WRKY	2	60	59	91.2	3.60E-26	1
	ERN02112	122	179	121	179	WRKY	2	60	59	87.6	4.50E-25	1
	ERN03304	137	194	137	194	WRKY	1	60	60	91.3	3.30E-26	1
	ERN03816	252	310	252	310	WRKY	1	60	60	92.2	1.70E-26	1
	ERN03839	109	165	109	166	WRKY	1	59	59	84.5	4.30E-24	1
	ERN03881	140	196	140	197	WRKY	1	59	59	88.2	3.10E-25	1
	ERN04180	141	201	141	201	WRKY	1	60	62	86.1	1.40E-24	1
	ERN04181	6	65	6	65	WRKY	1	60	61	85.8	1.70E-24	1
	ERN05012	153	210	152	210	WRKY	2	60	59	89.2	1.50E-25	1
	ERN05172	7	46	4	46	WRKY	19	60	42	52.4	4.50E-14	1
	ERN05173	121	142	121	145	WRKY	1	22	22	34	2.50E-08	1
	ERN06521	226	281	226	282	WRKY	1	59	59	86.5	1.00E-24	2
		409	466	409	466	WRKY	1	60	60	88.5	2.50E-25	
	ERN07431	244	300	243	301	WRKY	2	59	58	94.2	3.90E-27	1
	ERN07935	111	167	111	168	WRKY	1	59	59	88.1	3.20E-25	1
	ERN08293	232	290	232	290	WRKY	1	60	60	94.6	3.10E-27	1
	ERN08862	266	323	265	323	WRKY	2	60	59	94.2	4.10E-27	1
	ERN08905	120	178	119	179	WRKY	2	59	60	89.9	8.80E-26	1
	ERN08906	120	178	119	179	WRKY	2	59	60	91	3.90E-26	1
	ERN09907	74	93	74	95	WRKY	1	20	20	29.2	8.10E-07	1
	ERN14755	208	265	207	265	WRKY	2	60	59	90.4	6.40E-26	1
	ERN18679	147	205	147	205	WRKY	1	60	60	84	6.30E-24	1
	ERN18680	113	170	112	170	WRKY	2	60	59	83.7	7.60E-24	1
	ERN19019	321	377	321	377	WRKY	1	60	60	88.9	1.90E-25	2

		536	593	536	593	WRKY	1	60	60	88	3.50E-25	
**Gymnosperms**	***Picea sitchensis* (8 WRKYs)**											
	gi|116791126|gb|ABK25866.1|	248	305	248	305	WRKY	1	60	60	89.8	9.70E-26	1
	gi|148905936|gb|ABR16129.1|	265	321	264	322	WRKY	2	59	58	93.2	8.10E-27	1
	gi|148906002|gb|ABR16161.1|	481	538	481	538	WRKY	1	60	60	89.2	1.50E-25	2
		284	338	283	339	WRKY	2	59	58	90.2	7.30E-26	
	gi|294461775|gb|ADE76446.1|	14	52	4	52	WRKY	20	60	42	42.2	6.70E-11	1
	gi|294463034|gb|ADE77055.1|	152	209	152	209	WRKY	1	60	60	93.3	7.60E-27	1
	gi|294463948|gb|ADE77495.1|	274	330	273	331	WRKY	2	59	58	91.9	2.10E-26	1
	gi|294464132|gb|ADE77584.1|	294	350	293	351	WRKY	2	59	58	93.3	7.70E-27	1
	gi|294464637|gb|ADE77827.1|	209	265	209	266	WRKY	1	59	59	89.4	1.20E-25	1
**Bryophytes**	***Physcomitrella patens* (117 WRKYs)**											
	Pp3c1_40230V3.1.p	326	383	325	383	WRKY	2	60	59	94.7	2.70E-27	1
	Pp3c1_40230V3.2.p	326	383	325	383	WRKY	2	60	59	94.7	2.70E-27	1
	Pp3c1_40230V3.3.p	326	383	325	383	WRKY	2	60	59	94.7	2.70E-27	1
	Pp3c11_14240V3.1.p	147	204	146	204	WRKY	2	60	59	91.3	3.30E-26	1
	Pp3c11_14240V3.2.p	147	204	146	204	WRKY	2	60	59	91.3	3.30E-26	1
	Pp3c11_1790V3.1.p	254	311	254	311	WRKY	1	60	60	83.3	1.00E-23	1
	Pp3c11_1790V3.2.p	254	311	254	311	WRKY	1	60	60	83.3	1.00E-23	1
	Pp3c11_1840V3.1.p	224	281	224	281	WRKY	1	60	60	88	3.50E-25	1
	Pp3c11_1840V3.2.p	224	281	224	281	WRKY	1	60	60	88	3.50E-25	1
	Pp3c11_1840V3.3.p	225	282	225	282	WRKY	1	60	60	88	3.60E-25	1
	Pp3c11_21340V3.2.p	176	196	175	207	WRKY	2	22	21	22	0.00014	1
	Pp3c12_4260V3.1.p	372	430	372	430	WRKY	1	60	60	89.5	1.20E-25	1
	Pp3c12_4260V3.2.p	372	430	372	430	WRKY	1	60	60	89.5	1.20E-25	1
	Pp3c13_10830V3.1.p	347	403	347	404	WRKY	1	59	59	88.6	2.30E-25	1
	Pp3c13_10830V3.2.p	347	403	347	404	WRKY	1	59	59	88.6	2.30E-25	1
	Pp3c13_15520V3.1.p	376	434	376	434	WRKY	1	60	60	94.5	3.30E-27	1
	Pp3c13_15520V3.2.p	376	434	376	434	WRKY	1	60	60	94.5	3.30E-27	1
	Pp3c13_15520V3.3.p	376	434	376	434	WRKY	1	60	60	94.5	3.30E-27	1
	Pp3c14_11320V3.1.p	196	252	195	253	WRKY	1	59	59	68.3	5.10E-19	1
	Pp3c14_11320V3.2.p	127	183	126	184	WRKY	1	59	59	68.6	3.90E-19	1
	Pp3c14_11320V3.3.p	196	252	195	253	WRKY	1	59	59	68.3	5.10E-19	1
	Pp3c14_17020V3.1.p	324	381	323	381	WRKY	2	60	59	93.6	6.00E-27	1
	Pp3c14_17020V3.2.p	324	381	323	381	WRKY	2	60	59	93.6	6.00E-27	1
	Pp3c17_19970V3.1.p	327	383	326	384	WRKY	2	59	58	92.6	1.30E-26	1
	Pp3c17_19970V3.2.p	327	383	326	384	WRKY	2	59	58	92.6	1.30E-26	1
	Pp3c19_3000V3.1.p	333	390	333	390	WRKY	1	60	60	88.3	2.70E-25	1
	Pp3c19_3000V3.2.p	333	390	333	390	WRKY	1	60	60	88.3	2.70E-25	1
	Pp3c2_23590V3.1.p	372	428	372	428	WRKY	1	60	60	84.8	3.40E-24	2
		551	608	551	608	WRKY	1	60	60	92.1	1.90E-26	
	Pp3c2_23590V3.2.p	372	428	372	428	WRKY	1	60	60	84.8	3.40E-24	2
		551	608	551	608	WRKY	1	60	60	92.1	1.90E-26	
	Pp3c2_23590V3.3.p	372	428	372	428	WRKY	1	60	60	84.8	3.40E-24	2
		554	611	554	611	WRKY	1	60	60	92.1	1.90E-26	
	Pp3c2_23590V3.4.p	372	428	372	428	WRKY	1	60	60	84.8	3.40E-24	2
		554	611	554	611	WRKY	1	60	60	92.1	1.90E-26	
	Pp3c2_23590V3.5.p	372	428	372	428	WRKY	1	60	60	84.5	4.30E-24	2
		554	611	554	611	WRKY	1	60	60	91.7	2.40E-26	

Pp3c2_23590V3.6.p	372	428	372	428	WRKY	1	60	60	84	6.00E-24	2
	554	611	554	611	WRKY	1	60	60	91.3	3.40E-26	
Pp3c2_2510V3.1.p	324	381	323	381	WRKY	2	60	59	94.7	2.70E-27	1
Pp3c2_2510V3.2.p	324	381	323	381	WRKY	2	60	59	94.7	2.70E-27	1
Pp3c2_2510V3.3.p	324	381	323	381	WRKY	2	60	59	94.7	2.70E-27	1
Pp3c2_2510V3.4.p	324	381	323	381	WRKY	2	60	59	94.7	2.70E-27	1
Pp3c2_2510V3.5.p	324	381	323	381	WRKY	2	60	59	94.7	2.70E-27	1
Pp3c2_2510V3.6.p	324	381	323	381	WRKY	2	60	59	94.7	2.70E-27	1
Pp3c2_2510V3.7.p	324	381	323	381	WRKY	2	60	59	94.7	2.70E-27	1
Pp3c2_32150V3.1.p	177	234	177	234	WRKY	1	60	60	79.4	1.70E-22	1
Pp3c2_32150V3.2.p	166	223	166	223	WRKY	1	60	60	79.5	1.60E-22	1
Pp3c2_32160V3.1.p	192	249	192	249	WRKY	1	60	60	85.3	2.40E-24	1
Pp3c2_32160V3.10.p	322	379	322	379	WRKY	1	60	60	84.8	3.40E-24	1
Pp3c2_32160V3.2.p	68	125	68	125	WRKY	1	60	60	86	1.50E-24	1
Pp3c2_32160V3.3.p	68	125	68	125	WRKY	1	60	60	86	1.50E-24	1
Pp3c2_32160V3.4.p	68	125	68	125	WRKY	1	60	60	86	1.50E-24	1
Pp3c2_32160V3.5.p	209	266	209	266	WRKY	1	60	60	85.2	2.50E-24	1
Pp3c2_32160V3.6.p	289	346	289	346	WRKY	1	60	60	84.9	3.10E-24	1
Pp3c2_32160V3.7.p	272	329	272	329	WRKY	1	60	60	85	3.00E-24	1
Pp3c2_32160V3.8.p	322	379	322	379	WRKY	1	60	60	84.8	3.40E-24	1
Pp3c2_32160V3.9.p	305	362	305	362	WRKY	1	60	60	84.9	3.20E-24	1
Pp3c2_9700V3.1.p	287	344	287	345	WRKY	1	59	60	70.5	1.00E-19	1
Pp3c2_9700V3.2.p	287	344	287	345	WRKY	1	59	60	70.5	1.00E-19	1
Pp3c2_9700V3.3.p	287	344	287	345	WRKY	1	59	60	70.5	1.00E-19	1
Pp3c2_9700V3.4.p	287	344	287	345	WRKY	1	59	60	70.5	1.00E-19	1
Pp3c2_9700V3.5.p	287	344	287	345	WRKY	1	59	60	70.5	1.00E-19	1
Pp3c2_9700V3.6.p	287	344	287	345	WRKY	1	59	60	70.5	1.00E-19	1
Pp3c20_12200V3.1.p	340	396	340	397	WRKY	1	59	59	89.8	9.80E-26	1
Pp3c20_12200V3.2.p	340	396	340	397	WRKY	1	59	59	89.8	9.80E-26	1
Pp3c20_12200V3.3.p	340	396	340	397	WRKY	1	59	59	89.8	9.80E-26	1
Pp3c21_11270V3.1.p	325	382	325	382	WRKY	1	60	60	86.1	1.40E-24	1
Pp3c21_11270V3.2.p	325	382	325	382	WRKY	1	60	60	86.1	1.40E-24	1
Pp3c22_12880V3.1.p	313	370	313	370	WRKY	1	60	60	84.6	4.00E-24	1
Pp3c23_10680V3.1.p	333	389	333	390	WRKY	1	59	59	89.7	1.00E-25	1
Pp3c23_10680V3.2.p	333	389	333	390	WRKY	1	59	59	89.7	1.00E-25	1
Pp3c24_7120V3.1.p	333	389	333	390	WRKY	1	59	59	90.4	6.10E-26	1
Pp3c24_7120V3.2.p	333	389	333	390	WRKY	1	59	59	90.4	6.10E-26	1
Pp3c26_5090V3.1.p	388	445	388	445	WRKY	1	60	60	88.4	2.50E-25	1
Pp3c26_5095V3.1.p	388	445	388	445	WRKY	1	60	60	88.4	2.50E-25	1
Pp3c27_600V3.1.p	149	206	149	207	WRKY	1	59	60	69.9	1.50E-19	1
Pp3c27_600V3.2.p	149	206	149	207	WRKY	1	59	60	69.9	1.50E-19	1
Pp3c27_600V3.3.p	243	300	243	301	WRKY	1	59	60	69.5	2.10E-19	1
Pp3c27_600V3.4.p	271	328	271	329	WRKY	1	59	60	69.3	2.30E-19	1
Pp3c27_600V3.5.p	271	328	271	329	WRKY	1	59	60	69.3	2.30E-19	1
Pp3c3_15040V3.1.p	251	308	251	308	WRKY	1	60	60	86.2	1.30E-24	1
Pp3c3_15040V3.2.p	251	308	251	308	WRKY	1	60	60	86.2	1.30E-24	1
Pp3c3_15040V3.3.p	251	308	251	308	WRKY	1	60	60	86.2	1.30E-24	1
Pp3c3_15040V3.4.p	311	368	311	368	WRKY	1	60	60	86	1.50E-24	1
Pp3c3_15040V3.5.p	311	368	311	368	WRKY	1	60	60	86	1.50E-24	1

	Pp3c3_15040V3.6.p	311	368	311	368	WRKY	1	60	60	86	1.50E-24	1
	Pp3c3_15040V3.7.p	357	414	357	414	WRKY	1	60	60	85.9	1.60E-24	1
	Pp3c3_21440V3.1.p	369	427	369	427	WRKY	1	60	60	93.3	7.80E-27	1
	Pp3c3_21440V3.2.p	369	427	369	427	WRKY	1	60	60	93.3	7.80E-27	1
	Pp3c3_27940V3.1.p	329	385	329	385	WRKY	1	60	60	89.3	1.40E-25	2
		492	549	492	549	WRKY	1	60	60	88.6	2.20E-25	
	Pp3c3_27940V3.2.p	329	385	329	385	WRKY	1	60	60	89.3	1.40E-25	2
		492	549	492	549	WRKY	1	60	60	88.6	2.20E-25	
	Pp3c3_27940V3.3.p	329	385	329	385	WRKY	1	60	60	89.3	1.40E-25	2
		492	549	492	549	WRKY	1	60	60	88.6	2.20E-25	
	Pp3c3_27940V3.4.p	329	385	329	385	WRKY	1	60	60	89.3	1.40E-25	2
		492	549	492	549	WRKY	1	60	60	88.6	2.20E-25	
	Pp3c3_32860V3.1.p	365	422	365	422	WRKY	1	60	60	85.5	2.10E-24	1
	Pp3c3_32860V3.2.p	365	422	365	422	WRKY	1	60	60	85.5	2.10E-24	1
	Pp3c3_32860V3.3.p	365	422	365	422	WRKY	1	60	60	85.5	2.10E-24	1
	Pp3c3_32860V3.4.p	365	422	365	422	WRKY	1	60	60	85.5	2.10E-24	1
	Pp3c3_32860V3.5.p	365	422	365	422	WRKY	1	60	60	85.5	2.10E-24	1
	Pp3c3_32860V3.6.p	365	422	365	422	WRKY	1	60	60	85.5	2.10E-24	1
	Pp3c3_8830V3.1.p	353	409	353	410	WRKY	1	59	59	85.6	2.00E-24	1
	Pp3c3_8830V3.2.p	353	409	353	410	WRKY	1	59	59	85.6	2.00E-24	1
	Pp3c4_15559V3.1.p	367	424	367	425	WRKY	1	59	59	89.8	9.30E-26	1
	Pp3c4_26880V3.1.p	334	390	334	391	WRKY	1	59	59	88.4	2.50E-25	1
	Pp3c4_26880V3.2.p	334	390	334	391	WRKY	1	59	59	88.4	2.50E-25	1
	Pp3c4_26880V3.3.p	334	390	334	391	WRKY	1	59	59	88.3	2.70E-25	1
	Pp3c4_26880V3.4.p	437	493	437	494	WRKY	1	59	59	88	3.40E-25	1
	Pp3c5_12130V3.1.p	349	406	349	407	WRKY	1	59	60	70	1.50E-19	1
	Pp3c5_12130V3.2.p	148	205	148	206	WRKY	1	59	60	70.9	7.70E-20	1
	Pp3c6_14470V3.1.p	343	400	343	401	WRKY	1	59	60	67.9	6.50E-19	1
	Pp3c6_14470V3.2.p	144	201	144	202	WRKY	1	59	60	69	3.00E-19	1
	Pp3c6_14470V3.3.p	144	201	144	202	WRKY	1	59	60	68.9	3.10E-19	1
	Pp3c7_18860V3.1.p	167	246	167	246	WRKY	1	60	85	48.6	6.80E-13	1
	Pp3c7_24490V3.1.p	216	273	216	273	WRKY	1	60	60	87.2	6.10E-25	1
	Pp3c7_24490V3.2.p	216	273	216	273	WRKY	1	60	60	87.2	6.10E-25	1
	Pp3c7_7550V3.1.p	316	372	315	373	WRKY	2	59	58	91.6	2.60E-26	1
	Pp3c7_7550V3.2.p	290	330	280	331	WRKY	18	59	42	47.3	1.70E-12	1
	Pp3c7_7550V3.3.p	316	372	315	373	WRKY	2	59	58	91.6	2.60E-26	1
	Pp3c8_4400V3.1.p	351	407	351	407	WRKY	1	60	60	89.6	1.10E-25	2
		514	571	514	571	WRKY	1	60	60	88.6	2.30E-25	
	Pp3c8_4400V3.2.p	351	407	351	407	WRKY	1	60	60	89.6	1.10E-25	2
		514	571	514	571	WRKY	1	60	60	88.6	2.30E-25	
	Pp3c8_4400V3.3.p	351	407	351	407	WRKY	1	60	60	89.6	1.10E-25	2
		514	571	514	571	WRKY	1	60	60	88.6	2.30E-25	
	Pp3c8_5110V3.1.p	372	429	372	429	WRKY	1	60	60	85.8	1.70E-24	1
	Pp3c8_5110V3.2.p	454	511	454	511	WRKY	1	60	60	85.6	2.00E-24	1
**Lycophytes**	***Selaginella moellendorffii* (35 WRKYs)**											
	EFJ04574	31	87	31	87	WRKY	1	59	59	95.5	1.60E-27	1
	EFJ07462	109	165	109	165	WRKY	1	60	60	92.9	1.00E-26	2
		225	282	225	282	WRKY	1	60	60	92.5	1.40E-26	
	EFJ08874	1	55	1	56	WRKY	2	59	58	81.5	3.60E-23	2

		117	173	117	174	WRKY	1	59	59	93.2	8.10E-27	
	EFJ09535	25	82	24	82	WRKY	2	60	59	95.4	1.70E-27	1
	EFJ10059	25	81	25	81	WRKY	1	59	59	92.2	1.70E-26	1
	EFJ10090	2	60	2	60	WRKY	1	60	60	101.9	1.60E-29	1
	EFJ11950	1	55	1	56	WRKY	2	59	58	81.3	4.40E-23	2
		117	173	117	174	WRKY	1	59	59	92.9	9.90E-27	
	EFJ14690	34	90	34	90	WRKY	1	59	59	93.6	6.30E-27	1
	EFJ17667	2	60	2	60	WRKY	1	60	60	98.7	1.50E-28	1
	EFJ18006	4	64	4	64	WRKY	1	60	62	96.2	9.40E-28	1
	EFJ20023	109	165	109	165	WRKY	1	60	60	92.9	1.00E-26	2
		225	282	225	282	WRKY	1	60	60	92.5	1.40E-26	
	EFJ20390	229	286	229	287	WRKY	1	59	59	92.8	1.10E-26	1
	EFJ20410	25	59	24	59	WRKY	2	36	35	60.1	1.80E-16	1
	EFJ20540	6	47	3	55	WRKY	4	46	43	46.3	3.60E-12	1
	EFJ21694	1	61	1	61	WRKY	1	60	62	92.3	1.50E-26	1
	EFJ23533	11	68	11	68	WRKY	1	60	60	94.3	3.80E-27	1
	EFJ25033	4	60	4	60	WRKY	1	60	60	97.3	4.40E-28	2
		111	168	111	168	WRKY	1	60	60	94.6	2.90E-27	
	EFJ26352	24	80	24	80	WRKY	1	59	59	94.1	4.40E-27	1
	EFJ28376	229	286	229	287	WRKY	1	59	59	92.8	1.10E-26	1
	EFJ28396	25	59	24	59	WRKY	2	36	35	60.1	1.80E-16	1
	EFJ28608	22	51	21	51	WRKY	7	36	30	45.8	5.30E-12	1
	EFJ29133	2	60	2	60	WRKY	1	60	60	98.7	1.50E-28	1
	EFJ30009	102	158	102	158	WRKY	1	60	60	96.1	1.00E-27	2
		209	266	209	266	WRKY	1	60	60	93.5	6.80E-27	
	EFJ31066	25	59	24	59	WRKY	2	36	35	60.1	1.80E-16	1
	EFJ31448	31	87	31	87	WRKY	1	59	59	95.5	1.60E-27	1
	EFJ33848	1	61	1	61	WRKY	1	60	62	94.1	4.30E-27	1
	EFJ33984	1	54	1	54	WRKY	6	60	55	86.6	9.70E-25	1
	EFJ34648	25	59	24	59	WRKY	2	36	35	60.1	1.80E-16	1
	EFJ34797	5	60	5	61	WRKY	1	59	59	88.1	3.20E-25	2
		174	231	174	231	WRKY	1	60	60	89.9	8.60E-26	
	EFJ34902	2	60	2	60	WRKY	1	60	60	101.9	1.60E-29	1
	EFJ34931	26	82	26	82	WRKY	1	59	59	92.1	1.80E-26	1
	EFJ36328	25	82	24	82	WRKY	2	60	59	95.4	1.70E-27	1
	EFJ37089	4	64	4	64	WRKY	1	60	62	96.2	9.40E-28	1
	EFJ37492	1	54	1	54	WRKY	6	60	55	86.6	9.70E-25	1
	EFJ38698	31	87	31	87	WRKY	1	59	59	95.5	1.60E-27	1
**Chlorophytes**	***Ostreococcus lucimarinu* (2 WRKYs)**											
	ABO97433	27	88	27	89	WRKY	1	59	65	60.3	1.60E-16	1
	ABO98812	46	104	45	105	WRKY	2	59	60	85	3.00E-24	1
	***Ostreococcus tauri* (6 WRKYs)**											
	gi|116059246|emb|CAL54953.1|	23	83	23	83	WRKY	1	60	64	72.8	1.90E-20	1
	gi|308806431|ref|XP_003080527.1|	82	139	82	140	WRKY	1	55	61	58.6	5.20E-16	1
	gi|308806948|ref|XP_003080785.1|	23	83	23	83	WRKY	1	60	64	72.8	1.90E-20	1
	gi|308809517|ref|XP_003082068.1|	31	94	31	94	WRKY	1	60	65	84.5	4.20E-24	1
	gi|693497948|emb|CEF99688.1|	31	94	31	94	WRKY	1	60	65	84.4	4.50E-24	1
	gi|693499331|emb|CEF98869.1|	141	198	141	199	WRKY	1	55	61	57.9	8.90E-16	1
	***Micromonas pusilla CCMP1545* (2 WRKYs)**											

50253	67	119	67	122	WRKY	1	55	55	76.2	1.70E-21	1
62997	5	60	5	60	WRKY	1	60	60	85.4	2.20E-24	1
***Volvox carteri* (4 WRKYs)**											
Vocar.0001s1791.1.p	122	178	121	178	WRKY	2	60	59	80.3	8.60E-23	2
	357	414	357	414	WRKY	1	60	60	91.1	3.80E-26	
Vocar.0001s1791.2.p	121	177	120	177	WRKY	2	60	59	80.3	8.60E-23	2
	356	413	356	413	WRKY	1	60	60	91.1	3.80E-26	
Vocar.0058s0025.1.p	1129	1165	1127	1204	WRKY	3	42	40	31.8	1.20E-07	1
Vocar.0072s0004.1.p	1230	1264	1228	1269	WRKY	3	40	38	29.9	4.80E-07	1
***Coccomyxa subellipsoidea C_169* (1 WRKY)**											
8449	2	58	2	59	WRKY	1	59	59	74.6	5.40E-21	1
