# Supplementary material for: Genome-wide analysis of WRKY transcription factors in wheat (Triticum aestivum L.) and differential expression under water deficit condition
Source: PeerJ. 2017 May 4;5:e3232. doi: 10.7717/peerj.3232 (PMC5420200; doi:10.7717/peerj.3232)
Supplement: Table S3 [file peerj-05-3232-s005.pdf]

Supplemental Table S3. Sequence features of WRKYs identified in wheat.

Group	Proposed name	Transcript ID	Chr	NA (aa)	MW(KDa)	p*I*	Ai	GRAVY	II	Conserved motif	Domain Pattern	Zinc-finger structure
I	TaWRKY1	Traes_1AS_F3EAEC435.1	1AS	440	47.5416	7.98	58.09	-0.688	49.03	2×[WRKYGQK]	C-X_4-C-X_{23}-HDH(N) C-X_4-C-X_{23}-HSH(C)	C_2H_2
I	TaWRKY8	Traes_1BS_EF67E5A24.1	1BS	458	49.3004	8.01	56.66	-0.691	48.81	2×[WRKYGQK]	C-X_4-C-X_{23}-HDH(N) C-X_4-C-X_{23}-HSH(C)	C_2H_2
I	TaWRKY23	Traes_2AS_C407071E4.2	2AS	571	62.3895	6.69	53.78	-0.777	48.15	2×[WRKYGQK]	C-X_4-C-X_{22}-HNH(N) C-X_4-C-X_{23}-HNH(C)	C_2H_2
I	TaWRKY25	Traes_2AS_0186B9E4F.2	2AS	421	46.0009	6.43	66.94	-0.734	40.43	2×[WRKYGQK]	C-X_3-C-X_{22}-HCH(N) C-X_4-C-X_{23}-HTH(C)	C_2H_2
I	TaWRKY26	Traes_2AL_409AB7647.1	2AL	733	77.3186	6.39	54.27	-0.705	49.63	2×[WRKYGQK]	C-X_4-C-X_{22}-HNH(N) C-X_4-C-X_{23}-HNH(C)	C_2H_2
I	TaWRKY30	Traes_2BL_2BA3A755A.1	2BL	729	UN	UN	53.1	-0.736	51.47	2×[WRKYGQK]	C-X_4-C-X_{22}-HNH(N) C-X_4-C-X_{23}-HNH(C)	C_2H_2
I	TaWRKY32	Traes_2BS_380EC4D1E.1	2BS	468	50.688	5.84	65.47	-0.676	43.94	2×[WRKYGQK]	C-X_3-C-X_{22}-HCH(N) C-X_4-C-X_{23}-HTH(C)	C_2H_2
I	TaWRKY33	Traes_2BS_D435A8999.1	2BS	524	57.031	6.35	50.08	-0.888	49.38	2×[WRKYGQK]	C-X_4-C-X_{22}-HNH(N) C-X_4-C-X_{23}-HNH(C)	C_2H_2
I	TaWRKY39	Traes_2DS_97E3E7CFC.1	2DS	525	57.2143	6.26	50.72	-0.884	48.45	2×[WRKYGQK]	C-X_4-C-X_{22}-HNH(N) C-X_4-C-X_{23}-HNH(C)	C_2H_2
I	TaWRKY41	Traes_2DS_F6FBC974C.2	2DS	373	40.1606	5.61	73.99	-0.558	33.62	2×[WRKYGQK]	C-X_3-C-X_{22}-HCH(N) /(C)	C_2H_2
I	TaWRKY69	Traes_4BL_EFEC50B26.2	4BL	311	34.2057	7.75	63.92	-0.827	40.44	2×[WRKYGQK]	C-X_3-C-X_{22}-HNH(N) /(C)	C_2H_2
I	TaWRKY75	Traes_4DS_DC3C9DC42.2	4DS	485	51.6168	6.97	58.43	-0.658	53.13	2×[WRKYGQK]	C-X_4-C-X_{22}-HNH(N) C-X_4-C-X_{23}-HSH(C)	C_2H_2
I	TaWRKY77	Traes_5AL_ED3ADED51.3	5AL	590	64.1371	5.86	53.42	-0.833	52.76	2×[WRKYGQK]	C-X_4-C-X_{22}-HNH(N) C-X_4-C-X_{23}-HNH(C)	C_2H_2
I	TaWRKY82	Traes_5BS_C46781248.1	5BL	355	39.0837	8.12	49.24	-1.085	54.52	2×[WRKYGQK]	C-X_4-C-X_{22}-HNH(N) C-X_4-C-X_{23}-HNH(C)	C_2H_2
I	TaWRKY83	Traes_5BL_A522C62D1.1	5BL	333	36.4387	8.94	58.56	-0.621	49.04	2×[WRKYGQK]	C-X_4-C-X_{22}-HNH(N) C-X_4-C-X_{21}-HNH(C)	C_2H_2
I	TaWRKY88	Traes_5BL_17A712C94.1	5BL	679	73.6722	5.91	50.9	-0.773	55.17	2×[WRKYGQK]	C-X_4-C-X_{22}-HNH(N) C-X_4-C-X_{23}-HNH(C)	C_2H_2
I	TaWRKY94	Traes_5DS_5DEA5C9E3.1	5DS	497	52.8289	8.12	50.93	-0.85	60.28	2×[WRKYGQK]	C-X_4-C-X_{22}-HNH(N) C-X_4-C-X_{23}-HNH(C)	C_2H_2
I	TaWRKY96	Traes_5DL_21F7C6BF7.2	5DL	571	62.0741	5.79	57.55	-0.782	50.52	2×[WRKYGQK]	C-X_4-C-X_{22}-HNH(N) C-X_4-C-X_{23}-HNH(C)	C_2H_2
I	TaWRKY98	Traes_5DL_46E3AC8D6.1	5DL	346	38.1112	7.79	51.42	-0.886	65.46	2×[WRKYGQK]	C-X_4-C-X_{22}-HTH(N) /(C)	C_2H_2
I	TaWRKY99	Traes_5DL_4BA2CC560.2	5DL	334	36.5949	9.02	55.48	-0.68	51.14	2×[WRKYGQK]	C-X_4-C-X_{22}-HNH(N) C-X_4-C-X_{21}-HNH(C)	C_2H_2
I	TaWRKY109	Traes_7BL_A46F1A830.2	7BL	305	33.6641	9.37	70.36	-0.587	62.03	2×[WRKYGQK]	C-X_4-C-X_{22}-HTH(N) /(C)	C_2H_2
I	TaWRKY111	Traes_7DS_24C563960.1	7DS	305	33.6741	9.31	70.98	-0.58	63.82	2×[WRKYGQK]	C-X_4-C-X_{22}-HSH(N) /(C)	C_2H_2
I	TaWRKY120	Traes_1DS_A6733B734.1	1DS_scaff_750830	464	49.9713	8.56	56.79	-0.692	49.37	2×[WRKYGQK]	C-X_4-C-X_{23}-HDH(N) C-X_4-C-X_{23}-HSH(C)	C_2H_2
I	TaWRKY133	Traes_3B_990298FF5.1	3B_scaff_10750391	559	59.5938	6.11	41.16	-0.808	58.04	2×[WRKYGQK]	C-X_4-C-X_{22}-HNH(N) C-X_4-C-X_{23}-HNH(C)	C_2H_2
I	TaWRKY141	Traes_4AL_2EEECCC4B.1	4AL_scaff_7093101	376	40.9167	7.71	58.94	-0.873	48.56	2×[WRKYGQK]	C-X_4-C-X_{22}-HNH(N) C-X_4-C-X_{23}-HSH(C)	C_2H_2
I	TaWRKY145	Traes_5AL_E644A6A0B.1	5AL_scaff_2685980	333	36.5127	9.13	55.35	-0.654	50.4	2×[WRKYGQK]	C-X_4-C-X_{22}-HNH(N) C-X_4-C-X_{21}-HNH(C)	C_2H_2
I	TaWRKY150	Traes_5AS_9C6171380.1	5AS_scaff_1020534	360	39.5482	8.12	48	-1.074	55.13	2×[WRKYGQK]	C-X_4-C-X_{22}-HNH(N) C-X_4-C-X_{23}-HNH(C)	C_2H_2
I	TaWRKY152	Traes_5BL_D3C383CF5.1	5BL_scaff_10787947	341	37.3634	6.93	53.31	-0.872	68.78	2×[WRKYGQK]	/(N) C-X_4-C-X_{22}-HNH(C)	C_2H_2
I	TaWRKY168	Traes_7AL_48C81DE03.1	7AL_scaff_4556343	395	UN	UN	64.2	-0.596	56.1	2×[WRKYGQK]	C-X_4-C-X_{22}-HSH(N) /(C)	C_2H_2
I	TaWRKY171	Traes_7AL_48C81DE031.1	7AL_scaff_4556343	395	UN	UN	64.2	-0.596	56.1	2×[WRKYGQK]	C-X_4-C-X_{22}-HSH(N) /(C)	C_2H_2
I-III	TaWRKY157	Traes_5DL_1733FB4DA.1	5DL_scaff_4534545	1,100	123.464	6.3	87.46	-0.311	47.25	WRKYGQK(N)	C-X_7-C-X_{23}-HTC(N)	C_2HC
										WSKYGQK(C)	C-X_7-C-X_{23}-HTC(C)	C_2HC
IIa	TaWRKY86	Traes_5BL_8BEF7F9CD.1	5BL	330	35.639	7.95	60.94	-0.593	56.37	WRKYGQK	C-X_4-C-X_{23}-HNH	C_2H_2
IIa	TaWRKY87	Traes_5BL_90757F0CC.1	5BL	331	35.9063	7.49	62.21	-0.602	46.42	WRKYGQK	C-X_5-C-X_{23}-HNH	C_2H_2
IIa	TaWRKY113	Traes_7DL_5968FA56C.1	7DL	197	21.1439	9.42	71.78	-0.497	38.96	WRKYGQK	C-X_5-C-X_{23}-HNH	C_2H_2
IIa	TaWRKY114	Traes_7DL_B09854286.1	7DL	180	19.6342	9.27	59.11	-0.741	41.33	WRKYGQK	C-X_5-C-X_{23}-HNH	C_2H_2
IIa	TaWRKY115	Traes_7DL_F849918EA.2	7DL	164	17.5338	9.64	51.83	-0.691	53.26	WRKYGQK	C-X_5-C-X_{23}-HNH	C_2H_2
IIa	TaWRKY144	Traes_5AL_7164FEAC3.1	5AL_scaff_2204788	83	9.3136	9.78	58.55	-0.812	29.32	WRKYGQK	/	C_2H_2
IIa	TaWRKY156	Traes_5DL_32D78D06A.1	5DL_scaff_4501324	105	11.8102	9.33	57.43	-0.91	31.12	WRKYGQK	C-X_4-C-X_{23}-HNH	C_2H_2
IIa	TaWRKY161	Traes_6AS_DA75BB1FD.1	6AS_scaff_4428654	300	32.6184	8.44	55.33	-0.874	58.15	WRKYGQK	C-X_5-C-X_{23}-HNH	C_2H_2
IIa	TaWRKY165	Traes_6DS_8F684013D.2	6DS_scaff_1013038	139	15.3522	9.03	49.86	-0.945	64.74	WRKYGQK	C-X_5-C-X_{23}-HNH	C_2H_2
IIa	TaWRKY167	Traes_7AL_AC56CC184.1	7AL_scaff_4376169	91	10.2176	9.35	60.88	-0.703	29.54	WRKYGQK	/	C_2H_2
IIa	TaWRKY170	Traes_7DL_B86B36F67.1	7DL_scaff_3340826	155	UN	UN	62.97	-0.654	38.14	WRKYGQK	C-X_5-C-X_{23}-HNH	C_2H_2
IIb	TaWRKY47	TRAES3BF003800010CFD_t1	3BF	576	60.2689	6.71	58.28	-0.579	51.74	WRKYGQK	C-X_5-C-X_{23}-HNH	C_2H_2
IIb	TaWRKY68	Traes_4AL_3E11167D9.1	4AL	262	28.9792	8.73	57.33	-0.99	30.82	WRKYGQK	/	C_2H_2
IIb	TaWRKY104	Traes_6AL_0C0899C15.1	6AL	553	57.7713	5.01	56.38	-0.709	49.12	WRKYGQK	C-X_5-C-X_{23}-HNH	C_2H_2
IIb	TaWRKY108	Traes_7AS_C9DF68E53.2	7AS	451	47.8442	8.98	56.81	-0.549	42.25	WRKYGQK	C-X_5-C-X_{23}-HNH	C_2H_2
IIb	TaWRKY131	Traes_3AS_5CD024A9E.1	3AS_scaff_794031	78	8.8943	9.98	55.13	-0.742	38.23	WRKYGQK	C-X_5-C-X_{23}-HNH	C_2H_2
IIb	TaWRKY162	Traes_6BL_B92FA1D38.1	6BL_scaff_4134725	58	6.6656	9.17	47.07	-0.826	48.93	WRKYGQK	C-X_5-C-X_{23}-HNH	C_2H_2
IIb	TaWRKY164	Traes_6DL_AB95B0CE0.1	6DL_scaff_3269795	559	58.5503	4.96	58.21	-0.682	51.77	WRKYGQK	C-X_5-C-X_{23}-HNH	C_2H_2
IIc	TaWRKY2	Traes_1AS_1432A2F79.1	1AS	123	13.7303	9.61	50.81	-0.956	37.44	WRKYGKK	C-X_4-C-X_{23}-HTH	C_2H_2
IIc	TaWRKY6	Traes_1AL_0404BC790.1	1AL	135	14.9779	10.02	55.63	-0.847	38.07	WRKYGQK	C-X_4-C-X_{23}-HNH	C_2H_2
IIc	TaWRKY7	Traes_1AL_9ADA7A031.1	1AL	220	23.0964	6.96	57.86	-0.355	50.94	WRKYGKK	C-X_4-C-X_{23}-HAH	C_2H_2
IIc	TaWRKY11	Traes_1BL_1D865A8CC.1	1BL	67	7.928	7.93	82.84	-0.494	40.35	/	C-X_4-C-X_{23}-HGH	C2H2
IIc	TaWRKY12	Traes_1BL_9AFA4B870.1	1BL	321	33.6695	9.07	49.78	-0.672	37.97	WRKYGQK	C-X_4-C-X_{23}-HGH	C_2H_2
IIc	TaWRKY15	Traes_1DL_D550418641.2	1DL	96	UN	UN	29.38	-1.057	26.13	WRKYGKK	C-X_4-C-X_{23}-HTH	C_2H_2
IIc	TaWRKY16	Traes_1DL_5BAB0B6BC.1	1DL	135	14.9318	10.02	59.26	-0.747	31.74	WRKYGQK	C-X_4-C-X_{23}-HCH	C_2H_2
IIc	TaWRKY17	Traes_1DL_D1EC7DEA6.1	1DL	112	12.6058	9.3	37.41	-1.387	53.36	WRKYGQK	/	C_2H_2
IIc	TaWRKY18	Traes_1DL_46428511F.1	1DL	174	19.2655	9.62	49.43	-0.847	31.69	WRKYGQK	C-X_4-C-X_{23}-HNH	C_2H_2
IIc	TaWRKY19	Traes_2AS_D0C21ADB5.1	2AS	126	13.4795	8.98	38.73	-1.133	43.25	WRKYGQK	C-X_7-C-X_{23}-HLH	C_2H_2
IIc	TaWRKY20	Traes_2AL_1B43EA59E.1	2AL	228	24.7367	9.3	52.37	-0.676	58.57	WRKYGQK	C-X_4-C-X_{23}-HLH	C_2H_2
IIc	TaWRKY21	Traes_2AL_B1270662B.1	2AL	229	25.0493	8.79	53.32	-0.576	47.47	WRKYGQK	/	C_2H_2
IIc	TaWRKY28	Traes_2BS_F3097F116.1	2BS	204	22.5485	8.53	45.34	-1.032	38.81	WRKYGQK	C-X_4-C-X_{23}-HTH	C_2H_2
IIc	TaWRKY31	Traes_2BL_A69F6C5DF.1	2BL	285	30.3388	6.45	56	-0.507	59.51	WRKYGQK	/	C_2H_2
IIc	TaWRKY34	Traes_2BL_A5BFA97B9.1	2BL	229	25.0633	8.79	56.24	-0.58	51.68	WRKYGQK	/	C_2H_2
IIc	TaWRKY36	Traes_2DS_0F2500A60.1	2DS	191	21.0789	9.08	46.39	-1.052	36.24	WRKYGQK	C-X_4-C-X_{22}-HQH	C_2H_2
IIc	TaWRKY38	Traes_2DL_4F9F8F1F0.1	2DL	229	24.9631	8.79	54.59	-0.573	46.89	WRKYGQK	C-X_4-C-X_{22}-HQH	C_2H_2
IIc	TaWRKY42	Traes_3AL_67ECA2932.1	3AL	49	5.7535	10.16	43.88	-1.227	52.47	WRKYGKK	C-X_4-C-X_{23}-HCH	C_2H_2
IIc	TaWRKY43	Traes_3AL_1B73D2C12.1	3AL	79	9.0972	8.62	59.24	-0.556	35.67	/	C-X_{4-}C-X_{23}-HSH	C_2H_2
IIc	TaWRKY45	TRAES3BF066700160CFD_t1	3BF	222	23.8954	6.59	51.13	-0.445	46.8	WRKYGKK	C-X_4-C-X_{23}-HNH	C_2H_2
IIc	TaWRKY48	TRAES3BF021300010CFD_t1	3BF	346	37.7506	6.64	51.04	-0.924	60.39	WRKYGQK	C-X_6-F-X_{23}-HNH	CFH_2

IIc	TaWRKY49	TRAES3BF267200010CFD_t1	3BF	239	26.2649	7.04	51.51	-0.822	55.51	WRKYGKK	/	C_2H_2
IIc	TaWRKY50	TRAES3BF021100090CFD_t1	3BF	346	37.7506	6.64	51.04	-0.924	60.39	WRKYGQK	$C-X_4-C-X_{23}-HGH$	C_2H_2
IIc	TaWRKY52	TRAES3BF058500060CFD_t1	3BF	368	38.6751	8.5	53.94	-0.634	47.84	WRKYGQK	/	C_2H_2
IIc	TaWRKY53	TRAES3BF111700140CFD_t1	3BF	200	21.0834	7.71	57.6	-0.438	42.82	WRKYGKK	$C-X_4-C-X_{23}-HNH$	C_2H_2
IIc	TaWRKY61	TRAES3BF090100100CFD_t1	3BF	312	33.0295	6.26	63.91	-0.536	47.55	WRKYGQK	$C-X_4-C-X_{23}-HCH$	C_2H_2
IIc	TaWRKY62	TRAES3BF045500040CFD_t1	3BF	410	43.6012	5.34	58.39	-0.505	61.62	WRKYGQK	$C-X_4-C-X_{23}-HTH$	C_2H_2
IIc	TaWRKY63	Traes_3DL_DF0D3F3FE.1	3DL	90	10.3644	8.56	45.44	-1.066	41.52	WRKYGKK	$C-X_4-C-X_{23}-HTH$	C_2H_2
IIc	TaWRKY79	Traes_5AL_E566BD64E.1	5AL	66	UN	UN	51.67	-0.948	29.81	/	$C-X_4-C-X_{23}-HCH$	C_2H_2
IIc	TaWRKY90	Traes_5BL_C1D6B6B74.2	5BL	310	UN	UN	51.45	-0.496	60.74	WRKYGQK	$C-X_4-C-X_{23}-HTH$	C_2H_2
IIc	TaWRKY97	Traes_5DL_7E2053226.2	5DL	208	23.1771	9.78	56.2	-0.684	74.72	WRKYGQK	$C-X_4-C-X_{23}-HTH$	C_2H_2
IIc	TaWRKY102	Traes_6AL_BA4636569.1	6AL	236	26.317	8.61	57.8	-0.52	51.97	WRKYGQK	$C-X_4-C-X_{23}-HTH$	C_2H_2
IIc	TaWRKY105	Traes_6BL_DD840863A.1	6BL	235	26.2969	8.46	55.15	-0.537	53.62	WRKYGQK	$C-X_4-C-X_{23}-HTH$	C_2H_2
IIc	TaWRKY106	Traes_6DL_D29E210A1.1	6DL	236	26.423	8.46	55.72	-0.538	56.77	WRKYGQK	$C-X_4-C-X_{23}-HTH$	C_2H_2
IIc	TaWRKY116	Traes_1AL_F64E07A92.1	1AL_scaff_3884747	168	18.7661	9.66	56.85	-0.666	38.98	WRKYGQK	/	C_2H_2
IIc	TaWRKY117	Traes_1AL_180B9CF01.1	1AL_scaff_3934462	205	22.6461	9.25	48.63	-0.961	48.02	WRKYGQK	$C-X_4-C-X_{23}-HNH$	C_2H_2
IIc	TaWRKY118	Traes_1BL_B15990028.1	1BL_scaff_3848213	170	19.0303	9.6	52.24	-0.838	35.77	WRKYGQK	$C-X_4-C-X_{23}-HNH$	C_2H_2
IIc	TaWRKY123	Traes_2DL_04535D371.1	2DL_scaff_9908519	285	30.3569	6.4	57.37	-0.485	55.74	WRKYGQK	$C-X_4-C-X_{23}-HTH$	C_2H_2
IIc	TaWRKY124	Traes_3AL_DED8A29EC.1	3AL_scaff_382150	73	7.8144	10.06	22.88	-1.581	28.95	WRKYGQK	$C-X_4-C-X_{23}-HDH$	C_2H_2
IIc	TaWRKY125	Traes_3AL_4769A72F1.1	3AL_scaff_805190	57	6.5586	9.22	85.44	-0.382	28.61	/	$C-X_4-C-X_{23}-HTH$	C_2H_2
IIc	TaWRKY127	Traes_3AL_F326C5B8E.1	3AL_scaff_3442799	98	11.2758	9.56	52.65	-1.02	50.91	WRKYGQK	$C-X_4-C-X_{23}-HTH$	C_2H_2
IIc	TaWRKY128	Traes_3AL_3160E1F30.1	3AL_scaff_4247568	62	7.2742	9.43	37.74	-1.223	24.71	WRKYGQK	$C-X_4-C-X_{23}-HNH$	C_2H_2
IIc	TaWRKY129	Traes_3AL_AB2BAE660.1	3AL_scaff_4270257	155	16.9304	7.7	46.71	-0.859	55.59	WRKYGKK	$C-X_4-C-X_{23}-HNH$	C_2H_2
IIc	TaWRKY130	Traes_3AL_140B829CB.2	3AL_scaff_4308486	345	37.6536	7.1	49.77	-0.919	58.82	WRKYGQK	$C-X_4-C-X_{23}-HLH$	C_2H_2
IIc	TaWRKY138	Traes_3DS_F6B1E6078.1	3DS_scaff_2602154	63	7.753	10.25	47.78	-1.332	29.6	WRKYGQK	$C-X_4-C-X_{23}-HLH$	C_2H_2
IIc	TaWRKY139	Traes_4AL_C2A825B6D.1	4AL_scaff_3841042	81	9.1644	10.2	55.43	-0.935	29.36	WRKYGKK	$C-X_4-C-X_{23}-HNH$	C_2H_2
IIc	TaWRKY140	Traes_4AL_234E1CDF6.1	4AL_scaff_7067843	157	17.5236	6.51	62.8	-0.699	39.79	WRKYGQK	$C-X_4-C-X_{23}-HSH$	C_2H_2
IIc	TaWRKY143	Traes_4DS_CFC487CE5.2	4DS_scaff_2286750	145	16.2729	7.73	42.48	-1.07	44.34	WRKYGQK	$C-X_4-C-X_{23}-HNH$	C_2H_2
IIc	TaWRKY149	Traes_5AL_06A6F9328.2	5AL_scaff_2793480	193	UN	UN	37.82	-0.728	46.64	WRKYGQK	$C-X_4-C-X_{23}-HCH$	C_2H_2
IIc	TaWRKY166	Traes_6DS_BF71C1557.2	6DS_scaff_2049506	254	26.8369	9.02	91.77	0.147	42.39	/	$C-X_4-C-X_{23}-HCH$	C_2H_2
IId	TaWRKY22	Traes_2AL_434E9F101.1	2AL	313	33.0191	9.43	56.81	-0.541	52.61	WRKYGQK	$C-X_5-C-X_{23}-HRH$	C_2H_2
IId	TaWRKY27	Traes_2AL_15A7BB684.1	2AL	328	34.5968	10.09	68.72	-0.344	56.71	WRKYGQK	$C-X_5-C-X_{23}-HRH$	C_2H_2
IId	TaWRKY35	Traes_2BL_6B75B32E3.1	2BL	313	33.032	10.02	65.18	-0.384	55.35	WRKYGQK	$C-X_5-C-X_{23}-HRH$	C_2H_2
IId	TaWRKY37	Traes_2DL_F600B5FDF.1	2DL	120	13.1693	9.87	54.42	-0.595	45.78	WRKYGQK	$C-X_5-C-X_{23}-HRH$	C_2H_2
IId	TaWRKY67	Traes_4AL_98B1C762B.1	4AL	349	38.0377	9.84	58.17	-0.719	52.12	WRKYGQK	$C-X_5-C-X_{23}-HNH$	C_2H_2
IId	TaWRKY71	Traes_4BS_CE839571B.2	4BL	342	37.3424	9.93	64.47	-0.62	51.65	WRKYGQK	/	C_2H_2
IId	TaWRKY73	Traes_4DS_3BE557D5C.2	4DS	342	37.4675	9.82	63.63	-0.613	50.92	WRKYGQK	/	C_2H_2
IId	TaWRKY80	Traes_5AL_6F7D1D441.1	5AL	124	13.8386	9.44	47.34	-1.078	39.35	WRKYGQK	$C-X_5-Y-X_{23}-HNH$	C_2H_2
IId	TaWRKY81	Traes_5BS_E0345D5DF.2	5BL	355	38.1304	10.17	61.32	-0.585	62.09	WRKYGQK	$C-X_5-C-X_{23}-HSH$	C_2H_2
IId	TaWRKY89	Traes_5BL_8688F70C9.1	5BL	299	31.0712	10.06	63.21	-0.427	45.74	WRKYGQK	$C-X_5-C-X_{23}-HRH$	C_2H_2
IId	TaWRKY91	Traes_5BL_E294922A9.2	5BL	379	41.0487	9.19	63.69	-0.476	65.23	WRKYGQK	$C-X_5-C-X_{23}-HNH$	C_2H_2
IId	TaWRKY93	Traes_5DS_D83DEA9B0.1	5DS	356	38.2725	10.22	60.87	-0.608	63.19	WRKYGQK	$C-X_5-C-X_{23}-HSH$	C_2H_2
IId	TaWRKY148	Traes_5AL_A3653B781.1	5AL_scaff_2786992	119	13.3252	10	51.76	-0.963	50.29	WRKYGQK	$C-X_5-C-X_{23}-HNH$	C_2H_2
IId	TaWRKY151	Traes_5AS_433D3E526.1	5AS_scaff_1136374	136	15.1134	9.97	55.96	-0.914	46.4	WRKYGQK	$C-X_5-C-X_{23}-HSH$	C_2H_2
IId	TaWRKY154	Traes_5BL_AEF9FE805.2	5BL_scaff_10827243	400	42.9107	10.05	59.85	-0.601	55.82	WRKYGQK	$C-X_5-C-X_{23}-HNH$	C_2H_2
IId	TaWRKY158	Traes_5DL_2553A6C33.1	5DL_scaff_4566006	115	UN	UN	46.7	-0.691	31.7	WRKYGQK	$C-X_5-C-X_{23}-HNH$	C_2H_2
IId	TaWRKY159	Traes_5DL_A54ED44C9.2	5DL_scaff_4573934	394	42.2809	10	60.25	-0.594	57.14	WRKYGQK	$C-X_5-C-X_{23}-HNH$	C_2H_2
IIe	TaWRKY3	Traes_1AL_4E924201A.1	1AL	342	36.7389	6.19	61.96	-0.591	51.95	WRKYGQK	$C-X_5-C-X_{23}-HNH$	C_2H_2
IIe	TaWRKY51	TRAES3BF180700010CFD_t1	3BF	319	34.5208	6.18	64.89	-0.581	55.38	WRKYGQK	$C-X_5-C-X_{23}-HCH$	C_2H_2
IIe	TaWRKY54	TRAES3BF073300120CFD_t1	3BF	297	31.1866	5.34	58.48	-0.539	56.79	WRKYGQK	$C-X_5-C-X_{23}-HNH$	C_2H_2
IIe	TaWRKY55	TRAES3BF029000080CFD_t1	3BF	288	31.4786	4.98	47.15	-0.961	80.15	WRKYGQK	$C-X_5-C-X_{23}-HNH$	C_2H_2
IIe	TaWRKY100	Traes_5DL_E4A6D1889.2	5DL	366	39.6035	7.2	61.97	-0.501	56.09	WRKYGQK	$C-X_5-C-X_{23}-HNH$	C_2H_2
IIe	TaWRKY103	Traes_6AL_A5FB7CFA5.1	6AL	481	50.4121	5.83	52.91	-0.471	49.23	WRKYGQK	$C-X_5-C-X_{23}-HNH$	C_2H_2
IIe	TaWRKY107	Traes_6DL_D4F2CDDDC.1	6DL	481	50.5001	5.72	52.89	-0.477	48.83	WRKYGQK	$C-X_5-C-X_{23}-HNH$	C_2H_2
IIe	TaWRKY119	Traes_1BL_794E99FF5.1	1BL_scaff_3919848	341	36.8072	6.59	64.99	-0.535	49.34	WRKYGQK	$C-X_5-C-X_{23}-HNH$	C_2H_2
IIe	TaWRKY155	Traes_5BL_B9DD3E76F.1	5BL_scaff_10924584	371	39.9227	6.63	58.25	-0.547	60.38	WRKYGQK	$C-X_4-C-X_{23}-HNH$	C_2H_2
IIe	TaWRKY163	Traes_6BL_EEAA2A7E3.1	6BL_scaff_4221964	146	15.9801	10.56	48.84	-0.941	53.27	WRKYGQK	$C-X_5-C-X_{23}-HNH$	C_2H_2
III	TaWRKY4	Traes_1AL_309623B48.1	1AL	290	32.3308	5.72	60.93	-0.813	65.82	WRKYGEK	$C-X_7-C-X_{24}-HTC$	C_2HC
III	TaWRKY5	Traes_1AL_B24F28600.1	1AL	284	30.9778	8.81	69.82	-0.576	52.69	WRKYGEK	$C-X_7-C-X_{23}-HSC$	C_2HC
III	TaWRKY9	Traes_1BL_46340D685.1	1BL	288	32.1716	5.59	60.66	-0.789	66.88	WRKYGEK	$C-X_7-C-X_{23}-HTC$	C_2HC
III	TaWRKY10	Traes_1BL_B4AFDB663.1	1BL	286	31.1086	6.51	60.49	-0.644	52.87	WRKYGEK	$C-X_7-C-X_{23}-HAC$	C_2HC
III	TaWRKY13	Traes_1BL_73811B853.1	1BL	150	UN	UN	45.07	-0.411	33.04	WRKYGQK	/	C_2HC
III	TaWRKY14	Traes_1DL_DFE1721E0.1	1DL	295	32.2249	7.81	57.93	-0.773	65.22	WRKYGEK	/	C_2HC
III	TaWRKY24	Traes_2AS_6269D889E.1	2AS	324	UN	UN	63.86	-0.604	49.3	WRKYGQK	$C-X_7-C-X_{23}-HTC$	C_2HC
III	TaWRKY29	Traes_2BS_B65714572.1	2BS	293	31.1185	6.24	65.63	-0.438	45.88	WRKYGQK	$C-X_7-C-X_{23}-HTC$	C_2HC
III	TaWRKY40	Traes_2DS_AD8820C42.1	2DS	229	24.4868	10.73	74.28	-0.468	50.51	WRKYGQK	$C-X_7-C-X_{24}-HTC$	C_2HC
III	TaWRKY44	Traes_3AL_2297D6E18.1	3AL	265	29.4811	5.63	40.57	-0.941	63.51	WRKYGQK	/	C_2HC
III	TaWRKY46	TRAES3BF051200110CFD_t1	3BF	294	31.2618	8	54.52	-0.549	52.58	WRKYGQK	/	C_2HC
III	TaWRKY56	TRAES3BF081400030CFD_t1	3BF	221	25.0354	5.67	43.3	-0.853	54.02	WRKYGQK	/	C_2HC
III	TaWRKY57	TRAES3BF001300030CFD_t1	3BF	269	29.1865	6.8	63.61	-0.474	50.77	WRKYGQK	$C-X_7-C-X_{23}-HTC$	C_2HC
III	TaWRKY58	TRAES3BF005100010CFD_t1	3BF	272	29.2448	7.04	70.07	-0.357	56.09	WRKYGQK	$C-X_7-C-X_{24}-HTC$	C_2HC
III	TaWRKY59	TRAES3BF005100020CFD_t1	3BF	147	15.7607	8.93	71.16	-0.42	40.47	WRKYGQK	$C-X_7-C-X_{23}-HTC$	C_2HC
III	TaWRKY60	TRAES3BF005100030CFD_t1	3BF	351	37.7058	6.63	57.61	-0.606	44.74	WRKYGQK	$C-X_7-C-X_{23}-HTC$	C_2HC
III	TaWRKY64	Traes_3DL_48F7A19D2.1	3DL	143	37.7506	8.69	44.41	0.926	61.67	WRKYGQK	$C-X_7-C-X_{28}-HSC$	C_2HC
III	TaWRKY65	Traes_4AS_70DF607CC.1	4AS	206	26.2649	8.38	49.85	-0.767	63.02	WRKYGEK	$C-X_7-C-X_{23}-HTC$	C_2HC
III	TaWRKY66	Traes_4AL_9E0D1CFA6.1	4AL	222	37.7506	8.57	44.05	-0.824	54.93	WRKYGEK	$C-X_7-C-X_{28}-HSC$	C_2HC
III	TaWRKY70	Traes_4BS_A6D9EB0E5.1	4BL	278	38.6751	9.49	54.53	-0.726	61.17	WRKYGEK	/	C_2HC

III	TaWRKY72	Traes_4BL_A8C6FBEB6.1	4BL	206	21.0834	8.09	49.37	-0.793	64.94	WRKYGEK	C-X_7-C-X_{23}-HTC	C_2HC
III	TaWRKY74	Traes_4DS_FE38A59D0.1	4DS	222	33.0295	8.57	44.05	-0.824	54.93	WRKYGEK	C-X_7-C-X_{23}-HTC	C_2HC
III	TaWRKY76	Traes_4DL_3140A8240.1	4DL	206	43.6012	8.34	49.37	-0.762	63.4	WRKYGEK	C-X_7-C-X_{24}-HTC	C_2HC
III	TaWRKY78	Traes_5AL_B4E8A3115.2	5AL	1,482	10.3644	6.85	95.59	-0.175	42.96	WRKYGQK	/	C_2HC
III	TaWRKY84	Traes_5BL_0A3D332A8.1	5BL	339	UN	5.91	59.03	-0.5	57.86	WRKYGQK	C-X_7-C-X_{24}-HTC	C_2HC
III	TaWRKY85	Traes_5BL_175E7FC38.1	5BL	337	UN	7.59	53.38	-0.715	64.49	WRKYGQK	C-X_7-C-X_{23}-HTC	C_2HC
III	TaWRKY92	Traes_5DL_C93641E43.1	5DL	341	23.1771	5.91	59	-0.53	56	WRKYGQK	C-X_7-C-X_{24}-HTC	C_2HC
III	TaWRKY95	Traes_5DL_5C93510D5.1	5DL	351	26.317	5.82	63.73	-0.343	54.37	WRKYGQK	C-X_7-C-X_{24}-HTC	C_2HC
III	TaWRKY101	Traes_6AS_68775100B.1	6AL	189	26.2969	6.02	51.22	-0.376	46.92	WRKYGQK	C-X_6-C-X_{23}-HTC	C_2HC
III	TaWRKY110	Traes_7DS_01F74C6F3.1	7DS	90	26.423	9.13	53.22	-0.881	27.27	WRKYGQK	C-X_6-C-X_{23}-HTC	C_2HC
III	TaWRKY112	Traes_7DL_A9EF00572.1	7DL	226	18.7661	UN	61.95	-0.758	54.75	WRKYGQK	/	C_2HC
III	TaWRKY121	Traes_2AS_1AFFE8DA6.1	2AS_scaff_5294211	44	22.6461	10.1	39.77	-1.889	71.09	WRKYGQK	C-X_6-C-X_{23}-HTC	C_2HC
III	TaWRKY122	Traes_2DL_362A1F535.1	2DL_scaff_9707610	70	19.0303	9.42	34.86	-1.336	57.44	WRKYGQK	C-X_7-C-X_{24}-HTC	C_2HC
III	TaWRKY126	Traes_3AL_6E92D4E1F.1	3AL_scaff_2835273	80	30.3569	9.15	48.75	-0.99	24.33	WRKYGQK	C-X_7-C-X_{24}-HSC	C_2HC
III	TaWRKY132	Traes_3B_8B0D448D8.1	3B_scaff_10684507	255	7.8144	6.79	54.78	-0.603	50.07	WRKYGQK	C-X_7-C-X_{24}-HSC	C_2HC
III	TaWRKY134	Traes_3DL_678D51EAD.1	3DL_scaff_5358175	68	6.5586	10.02	41.62	-1.159	32.13	WRKYGQK	C-X_7-C-X_{24}-HSC	C_2HC
III	TaWRKY135	Traes_3DL_7456F61A3.1	3DL_scaff_5877113	53	11.2758	9.15	23.96	-1.262	25.95	WRKYGQK	C-X_7-C-X_{24}-HSC	C_2HC
III	TaWRKY136	Traes_3DL_2551BF2C1.1	3DL_scaff_6811598	52	7.2742	9.55	31.73	-0.906	36.72	WRKYGQK	C-X_7-C-X_{24}-HSC	C_2HC
III	TaWRKY137	Traes_3DS_9A02CF31D.1	3DS_scaff_2567572	303	16.9304	6.83	101.98	-0.12	45.29	WRKYGQK	C-X_7-C-X_{24}-HSC	C_2HC
III	TaWRKY142	Traes_4AS_0DA136E0E.1	4AS_scaff_5962726	135	37.6536	9.1	44.22	-0.841	54.23	/	C-X_7-C-X_{23}-HTC	C_2HC
III	TaWRKY146	Traes_5AL_69A969FF4.1	5AL_scaff_2698301	164	7.753	6.2	63.6	-0.988	64.22	WRKYGQK	C-X_7-C-X_{23}-HTC	C_2HC
III	TaWRKY147	Traes_5AL_6FDB440FB.1	5AL_scaff_2705439	69	9.1644	6.53	55.07	-0.761	42.81	/	C-X_7-C-X_{28}-HTC	C_2HC
III	TaWRKY153	Traes_5BL_F853EA802.1	5BL_scaff_10789552	346	17.5236	5.94	62.11	-0.381	47.7	WRKYGQK	C-X_7-C-X_{24}-HTC	C_2HC
III	TaWRKY160	Traes_5DL_09F1F8F79.1	5DL_scaff_4583222	334	16.2729	6.76	56.20	-0.657	58.31	WRKYGQK	C-X_7-C-X_{23}-HTC	C_2HC
III	TaWRKY169	Traes_7BL_53AA25AA1.1	7BL_scaff_6632473	55	UN	8.93	26.73	-1.538	44.13	WRKYGQK	C-X_7-C-X_{23}-HTC	C_2HC

Abbreviations: Chr, chromosome numbers; NA, number of amino acids; MW, molecular weight; p*I*, isoelectric point; Ai, Aliphatic index; GRAVY, grand average of hydropathicity; II, instability index; UN, undefined molecular weight and/or isoelectric point as sequence contains several consecutive undefined amino acids; "/", incomplete WRKY motif and/or zinc-finger structure.
