# Supplementary material for: Genome-wide analysis of WRKY transcription factors in wheat (Triticum aestivum L.) and differential expression under water deficit condition
Source: PeerJ. 2017 May 4;5:e3232. doi: 10.7717/peerj.3232 (PMC5420200; doi:10.7717/peerj.3232)
Supplement: Table S4 [file peerj-05-3232-s006.pdf]

Supplemental Table S4. Segmental and tandem duplication gene pairs identified in *TaWRKYs*.

Query id	Subject id	Identity(%)	Alignment length	Mismatches	Gap openings	Q. start	Q. end	S. start	S. end	E-value	Bit score
**Segmental duplication**											
TaWRKY48	*TaWRKY50*	100	1041	0	0	1	1041	1	1041	0	1923
TaWRKY68	*TaWRKY108*	100	41	0	0	1	41	1	41	6.04E-16	76.8
TaWRKY79	*TaWRKY98*	100	32	0	0	1	32	970	1001	1.46E-11	60.2
TaWRKY66	*TaWRKY74*	99.402	669	4	0	1	669	1	669	0	1214
TaWRKY109	*TaWRKY111*	99.02	918	9	0	1	918	1	918	0	1646
TaWRKY80	*TaWRKY91*	98.889	90	1	0	1	90	733	822	7.54E-42	161
TaWRKY6	*TaWRKY11*	98.876	178	2	0	231	408	27	204	4.62E-89	318
TaWRKY33	*TaWRKY39*	98.859	1578	15	1	1	1575	1	1578	0	2811
TaWRKY103	*TaWRKY107*	98.686	1446	19	0	1	1446	1	1446	0.00E+00	2566
TaWRKY82	*TaWRKY94*	98.502	1068	16	0	1	1068	427	1494	0	1884
TaWRKY65	*TaWRKY76*	98.39	621	10	0	1	621	1	621	0	1092
TaWRKY25	*TaWRKY41*	98.333	960	16	0	49	1008	160	1119	0	1685
TaWRKY11	*TaWRKY16*	98.315	178	3	0	27	204	231	408	1.03E-87	313
TaWRKY102	*TaWRKY106*	98.031	711	14	0	1	711	1	711	0	1236
TaWRKY21	*TaWRKY38*	97.971	690	14	0	1	690	1	690	0	1197
TaWRKY105	*TaWRKY106*	97.89	711	12	1	1	708	1	711	0	1227
TaWRKY81	*TaWRKY93*	97.852	1071	20	1	1	1068	1	1071	0	1847
TaWRKY69	*TaWRKY75*	97.823	827	15	1	1	824	343	1169	0	1424
TaWRKY23	*TaWRKY33*	97.655	1578	28	4	145	1716	1	1575	0	2700
TaWRKY25	*TaWRKY32*	97.619	1218	29	0	49	1266	190	1407	0	2089
TaWRKY66	*TaWRKY70*	97.608	669	16	0	1	669	169	837	0	1147
TaWRKY70	*TaWRKY74*	97.608	669	16	0	169	837	1	669	0	1147
TaWRKY88	*TaWRKY96*	97.496	1677	42	0	1	1677	1	1677	0	2865
TaWRKY102	*TaWRKY105*	97.468	711	15	1	1	711	1	708	0	1210
TaWRKY23	*TaWRKY39*	97.465	1578	34	2	145	1716	1	1578	0	2687
TaWRKY42	*TaWRKY63*	97.436	78	2	0	37	114	1	78	6.16E-34	134
TaWRKY53	*TaWRKY63*	97.436	273	7	0	331	603	1	273	2.33E-133	466
TaWRKY42	*TaWRKY53*	97.368	114	3	0	1	114	295	408	2.78E-52	195
TaWRKY72	*TaWRKY76*	97.262	621	17	0	1	621	1	621	0	1053
TaWRKY20	*TaWRKY31*	97.234	687	16	1	1	687	175	858	0	1160
TaWRKY29	*TaWRKY40*	97.086	549	11	2	1	546	139	685	0	922
TaWRKY24	*TaWRKY40*	96.869	543	15	1	1	543	139	679	0	909
TaWRKY4	*TaWRKY9*	96.804	876	16	4	1	873	1	867	0	1452
TaWRKY65	*TaWRKY72*	96.779	621	20	0	1	621	1	621	0	1037
TaWRKY77	*TaWRKY96*	96.673	1713	57	0	1	1713	1	1713	0	2848
TaWRKY83	*TaWRKY99*	96.617	1005	31	1	1	1002	1	1005	0	1664
TaWRKY34	*TaWRKY38*	96.522	690	24	0	1	690	1	690	0	1142
TaWRKY44	*TaWRKY64*	96.503	429	15	0	1	429	1	429	0	710
TaWRKY27	*TaWRKY37*	96.477	369	7	2	619	987	1	363	7.83E-175	604
TaWRKY43	*TaWRKY63*	96.465	198	7	0	43	240	76	273	4.40E-92	327
TaWRKY43	*TaWRKY53*	96.465	198	7	0	43	240	406	603	4.40E-92	327
TaWRKY77	*TaWRKY88*	96.441	1770	51	7	1	1770	1	1758	0	2909
TaWRKY6	*TaWRKY16*	96.333	409	13	2	1	408	1	408	0	671
TaWRKY24	*TaWRKY29*	96.25	560	18	1	1	560	1	557	0	915
TaWRKY21	*TaWRKY34*	96.232	690	26	0	1	690	1	690	0	1131
TaWRKY71	*TaWRKY73*	96.209	976	34	2	1	973	1	976	0	1594
TaWRKY44	*TaWRKY56*	95.84	649	15	1	150	798	30	666	0	1038
TaWRKY84	*TaWRKY92*	95.627	1029	33	3	1	1020	1	1026	0	1640
TaWRKY7	*TaWRKY15*	95.545	202	9	0	367	568	1	202	1.63E-90	324
TaWRKY90	*TaWRKY97*	95.462	595	23	2	324	918	10	600	0	946
TaWRKY4	*TaWRKY14*	95.119	881	32	3	1	873	11	888	0	1378
TaWRKY19	*TaWRKY28*	94.925	335	11	2	2	333	65	396	1.10E-149	520
TaWRKY32	*TaWRKY41*	94.778	1149	30	8	1	1149	1	1119	0	1762
TaWRKY26	*TaWRKY30*	94.723	2217	75	13	1	2202	1	2190	0	3428
TaWRKY28	*TaWRKY36*	94.646	579	22	4	43	615	1	576	0	889
TaWRKY5	*TaWRKY10*	94.366	852	45	2	1	852	1	849	0	1304
TaWRKY9	*TaWRKY14*	93.984	881	36	6	1	867	11	888	0	1317
TaWRKY67	*TaWRKY73*	93.021	1003	43	6	1	1003	1	976	0	1439
TaWRKY11	*TaWRKY36*	92.982	57	4	0	27	83	357	413	8.82E-19	84.2
TaWRKY56	*TaWRKY64*	92.857	280	8	1	30	297	150	429	3.43E-112	396
TaWRKY19	*TaWRKY36*	92.836	335	21	1	2	333	23	357	1.44E-138	483
TaWRKY34	*TaWRKY106*	92.337	261	20	0	373	633	403	663	6.00E-105	372
TaWRKY34	*TaWRKY105*	92.337	261	20	0	373	633	400	660	6.00E-105	372
TaWRKY67	*TaWRKY71*	92.231	1004	48	8	1	1004	1	974	0	1395
TaWRKY27	*TaWRKY35*	91.802	988	34	22	1	987	1	942	0	1332
TaWRKY21	*TaWRKY106*	91.76	267	19	3	368	633	399	663	7.76E-104	368
TaWRKY21	*TaWRKY105*	91.76	267	19	3	368	633	396	660	7.76E-104	368
TaWRKY34	*TaWRKY102*	91.571	261	22	0	373	633	403	663	1.30E-101	361
TaWRKY38	*TaWRKY106*	91.353	266	22	1	368	633	399	663	3.61E-102	363
TaWRKY38	*TaWRKY105*	91.353	266	22	1	368	633	396	660	3.61E-102	363
TaWRKY21	*TaWRKY102*	91.011	267	21	3	368	633	399	663	1.68E-100	357

TaWRKY1	*TaWRKY8*	90.725	1380	68	18	1	1323	1	1377	0	1784
TaWRKY38	*TaWRKY102*	90.602	266	24	1	368	633	399	663	7.81E-99	351
TaWRKY16	*TaWRKY28*	90.303	165	14	2	124	287	289	452	6.23E-58	671
TaWRKY6	*TaWRKY28*	90.244	164	16	0	124	287	289	452	6.23E-58	215
**Tandem duplication**											
TaWRKY59	*TaWRKY60*	99.551	445	1	1	1	444	1	445	0	809
TaWRKY113	*TaWRKY115*	91.734	496	35	5	4	494	1	495	0	684
TaWRKY113	*TaWRKY114*	92.371	367	26	2	31	396	1	366	4.82E-150	521
TaWRKY114	*TaWRKY115*	91.257	366	32	0	1	366	31	396	2.07E-143	499

This result was defined by Blastn, and the *TaWRKY* genes with identity(%)>90% are shown in the Table.
