# Supplementary material for: Genome-wide analysis of WRKY transcription factors in wheat (Triticum aestivum L.) and differential expression under water deficit condition
Source: PeerJ. 2017 May 4;5:e3232. doi: 10.7717/peerj.3232 (PMC5420200; doi:10.7717/peerj.3232)
Supplement: Table S5 [file peerj-05-3232-s007.pdf]

Supplementary Table S4. Putative *cis*-acting elements identified in the promoter regions of *TaWRKY* genes.

"*" means the *WRKY* gene contain this *cis*-acting element in the promoter region.

Transcript ID	Proposed name	Group	CAAT-box	TATA-box	A-box	OBP-1 site	ATGCAAAT motif	5UTR Py-rich stretch	AT-rich element	circadian	MSA-like	E2Fa
			common *cis*-acting element in promoter and enhancer regions	-30 of transcription start	*cis*-acting regulatory element	*cis*-acting regulatory element	*cis*-acting regulatory element associated to the GCN4 motif	high transcription levels	binding site of AT-rich DNA binding protein	circadian control	cell cycle regulation	E2F-binding site; G1-M transition of cell cycle
Traes_1AS_F3EAEC435.1	*TaWRKY1*	I	*	*	*					*		
Traes_1BS_EF67E5A24.1	*TaWRKY8*	I	*	*								
Traes_2AS_C407071E4.2	*TaWRKY23*	I	*	*						*		
Traes_2AS_0186B9E4F.2	*TaWRKY25*	I	*	*						*		
Traes_2AL_409AB7647.1	*TaWRKY26*	I	*	*	*			*		*		
Traes_2BL_2BA3A755A.1	*TaWRKY30*	I	*	*	*			*		*		
Traes_2BS_380EC4D1E.1	*TaWRKY32*	I	*	*	*			*			*	
Traes_2BS_D435A8999.1	*TaWRKY33*	I	*	*						*		
Traes_2DS_97E3E7CFC.1	*TaWRKY39*	I	*	*				*		*		
Traes_2DS_F6FBC974C.2	*TaWRKY41*	I	*	*	*							
Traes_4BL_EFEC50B26.2	*TaWRKY69*	I	*	*								
Traes_4DS_DC3C9DC42.2	*TaWRKY75*	I	*	*				*		*		
Traes_5AL_ED3ADED51.3	*TaWRKY77*	I	*	*						*		
Traes_5BS_C46781248.1	*TaWRKY82*	I	*	*	*	*		*				
Traes_5BL_A522C62D1.1	*TaWRKY83*	I	*	*			*			*		
Traes_5BL_17A712C94.1	*TaWRKY88*	I	*	*						*	*	
Traes_5DS_5DEA5C9E3.1	*TaWRKY94*	I	*	*		*						
Traes_5DL_21F7C6BF7.2	*TaWRKY96*	I	*	*				*			*	
Traes_5DL_46E3AC8D6.1	*TaWRKY98*	I	*	*				*		*		
Traes_5DL_4BA2CC560.2	*TaWRKY99*	I	*	*				*		*		
Traes_7BL_A46F1A830.2	*TaWRKY109*	I	*	*	*			*		*		
Traes_7DS_24C563960.1	*TaWRKY111*	I	*	*	*			*	*			
Traes_1DS_A6733B734.1	*TaWRKY120*	I	*	*	*				*	*		
Traes_3B_990298FF5.1	*TaWRKY133*	I	*	*	*		*	*	*	*		
Traes_4AL_2EEECCC4B.1	*TaWRKY141*	I	*	*	*					*		
Traes_5AL_E644A6A0B.1	*TaWRKY145*	I	*	*				*		*		
Traes_5BL_D3C383CF5.1	*TaWRKY152*	I	*	*				*		*		
Traes_7AL_48C81DE03.1	*TaWRKY168*	I	*	*								
Traes_7AL_48C81DE031.1	*TaWRKY171*	I	*	*								
Traes_5BL_8BEF7F9CD.1	*TaWRKY86*	IIa	*	*				*				
Traes_5BL_90757F0CC.1	*TaWRKY87*	IIa	*	*						*		
Traes_7DL_5968FA56C.1	*TaWRKY113*	IIa	*	*	*					*		
Traes_7DL_B09854286.1	*TaWRKY114*	IIa	*	*	*			*				
Traes_7DL_F849918EA.2	*TaWRKY115*	IIa	*	*						*		
Traes_6AS_DA75BB1FD.1	*TaWRKY161*	IIa	*	*	*			*	*			
TRAES3BF003800010CFD_t1	*TaWRKY47*	IIb	*	*	*		*	*				
Traes_4AL_3E11167D9.1	*TaWRKY68*	IIb	*	*	*					*		
Traes_6AL_0C0899C15.1	*TaWRKY104*	IIb	*	*	*					*		
Traes_7AS_C9DF68E53.2	*TaWRKY108*	IIb	*	*	*					*		
Traes_6DL_AB95B0CE0.1	*TaWRKY164*	IIb	*	*	*					*		
Traes_1AS_1432A2F79.1	*TaWRKY2*	IIc	*	*								
Traes_1AL_0404BC790.1	*TaWRKY6*	IIc	*	*					*	*		
Traes_1AL_9ADA7A031.1	*TaWRKY7*	IIc	*	*								
Traes_1BL_1D865A8CC.1	*TaWRKY11*	IIc	*	*	*			*		*		
Traes_1BL_9AFA4B870.1	*TaWRKY12*	IIc	*	*	*							
Traes_1DL_D550418641.2	*TaWRKY15*	IIc	*	*				*				
Traes_1DL_5BAB0B6BC.1	*TaWRKY16*	IIc	*	*						*		
Traes_1DL_D1EC7DEA6.1	*TaWRKY17*	IIc	*	*						*		
Traes_1DL_46428511F.1	*TaWRKY18*	IIc	*	*						*		
Traes_2AS_D0C21ADB5.1	*TaWRKY19*	IIc	*	*								
Traes_2AL_1B43EA59E.1	*TaWRKY20*	IIc	*	*			*			*		
Traes_2AL_B1270662B.1	*TaWRKY21*	IIc	*	*	*					*		
Traes_2BS_F3097F116.1	*TaWRKY28*	IIc	*	*				*		*		
Traes_2BL_A69F6C5DF.1	*TaWRKY31*	IIc	*	*	*			*				
Traes_2BL_A5BFA97B9.1	*TaWRKY34*	IIc	*	*				*		*	*	
Traes_2DS_0F2500A60.1	*TaWRKY36*	IIc	*	*				*		*		
Traes_2DL_4F9F8F1F0.1	*TaWRKY38*	IIc	*	*	*			*	*	*		
Traes_3AL_67ECA2932.1	*TaWRKY42*	IIc	*	*	*					*		
Traes_3AL_1B73D2C12.1	*TaWRKY43*	IIc	*	*						*		
TRAES3BF066700160CFD_t1	*TaWRKY45*	IIc	*	*				*		*		
TRAES3BF021300010CFD_t1	*TaWRKY48*	IIc	*	*	*		*	*		*		
TRAES3BF267200010CFD_t1	*TaWRKY49*	IIc	*	*	*					*	*	
TRAES3BF021100090CFD_t1	*TaWRKY50*	IIc	*	*	*		*	*		*		
TRAES3BF058500060CFD_t1	*TaWRKY52*	IIc	*	*	*			*				
TRAES3BF111700140CFD_t1	*TaWRKY53*	IIc	*	*	*							
TRAES3BF090100100CFD_t1	*TaWRKY61*	IIc	*	*	*						*	
TRAES3BF045500040CFD_t1	*TaWRKY62*	IIc	*	*	*							

Traes_3DL_DF0D3F3FE.1	*TaWRKY63*	IIc	*	*				*		*		
Traes_5AL_E566BD64E.1	*TaWRKY79*	IIc	*	*								
Traes_5BL_C1D6B6B74.2	*TaWRKY90*	IIc	*	*	*			*		*		
Traes_5DL_7E2053226.2	*TaWRKY97*	IIc	*	*						*	*	
Traes_6AL_BA4636569.1	*TaWRKY102*	IIc	*	*	*			*		*		
Traes_6BL_DD840863A.1	*TaWRKY105*	IIc	*	*				*		*		
Traes_6DL_D29E210A1.1	*TaWRKY106*	IIc	*	*	*					*		
Traes_1AL_F64E07A92.1	*TaWRKY116*	IIc	*	*				*				
Traes_1BL_B15990028.1	*TaWRKY118*	IIc	*		*							
Traes_3AL_4769A72F1.1	*TaWRKY125*	IIc	*	*				*				
Traes_3AL_AB2BAE660.1	*TaWRKY129*	IIc	*	*	*			*		*		
Traes_3AL_140B829CB.2	*TaWRKY130*	IIc	*	*	*			*		*		
Traes_4AL_234E1CDF6.1	*TaWRKY140*	IIc	*	*				*				
Traes_4DS_CFC487CE5.2	*TaWRKY143*	IIc	*	*				*		*		
Traes_6DS_BF71C1557.2	*TaWRKY166*	IIc										
Traes_2AL_434E9F101.1	*TaWRKY22*	IId	*	*	*			*				
Traes_2AL_15A7BB684.1	*TaWRKY27*	IId	*	*				*		*		
Traes_2BL_6B75B32E3.1	*TaWRKY35*	IId	*	*	*		*		*	*		
Traes_2DL_F600B5FDF.1	*TaWRKY37*	IId	*	*								
Traes_4AL_98B1C762B.1	*TaWRKY67*	IId	*	*						*		
Traes_4BS_CE839571B.2	*TaWRKY71*	IId	*	*	*			*			*	
Traes_4DS_3BE557D5C.2	*TaWRKY73*	IId	*	*	*			*				
Traes_5AL_6F7D1D441.1	*TaWRKY80*	IId	*	*						*		
Traes_5BS_E0345D5DF.2	*TaWRKY81*	IId	*	*	*			*				
Traes_5BL_8688F70C9.1	*TaWRKY89*	IId	*	*	*		*			*		
Traes_5BL_E294922A9.2	*TaWRKY91*	IId	*	*	*			*		*		
Traes_5DS_D83DEA9B0.1	*TaWRKY93*	IId	*	*	*			*		*		
Traes_5BL_AEF9FE805.2	*TaWRKY154*	IId	*		*							
Traes_1AL_4E924201A.1	*TaWRKY3*	IIe	*	*								
TRAES3BF180700010CFD_t1	*TaWRKY51*	IIe	*	*	*					*		
TRAES3BF073300120CFD_t1	*TaWRKY54*	IIe	*	*	*					*		
TRAES3BF029000080CFD_t1	*TaWRKY55*	IIe	*	*	*							
Traes_5DL_E4A6D1889.2	*TaWRKY100*	IIe	*	*				*		*		
Traes_6AL_A5FB7CFA5.1	*TaWRKY103*	IIe	*	*	*	*				*		
Traes_6DL_D4F2CDDDC.1	*TaWRKY107*	IIe	*	*	*					*		*
Traes_1BL_794E99FF5.1	*TaWRKY119*	IIe	*	*						*		
Traes_5BL_B9DD3E76F.1	*TaWRKY155*	IIe	*	*				*		*		
Traes_1AL_309623B48.1	*TaWRKY4*	III	*	*						*		
Traes_1AL_B24F28600.1	*TaWRKY5*	III	*	*			*					
Traes_1BL_46340D685.1	*TaWRKY9*	III	*	*				*				
Traes_1BL_B4AFDB663.1	*TaWRKY10*	III	*	*								
Traes_1BL_73811B853.1	*TaWRKY13*	III	*	*						*		
Traes_1DL_DFE1721E0.1	*TaWRKY14*	III	*	*			*	*				
Traes_2AS_6269D889E.1	*TaWRKY24*	III	*	*	*					*		
Traes_2BS_B65714572.1	*TaWRKY29*	III	*	*						*		*
Traes_2DS_AD8820C42.1	*TaWRKY40*	III	*	*				*		*		
Traes_3AL_2297D6E18.1	*TaWRKY44*	III	*	*						*		
TRAES3BF051200110CFD_t1	*TaWRKY46*	III	*	*	*							*
TRAES3BF081400030CFD_t1	*TaWRKY56*	III	*	*						*	*	
TRAES3BF001300030CFD_t1	*TaWRKY57*	III	*	*						*		
TRAES3BF005100010CFD_t1	*TaWRKY58*	III	*	*	*					*		
TRAES3BF005100020CFD_t1	*TaWRKY59*	III	*	*						*		
TRAES3BF005100030CFD_t1	*TaWRKY60*	III	*	*						*		
Traes_3DL_48F7A19D2.1	*TaWRKY64*	III	*	*	*							
Traes_4AS_70DF607CC.1	*TaWRKY65*	III	*	*				*		*	*	
Traes_4AL_9E0D1CFA6.1	*TaWRKY66*	III	*	*				*	*	*		
Traes_4BS_A6D9EB0E5.1	*TaWRKY70*	III	*	*		*				*		
Traes_4BL_A8C6FBEB6.1	*TaWRKY72*	III	*	*				*		*		
Traes_4DS_FE38A59D0.1	*TaWRKY74*	III	*	*						*		
Traes_4DL_3140A8240.1	*TaWRKY76*	III	*	*				*		*		
Traes_5AL_B4E8A3115.2	*TaWRKY78*	III	*	*								
Traes_5BL_0A3D332A8.1	*TaWRKY84*	III	*	*						*		
Traes_5BL_175E7FC38.1	*TaWRKY85*	III	*	*						*		*
Traes_5DL_C93641E43.1	*TaWRKY92*	III	*	*	*							*
Traes_5DL_5C93510D5.1	*TaWRKY95*	III	*	*	*					*		*
Traes_6AS_68775100B.1	*TaWRKY101*	III	*	*	*					*		
Traes_7DS_01F74C6F3.1	*TaWRKY110*	III	*	*	*			*				
Traes_7DL_A9EF00572.1	*TaWRKY112*	III	*	*						*		
Traes_2AS_1AFFE8DA6.1	*TaWRKY121*	III	*	*	*							
Traes_2DL_362A1F535.1	*TaWRKY122*	III	*	*								
Traes_3B_8B0D448D8.1	*TaWRKY132*	III	*	*			*	*				
Traes_3DL_2551BF2C1.1	*TaWRKY136*	III										
Traes_4AS_0DA136E0E.1	*TaWRKY142*	III	*	*	*	*				*		
Traes_5DL_09F1F8F79.1	*TaWRKY160*	III	*	*						*		*
Traes_7BL_53AA25AA1.1	*TaWRKY169*	III	*	*	*							

Transcript ID	Proposed name	Group	E2Fb	Skn-1_motif	GCN4_motif	CCGTCC-box	CAT-box	OCT	dOCT	NON-box	RY-element	HD-Zip 1	HD-Zip 2	as1	motif I
			E2F-binding site; G1-M transition of cell cycle	endosperm expression	endosperm expression	meristem specific activation	meristem expression	meristem specific activation	meristem specific activation	meristem specific activation	seed-specific regulation	differentiation of the palisade mesophyll cells	control of leaf morphology development	root-specific expression	root specific
Traes_1AS_F3EAEC435.1	*TaWRKY1*	I		*	*	*	*							*	
Traes_1BS_EF67E5A24.1	*TaWRKY8*	I		*			*								
Traes_2AS_C407071E4.2	*TaWRKY23*	I		*											
Traes_2AS_0186B9E4F.2	*TaWRKY25*	I		*	*		*				*				
Traes_2AL_409AB7647.1	*TaWRKY26*	I		*	*	*	*								
Traes_2BL_2BA3A755A.1	*TaWRKY30*	I		*		*									
Traes_2BS_380EC4D1E.1	*TaWRKY32*	I		*		*	*								
Traes_2BS_D435A8999.1	*TaWRKY33*	I		*	*										
Traes_2DS_97E3E7CFC.1	*TaWRKY39*	I		*	*						*				
Traes_2DS_F6FBC974C.2	*TaWRKY41*	I		*	*	*									
Traes_4BL_EFEC50B26.2	*TaWRKY69*	I		*			*								
Traes_4DS_DC3C9DC42.2	*TaWRKY75*	I		*											
Traes_5AL_ED3ADED51.3	*TaWRKY77*	I		*			*								
Traes_5BS_C46781248.1	*TaWRKY82*	I		*		*						*	*		
Traes_5BL_A522C62D1.1	*TaWRKY83*	I		*	*		*								
Traes_5BL_17A712C94.1	*TaWRKY88*	I		*			*								
Traes_5DS_5DEA5C9E3.1	*TaWRKY94*	I		*	*		*								
Traes_5DL_21F7C6BF7.2	*TaWRKY96*	I		*			*								
Traes_5DL_46E3AC8D6.1	*TaWRKY98*	I		*											
Traes_5DL_4BA2CC560.2	*TaWRKY99*	I		*							*				
Traes_7BL_A46F1A830.2	*TaWRKY109*	I		*	*	*					*				
Traes_7DS_24C563960.1	*TaWRKY111*	I		*	*	*									
Traes_1DS_A6733B734.1	*TaWRKY120*	I			*	*	*								
Traes_3B_990298FF5.1	*TaWRKY133*	I		*		*									
Traes_4AL_2EEECCC4B.1	*TaWRKY141*	I		*		*									
Traes_5AL_E644A6A0B.1	*TaWRKY145*	I		*							*				
Traes_5BL_D3C383CF5.1	*TaWRKY152*	I		*				*							
Traes_7AL_48C81DE03.1	*TaWRKY168*	I		*											
Traes_7AL_48C81DE031.1	*TaWRKY171*	I		*											
Traes_5BL_8BEF7F9CD.1	*TaWRKY86*	IIa		*			*								
Traes_5BL_90757F0CC.1	*TaWRKY87*	IIa		*											
Traes_7DL_5968FA56C.1	*TaWRKY113*	IIa		*											
Traes_7DL_B09854286.1	*TaWRKY114*	IIa													
Traes_7DL_F849918EA.2	*TaWRKY115*	IIa		*	*										
Traes_6AS_DA75BB1FD.1	*TaWRKY161*	IIa	*	*		*					*				
TRAES3BF003800010CFD_t1	*TaWRKY47*	IIb		*		*	*		*						
Traes_4AL_3E11167D9.1	*TaWRKY68*	IIb		*		*						*	*		
Traes_6AL_0C0899C15.1	*TaWRKY104*	IIb		*	*	*									
Traes_7AS_C9DF68E53.2	*TaWRKY108*	IIb		*		*	*								
Traes_6DL_AB95B0CE0.1	*TaWRKY164*	IIb		*	*	*									
Traes_1AS_1432A2F79.1	*TaWRKY2*	IIc		*	*		*								
Traes_1AL_0404BC790.1	*TaWRKY6*	IIc		*			*								
Traes_1AL_9ADA7A031.1	*TaWRKY7*	IIc					*								
Traes_1BL_1D865A8CC.1	*TaWRKY11*	IIc		*		*									
Traes_1BL_9AFA4B870.1	*TaWRKY12*	IIc		*		*				*					
Traes_1DL_D550418641.2	*TaWRKY15*	IIc		*	*										
Traes_1DL_5BAB0B6BC.1	*TaWRKY16*	IIc		*			*								
Traes_1DL_D1EC7DEA6.1	*TaWRKY17*	IIc		*											
Traes_1DL_46428511F.1	*TaWRKY18*	IIc		*											
Traes_2AS_D0C21ADB5.1	*TaWRKY19*	IIc		*	*		*								
Traes_2AL_1B43EA59E.1	*TaWRKY20*	IIc		*	*		*				*				
Traes_2AL_B1270662B.1	*TaWRKY21*	IIc	*	*		*									
Traes_2BS_F3097F116.1	*TaWRKY28*	IIc		*			*		*		*				
Traes_2BL_A69F6C5DF.1	*TaWRKY31*	IIc		*	*	*									
Traes_2BL_A5BFA97B9.1	*TaWRKY34*	IIc		*											
Traes_2DS_0F2500A60.1	*TaWRKY36*	IIc													
Traes_2DL_4F9F8F1F0.1	*TaWRKY38*	IIc		*		*	*								
Traes_3AL_67ECA2932.1	*TaWRKY42*	IIc		*	*	*	*								
Traes_3AL_1B73D2C12.1	*TaWRKY43*	IIc		*											
TRAES3BF066700160CFD_t1	*TaWRKY45*	IIc		*				*							
TRAES3BF021300010CFD_t1	*TaWRKY48*	IIc		*	*	*									
TRAES3BF267200010CFD_t1	*TaWRKY49*	IIc		*		*									
TRAES3BF021100090CFD_t1	*TaWRKY50*	IIc		*	*	*									
TRAES3BF058500060CFD_t1	*TaWRKY52*	IIc		*	*	*	*								*
TRAES3BF111700140CFD_t1	*TaWRKY53*	IIc		*	*	*									
TRAES3BF090100100CFD_t1	*TaWRKY61*	IIc		*	*	*									
TRAES3BF045500040CFD_t1	*TaWRKY62*	IIc		*	*	*					*				
Traes_3DL_DF0D3F3FE.1	*TaWRKY63*	IIc		*	*		*								
Traes_5AL_E566BD64E.1	*TaWRKY79*	IIc		*			*				*				

Traes_5BL_C1D6B6B74.2	*TaWRKY90*	IIc		*		*								
Traes_5DL_7E2053226.2	*TaWRKY97*	IIc		*	*									
Traes_6AL_BA4636569.1	*TaWRKY102*	IIc		*		*	*							
Traes_6BL_DD840863A.1	*TaWRKY105*	IIc		*										
Traes_6DL_D29E210A1.1	*TaWRKY106*	IIc		*		*	*							
Traes_1AL_F64E07A92.1	*TaWRKY116*	IIc		*								*	*	
Traes_1BL_B15990028.1	*TaWRKY118*	IIc		*		*								*
Traes_3AL_4769A72F1.1	*TaWRKY125*	IIc		*	*									
Traes_3AL_AB2BAE660.1	*TaWRKY129*	IIc		*	*	*								
Traes_3AL_140B829CB.2	*TaWRKY130*	IIc		*	*	*								
Traes_4AL_234E1CDF6.1	*TaWRKY140*	IIc												
Traes_4DS_CFC487CE5.2	*TaWRKY143*	IIc		*			*		*		*			
Traes_6DS_BF71C1557.2	*TaWRKY166*	IIc												
Traes_2AL_434E9F101.1	*TaWRKY22*	IId		*	*	*	*							
Traes_2AL_15A7BB684.1	*TaWRKY27*	IId		*										
Traes_2BL_6B75B32E3.1	*TaWRKY35*	IId		*	*	*								
Traes_2DL_F600B5FDF.1	*TaWRKY37*	IId		*	*		*							
Traes_4AL_98B1C762B.1	*TaWRKY67*	IId		*			*							
Traes_4BS_CE839571B.2	*TaWRKY71*	IId				*	*			*				
Traes_4DS_3BE557D5C.2	*TaWRKY73*	IId				*	*							
Traes_5AL_6F7D1D441.1	*TaWRKY80*	IId		*										
Traes_5BS_E0345D5DF.2	*TaWRKY81*	IId		*		*								
Traes_5BL_8688F70C9.1	*TaWRKY89*	IId		*		*	*							
Traes_5BL_E294922A9.2	*TaWRKY91*	IId		*		*								
Traes_5DS_D83DEA9B0.1	*TaWRKY93*	IId		*	*	*								
Traes_5BL_AEF9FE805.2	*TaWRKY154*	IId				*	*							
Traes_1AL_4E924201A.1	*TaWRKY3*	IIe		*	*		*							
TRAES3BF180700010CFD_t1	*TaWRKY51*	IIe		*		*	*				*			
TRAES3BF073300120CFD_t1	*TaWRKY54*	IIe		*	*	*								
TRAES3BF029000080CFD_t1	*TaWRKY55*	IIe			*	*								
Traes_5DL_E4A6D1889.2	*TaWRKY100*	IIe		*										
Traes_6AL_A5FB7CFA5.1	*TaWRKY103*	IIe		*		*	*				*			
Traes_6DL_D4F2CDDDC.1	*TaWRKY107*	IIe		*		*								
Traes_1BL_794E99FF5.1	*TaWRKY119*	IIe	*	*	*									
Traes_5BL_B9DD3E76F.1	*TaWRKY155*	IIe		*	*									
Traes_1AL_309623B48.1	*TaWRKY4*	III		*	*						*			
Traes_1AL_B24F28600.1	*TaWRKY5*	III		*	*		*							
Traes_1BL_46340D685.1	*TaWRKY9*	III		*	*		*				*			
Traes_1BL_B4AFDB663.1	*TaWRKY10*	III		*			*							
Traes_1BL_73811B853.1	*TaWRKY13*	III		*								*	*	
Traes_1DL_DFE1721E0.1	*TaWRKY14*	III		*	*		*				*			
Traes_2AS_6269D889E.1	*TaWRKY24*	III		*		*	*							
Traes_2BS_B65714572.1	*TaWRKY29*	III		*			*							
Traes_2DS_AD8820C42.1	*TaWRKY40*	III		*	*									
Traes_3AL_2297D6E18.1	*TaWRKY44*	III		*										
TRAES3BF051200110CFD_t1	*TaWRKY46*	III		*		*								
TRAES3BF081400030CFD_t1	*TaWRKY56*	III		*										
TRAES3BF001300030CFD_t1	*TaWRKY57*	III		*										
TRAES3BF005100010CFD_t1	*TaWRKY58*	III		*	*	*	*							
TRAES3BF005100020CFD_t1	*TaWRKY59*	III		*	*									
TRAES3BF005100030CFD_t1	*TaWRKY60*	III		*	*									
Traes_3DL_48F7A19D2.1	*TaWRKY64*	III		*	*	*								
Traes_4AS_70DF607CC.1	*TaWRKY65*	III		*	*		*							
Traes_4AL_9E0D1CFA6.1	*TaWRKY66*	III		*			*							
Traes_4BS_A6D9EB0E5.1	*TaWRKY70*	III		*	*									*
Traes_4BL_A8C6FBEB6.1	*TaWRKY72*	III		*	*									
Traes_4DS_FE38A59D0.1	*TaWRKY74*	III		*			*					*	*	*
Traes_4DL_3140A8240.1	*TaWRKY76*	III		*	*									
Traes_5AL_B4E8A3115.2	*TaWRKY78*	III		*			*							
Traes_5BL_0A3D332A8.1	*TaWRKY84*	III		*			*							
Traes_5BL_175E7FC38.1	*TaWRKY85*	III		*	*						*			
Traes_5DL_C93641E43.1	*TaWRKY92*	III		*	*	*	*	*						
Traes_5DL_5C93510D5.1	*TaWRKY95*	III		*		*	*							
Traes_6AS_68775100B.1	*TaWRKY101*	III		*		*								
Traes_7DS_01F74C6F3.1	*TaWRKY110*	III		*	*	*								
Traes_7DL_A9EF00572.1	*TaWRKY112*	III		*	*									
Traes_2AS_1AFFE8DA6.1	*TaWRKY121*	III		*	*	*	*				*			
Traes_2DL_362A1F535.1	*TaWRKY122*	III												
Traes_3B_8B0D448D8.1	*TaWRKY132*	III			*									
Traes_3DL_2551BF2C1.1	*TaWRKY136*	III												
Traes_4AS_0DA136E0E.1	*TaWRKY142*	III		*	*	*	*							
Traes_5DL_09F1F8F79.1	*TaWRKY160*	III		*							*			
Traes_7BL_53AA25AA1.1	*TaWRKY169*	III		*		*	*							

Transcript ID	Proposed name	Group	AC-I	AC-II	ABRE	motif IIb	CE3	CE1	GARE-motif	P-box	TATC-box	TCA-element
			negative regulation of phloem expression; restricting the vascular expression to the xylem	negative regulation of phloem expression; restricting the vascular expression to the xylem	abscisic acid responsive	abscisic acid responsive	ABA and VP1 responsiveness	associated to ABRE, involved in ABA responsiveness	gibberellin-responsive	gibberellin-responsive	gibberellin-responsive	salicylic acid responsiveness
Traes_1AS_F3EAEC435.1	*TaWRKY1*	I			*							*
Traes_1BS_EF67E5A24.1	*TaWRKY8*	I		*	*		*		*			*
Traes_2AS_C407071E4.2	*TaWRKY23*	I			*							*
Traes_2AS_0186B9E4F.2	*TaWRKY25*	I			*							
Traes_2AL_409AB7647.1	*TaWRKY26*	I		*	*		*		*			*
Traes_2BL_2BA3A755A.1	*TaWRKY30*	I		*	*							*
Traes_2BS_380EC4D1E.1	*TaWRKY32*	I			*				*			
Traes_2BS_D435A8999.1	*TaWRKY33*	I							*			*
Traes_2DS_97E3E7CFC.1	*TaWRKY39*	I							*			*
Traes_2DS_F6FBC974C.2	*TaWRKY41*	I			*				*		*	
Traes_4BL_EFEC50B26.2	*TaWRKY69*	I										
Traes_4DS_DC3C9DC42.2	*TaWRKY75*	I										*
Traes_5AL_ED3ADED51.3	*TaWRKY77*	I		*	*							*
Traes_5BS_C46781248.1	*TaWRKY82*	I										
Traes_5BL_A522C62D1.1	*TaWRKY83*	I							*			*
Traes_5BL_17A712C94.1	*TaWRKY88*	I								*		*
Traes_5DS_5DEA5C9E3.1	*TaWRKY94*	I			*							*
Traes_5DL_21F7C6BF7.2	*TaWRKY96*	I								*		
Traes_5DL_46E3AC8D6.1	*TaWRKY98*	I		*	*				*			*
Traes_5DL_4BA2CC560.2	*TaWRKY99*	I			*				*			*
Traes_7BL_A46F1A830.2	*TaWRKY109*	I			*					*		
Traes_7DS_24C563960.1	*TaWRKY111*	I			*	*						*
Traes_1DS_A6733B734.1	*TaWRKY120*	I			*							*
Traes_3B_990298FF5.1	*TaWRKY133*	I			*				*	*		
Traes_4AL_2EEECCC4B.1	*TaWRKY141*	I	*						*			
Traes_5AL_E644A6A0B.1	*TaWRKY145*	I							*			*
Traes_5BL_D3C383CF5.1	*TaWRKY152*	I		*	*	*			*		*	*
Traes_7AL_48C81DE03.1	*TaWRKY168*	I	*									
Traes_7AL_48C81DE031.1	*TaWRKY171*	I	*									
Traes_5BL_8BEF7F9CD.1	*TaWRKY86*	IIa			*		*		*			
Traes_5BL_90757F0CC.1	*TaWRKY87*	IIa							*			*
Traes_7DL_5968FA56C.1	*TaWRKY113*	IIa			*							*
Traes_7DL_B09854286.1	*TaWRKY114*	IIa			*							*
Traes_7DL_F849918EA.2	*TaWRKY115*	IIa			*							*
Traes_6AS_DA75BB1FD.1	*TaWRKY161*	IIa			*							
TRAES3BF003800010CFD_t1	*TaWRKY47*	IIb			*					*		
Traes_4AL_3E11167D9.1	*TaWRKY68*	IIb							*			
Traes_6AL_0C0899C15.1	*TaWRKY104*	IIb										*
Traes_7AS_C9DF68E53.2	*TaWRKY108*	IIb			*				*			
Traes_6DL_AB95B0CE0.1	*TaWRKY164*	IIb			*							
Traes_1AS_1432A2F79.1	*TaWRKY2*	IIc	*		*						*	*
Traes_1AL_0404BC790.1	*TaWRKY6*	IIc			*				*			
Traes_1AL_9ADA7A031.1	*TaWRKY7*	IIc			*						*	
Traes_1BL_1D865A8CC.1	*TaWRKY11*	IIc										*
Traes_1BL_9AFA4B870.1	*TaWRKY12*	IIc			*		*		*			
Traes_1DL_D550418641.2	*TaWRKY15*	IIc							*			*
Traes_1DL_5BAB0B6BC.1	*TaWRKY16*	IIc		*								*
Traes_1DL_D1EC7DEA6.1	*TaWRKY17*	IIc							*			
Traes_1DL_46428511F.1	*TaWRKY18*	IIc			*	*	*			*		*
Traes_2AS_D0C21ADB5.1	*TaWRKY19*	IIc			*				*	*		
Traes_2AL_1B43EA59E.1	*TaWRKY20*	IIc			*	*						
Traes_2AL_B1270662B.1	*TaWRKY21*	IIc			*				*	*		
Traes_2BS_F3097F116.1	*TaWRKY28*	IIc			*							
Traes_2BL_A69F6C5DF.1	*TaWRKY31*	IIc			*			*				
Traes_2BL_A5BFA97B9.1	*TaWRKY34*	IIc	*		*							*
Traes_2DS_0F2500A60.1	*TaWRKY36*	IIc			*				*	*		*
Traes_2DL_4F9F8F1F0.1	*TaWRKY38*	IIc	*									
Traes_3AL_67ECA2932.1	*TaWRKY42*	IIc			*							*
Traes_3AL_1B73D2C12.1	*TaWRKY43*	IIc								*		*
TRAES3BF066700160CFD_t1	*TaWRKY45*	IIc			*							
TRAES3BF021300010CFD_t1	*TaWRKY48*	IIc			*				*			
TRAES3BF267200010CFD_t1	*TaWRKY49*	IIc			*	*		*	*	*		
TRAES3BF021100090CFD_t1	*TaWRKY50*	IIc			*				*			
TRAES3BF058500060CFD_t1	*TaWRKY52*	IIc	*		*	*			*		*	
TRAES3BF111700140CFD_t1	*TaWRKY53*	IIc			*				*	*		
TRAES3BF090100100CFD_t1	*TaWRKY61*	IIc			*							*
TRAES3BF045500040CFD_t1	*TaWRKY62*	IIc		*	*							
Traes_3DL_DF0D3F3FE.1	*TaWRKY63*	IIc							*	*		
Traes_5AL_E566BD64E.1	*TaWRKY79*	IIc										

Traes_5BL_C1D6B6B74.2	*TaWRKY90*	IIc	*		*							*
Traes_5DL_7E2053226.2	*TaWRKY97*	IIc			*		*		*			
Traes_6AL_BA4636569.1	*TaWRKY102*	IIc			*		*		*			*
Traes_6BL_DD840863A.1	*TaWRKY105*	IIc			*							
Traes_6DL_D29E210A1.1	*TaWRKY106*	IIc										
Traes_1AL_F64E07A92.1	*TaWRKY116*	IIc	*		*	*						*
Traes_1BL_B15990028.1	*TaWRKY118*	IIc										
Traes_3AL_4769A72F1.1	*TaWRKY125*	IIc			*							
Traes_3AL_AB2BAE660.1	*TaWRKY129*	IIc			*			*		*		
Traes_3AL_140B829CB.2	*TaWRKY130*	IIc			*				*			
Traes_4AL_234E1CDF6.1	*TaWRKY140*	IIc										
Traes_4DS_CFC487CE5.2	*TaWRKY143*	IIc		*	*							*
Traes_6DS_BF71C1557.2	*TaWRKY166*	IIc										
Traes_2AL_434E9F101.1	*TaWRKY22*	IId		*	*					*		
Traes_2AL_15A7BB684.1	*TaWRKY27*	IId	*	*	*							*
Traes_2BL_6B75B32E3.1	*TaWRKY35*	IId		*	*				*			*
Traes_2DL_F600B5FDF.1	*TaWRKY37*	IId			*							
Traes_4AL_98B1C762B.1	*TaWRKY67*	IId	*		*				*	*		
Traes_4BS_CE839571B.2	*TaWRKY71*	IId			*							
Traes_4DS_3BE557D5C.2	*TaWRKY73*	IId			*	*						*
Traes_5AL_6F7D1D441.1	*TaWRKY80*	IId			*							
Traes_5BS_E0345D5DF.2	*TaWRKY81*	IId	*	*						*		
Traes_5BL_8688F70C9.1	*TaWRKY89*	IId	*	*	*	*						
Traes_5BL_E294922A9.2	*TaWRKY91*	IId		*	*				*			
Traes_5DS_D83DEA9B0.1	*TaWRKY93*	IId	*	*	*					*		
Traes_5BL_AEF9FE805.2	*TaWRKY154*	IId										
Traes_1AL_4E924201A.1	*TaWRKY3*	IIe										*
TRAES3BF180700010CFD_t1	*TaWRKY51*	IIe				*				*		
TRAES3BF073300120CFD_t1	*TaWRKY54*	IIe	*		*			*				*
TRAES3BF029000080CFD_t1	*TaWRKY55*	IIe		*	*							
Traes_5DL_E4A6D1889.2	*TaWRKY100*	IIe		*						*		
Traes_6AL_A5FB7CFA5.1	*TaWRKY103*	IIe							*			
Traes_6DL_D4F2CDDDC.1	*TaWRKY107*	IIe		*	*							*
Traes_1BL_794E99FF5.1	*TaWRKY119*	IIe		*					*	*	*	
Traes_5BL_B9DD3E76F.1	*TaWRKY155*	IIe		*						*		
Traes_1AL_309623B48.1	*TaWRKY4*	III			*				*			*
Traes_1AL_B24F28600.1	*TaWRKY5*	III			*				*			
Traes_1BL_46340D685.1	*TaWRKY9*	III			*					*		
Traes_1BL_B4AFDB663.1	*TaWRKY10*	III							*			*
Traes_1BL_73811B853.1	*TaWRKY13*	III	*						*			*
Traes_1DL_DFE1721E0.1	*TaWRKY14*	III			*					*		*
Traes_2AS_6269D889E.1	*TaWRKY24*	III		*								
Traes_2BS_B65714572.1	*TaWRKY29*	III			*				*		*	*
Traes_2DS_AD8820C42.1	*TaWRKY40*	III		*					*			*
Traes_3AL_2297D6E18.1	*TaWRKY44*	III			*							*
TRAES3BF051200110CFD_t1	*TaWRKY46*	III	*									*
TRAES3BF081400030CFD_t1	*TaWRKY56*	III			*							*
TRAES3BF001300030CFD_t1	*TaWRKY57*	III			*				*	*		
TRAES3BF005100010CFD_t1	*TaWRKY58*	III			*							
TRAES3BF005100020CFD_t1	*TaWRKY59*	III							*			*
TRAES3BF005100030CFD_t1	*TaWRKY60*	III							*			*
Traes_3DL_48F7A19D2.1	*TaWRKY64*	III			*					*		*
Traes_4AS_70DF607CC.1	*TaWRKY65*	III			*				*			*
Traes_4AL_9E0D1CFA6.1	*TaWRKY66*	III										*
Traes_4BS_A6D9EB0E5.1	*TaWRKY70*	III										*
Traes_4BL_A8C6FBEB6.1	*TaWRKY72*	III			*				*			*
Traes_4DS_FE38A59D0.1	*TaWRKY74*	III										*
Traes_4DL_3140A8240.1	*TaWRKY76*	III				*						*
Traes_5AL_B4E8A3115.2	*TaWRKY78*	III			*				*			
Traes_5BL_0A3D332A8.1	*TaWRKY84*	III			*							
Traes_5BL_175E7FC38.1	*TaWRKY85*	III			*							
Traes_5DL_C93641E43.1	*TaWRKY92*	III			*							*
Traes_5DL_5C93510D5.1	*TaWRKY95*	III			*	*				*		
Traes_6AS_68775100B.1	*TaWRKY101*	III			*	*			*			
Traes_7DS_01F74C6F3.1	*TaWRKY110*	III			*				*			
Traes_7DL_A9EF00572.1	*TaWRKY112*	III			*							*
Traes_2AS_1AFFE8DA6.1	*TaWRKY121*	III			*							
Traes_2DL_362A1F535.1	*TaWRKY122*	III										
Traes_3B_8B0D448D8.1	*TaWRKY132*	III			*		*			*	*	
Traes_3DL_2551BF2C1.1	*TaWRKY136*	III										
Traes_4AS_0DA136E0E.1	*TaWRKY142*	III			*	*			*	*		*
Traes_5DL_09F1F8F79.1	*TaWRKY160*	III			*				*			
Traes_7BL_53AA25AA1.1	*TaWRKY169*	III			*				*			

Transcript ID	Proposed name	Group	SARE	TGA-element	AuxRR-core	TGA-box	AuxRE	O2-site	ERE	CGTCA-motif	TGACG-motif	TC-rich repeats
			salicylic acid responsiveness	auxin-responsive	auxin responsiveness	part of an auxin-responsive element	Auxin-responsive element	zein metabolism regulation	ethylene-responsive element	MeJA-responsiveness	MeJA-responsiveness	defense and stress responsiveness
Traes_1AS_F3EAEC435.1	*TaWRKY1*	I		*				*		*	*	
Traes_1BS_EF67E5A24.1	*TaWRKY8*	I								*	*	
Traes_2AS_C407071E4.2	*TaWRKY23*	I						*				
Traes_2AS_0186B9E4F.2	*TaWRKY25*	I		*								*
Traes_2AL_409AB7647.1	*TaWRKY26*	I								*	*	*
Traes_2BL_2BA3A755A.1	*TaWRKY30*	I		*						*	*	
Traes_2BS_380EC4D1E.1	*TaWRKY32*	I		*				*		*	*	
Traes_2BS_D435A8999.1	*TaWRKY33*	I						*				
Traes_2DS_97E3E7CFC.1	*TaWRKY39*	I			*				*			
Traes_2DS_F6FBC974C.2	*TaWRKY41*	I								*	*	
Traes_4BL_EFEC50B26.2	*TaWRKY69*	I								*	*	*
Traes_4DS_DC3C9DC42.2	*TaWRKY75*	I						*		*	*	*
Traes_5AL_ED3ADED51.3	*TaWRKY77*	I						*		*	*	*
Traes_5BS_C46781248.1	*TaWRKY82*	I						*		*	*	
Traes_5BL_A522C62D1.1	*TaWRKY83*	I						*		*	*	*
Traes_5BL_17A712C94.1	*TaWRKY88*	I			*			*		*	*	*
Traes_5DS_5DEA5C9E3.1	*TaWRKY94*	I								*	*	*
Traes_5DL_21F7C6BF7.2	*TaWRKY96*	I			*			*		*	*	*
Traes_5DL_46E3AC8D6.1	*TaWRKY98*	I								*	*	*
Traes_5DL_4BA2CC560.2	*TaWRKY99*	I						*		*	*	
Traes_7BL_A46F1A830.2	*TaWRKY109*	I					*	*		*	*	
Traes_7DS_24C563960.1	*TaWRKY111*	I		*						*	*	*
Traes_1DS_A6733B734.1	*TaWRKY120*	I						*		*	*	
Traes_3B_990298FF5.1	*TaWRKY133*	I						*				
Traes_4AL_2EEECCC4B.1	*TaWRKY141*	I		*						*	*	
Traes_5AL_E644A6A0B.1	*TaWRKY145*	I						*		*	*	
Traes_5BL_D3C383CF5.1	*TaWRKY152*	I								*	*	*
Traes_7AL_48C81DE03.1	*TaWRKY168*	I								*	*	
Traes_7AL_48C81DE031.1	*TaWRKY171*	I								*	*	
Traes_5BL_8BEF7F9CD.1	*TaWRKY86*	IIa		*	*					*	*	*
Traes_5BL_90757F0CC.1	*TaWRKY87*	IIa								*	*	*
Traes_7DL_5968FA56C.1	*TaWRKY113*	IIa		*						*	*	
Traes_7DL_B09854286.1	*TaWRKY114*	IIa		*				*				
Traes_7DL_F849918EA.2	*TaWRKY115*	IIa						*		*	*	
Traes_6AS_DA75BB1FD.1	*TaWRKY161*	IIa						*		*	*	
TRAES3BF003800010CFD_t1	*TaWRKY47*	IIb								*	*	*
Traes_4AL_3E11167D9.1	*TaWRKY68*	IIb						*		*	*	*
Traes_6AL_0C0899C15.1	*TaWRKY104*	IIb						*				*
Traes_7AS_C9DF68E53.2	*TaWRKY108*	IIb		*						*	*	*
Traes_6DL_AB95B0CE0.1	*TaWRKY164*	IIb		*	*			*		*	*	*
Traes_1AS_1432A2F79.1	*TaWRKY2*	IIc						*		*	*	*
Traes_1AL_0404BC790.1	*TaWRKY6*	IIc										
Traes_1AL_9ADA7A031.1	*TaWRKY7*	IIc			*							
Traes_1BL_1D865A8CC.1	*TaWRKY11*	IIc								*	*	*
Traes_1BL_9AFA4B870.1	*TaWRKY12*	IIc				*				*	*	*
Traes_1DL_D550418641.2	*TaWRKY15*	IIc						*				*
Traes_1DL_5BAB0B6BC.1	*TaWRKY16*	IIc						*				*
Traes_1DL_D1EC7DEA6.1	*TaWRKY17*	IIc						*	*			*
Traes_1DL_46428511F.1	*TaWRKY18*	IIc						*		*	*	*
Traes_2AS_D0C21ADB5.1	*TaWRKY19*	IIc								*	*	*
Traes_2AL_1B43EA59E.1	*TaWRKY20*	IIc								*	*	
Traes_2AL_B1270662B.1	*TaWRKY21*	IIc								*	*	*
Traes_2BS_F3097F116.1	*TaWRKY28*	IIc						*	*	*	*	
Traes_2BL_A69F6C5DF.1	*TaWRKY31*	IIc								*	*	
Traes_2BL_A5BFA97B9.1	*TaWRKY34*	IIc		*	*					*	*	
Traes_2DS_0F2500A60.1	*TaWRKY36*	IIc		*					*	*	*	*
Traes_2DL_4F9F8F1F0.1	*TaWRKY38*	IIc		*						*	*	*
Traes_3AL_67ECA2932.1	*TaWRKY42*	IIc								*	*	*
Traes_3AL_1B73D2C12.1	*TaWRKY43*	IIc		*				*		*	*	
TRAES3BF066700160CFD_t1	*TaWRKY45*	IIc		*						*	*	
TRAES3BF021300010CFD_t1	*TaWRKY48*	IIc								*	*	
TRAES3BF267200010CFD_t1	*TaWRKY49*	IIc		*				*		*	*	
TRAES3BF021100090CFD_t1	*TaWRKY50*	IIc						*		*	*	
TRAES3BF058500060CFD_t1	*TaWRKY52*	IIc						*		*	*	
TRAES3BF111700140CFD_t1	*TaWRKY53*	IIc		*						*	*	
TRAES3BF090100100CFD_t1	*TaWRKY61*	IIc								*	*	*
TRAES3BF045500040CFD_t1	*TaWRKY62*	IIc						*		*	*	
Traes_3DL_DF0D3F3FE.1	*TaWRKY63*	IIc						*				*
Traes_5AL_E566BD64E.1	*TaWRKY79*	IIc		*				*				
Traes_5BL_C1D6B6B74.2	*TaWRKY90*	IIc		*				*		*	*	*

Traes_5DL_7E2053226.2	*TaWRKY97*	IIc	*							*	*	
Traes_6AL_BA4636569.1	*TaWRKY102*	IIc		*						*	*	*
Traes_6BL_DD840863A.1	*TaWRKY105*	IIc			*			*		*	*	*
Traes_6DL_D29E210A1.1	*TaWRKY106*	IIc				*		*		*	*	*
Traes_1AL_F64E07A92.1	*TaWRKY116*	IIc			*							
Traes_1BL_B15990028.1	*TaWRKY118*	IIc		*				*		*	*	
Traes_3AL_4769A72F1.1	*TaWRKY125*	IIc										*
Traes_3AL_AB2BAE660.1	*TaWRKY129*	IIc		*				*		*	*	
Traes_3AL_140B829CB.2	*TaWRKY130*	IIc								*	*	
Traes_4AL_234E1CDF6.1	*TaWRKY140*	IIc										
Traes_4DS_CFC487CE5.2	*TaWRKY143*	IIc						*		*	*	
Traes_6DS_BF71C1557.2	*TaWRKY166*	IIc										
Traes_2AL_434E9F101.1	*TaWRKY22*	IId						*		*	*	
Traes_2AL_15A7BB684.1	*TaWRKY27*	IId		*						*	*	*
Traes_2BL_6B75B32E3.1	*TaWRKY35*	IId		*				*		*	*	
Traes_2DL_F600B5FDF.1	*TaWRKY37*	IId								*	*	
Traes_4AL_98B1C762B.1	*TaWRKY67*	IId			*			*		*	*	*
Traes_4BS_CE839571B.2	*TaWRKY71*	IId		*				*		*	*	*
Traes_4DS_3BE557D5C.2	*TaWRKY73*	IId							*	*	*	*
Traes_5AL_6F7D1D441.1	*TaWRKY80*	IId						*				
Traes_5BS_E0345D5DF.2	*TaWRKY81*	IId		*	*					*	*	
Traes_5BL_8688F70C9.1	*TaWRKY89*	IId						*		*	*	
Traes_5BL_E294922A9.2	*TaWRKY91*	IId			*				*	*	*	*
Traes_5DS_D83DEA9B0.1	*TaWRKY93*	IId		*				*	*	*	*	*
Traes_5BL_AEF9FE805.2	*TaWRKY154*	IId										*
Traes_1AL_4E924201A.1	*TaWRKY3*	IIe						*		*	*	*
TRAES3BF180700010CFD_t1	*TaWRKY51*	IIe						*	*	*	*	
TRAES3BF073300120CFD_t1	*TaWRKY54*	IIe						*		*	*	
TRAES3BF029000080CFD_t1	*TaWRKY55*	IIe										
Traes_5DL_E4A6D1889.2	*TaWRKY100*	IIe						*				*
Traes_6AL_A5FB7CFA5.1	*TaWRKY103*	IIe		*						*	*	
Traes_6DL_D4F2CDDDC.1	*TaWRKY107*	IIe			*	*				*	*	
Traes_1BL_794E99FF5.1	*TaWRKY119*	IIe	*							*	*	*
Traes_5BL_B9DD3E76F.1	*TaWRKY155*	IIe		*				*		*	*	*
Traes_1AL_309623B48.1	*TaWRKY4*	III		*	*			*		*	*	
Traes_1AL_B24F28600.1	*TaWRKY5*	III			*				*	*	*	*
Traes_1BL_46340D685.1	*TaWRKY9*	III		*				*				*
Traes_1BL_B4AFDB663.1	*TaWRKY10*	III						*		*	*	*
Traes_1BL_73811B853.1	*TaWRKY13*	III										*
Traes_1DL_DFE1721E0.1	*TaWRKY14*	III						*				
Traes_2AS_6269D889E.1	*TaWRKY24*	III								*	*	*
Traes_2BS_B65714572.1	*TaWRKY29*	III			*							
Traes_2DS_AD8820C42.1	*TaWRKY40*	III						*		*	*	*
Traes_3AL_2297D6E18.1	*TaWRKY44*	III		*				*		*	*	*
TRAES3BF051200110CFD_t1	*TaWRKY46*	III			*				*	*	*	*
TRAES3BF081400030CFD_t1	*TaWRKY56*	III			*			*	*	*	*	*
TRAES3BF001300030CFD_t1	*TaWRKY57*	III		*				*		*	*	*
TRAES3BF005100010CFD_t1	*TaWRKY58*	III			*					*	*	*
TRAES3BF005100020CFD_t1	*TaWRKY59*	III		*					*			*
TRAES3BF005100030CFD_t1	*TaWRKY60*	III		*					*			*
Traes_3DL_48F7A19D2.1	*TaWRKY64*	III		*				*		*	*	*
Traes_4AS_70DF607CC.1	*TaWRKY65*	III								*	*	
Traes_4AL_9E0D1CFA6.1	*TaWRKY66*	III		*	*			*		*	*	*
Traes_4BS_A6D9EB0E5.1	*TaWRKY70*	III						*		*	*	
Traes_4BL_A8C6FBEB6.1	*TaWRKY72*	III								*	*	
Traes_4DS_FE38A59D0.1	*TaWRKY74*	III						*		*	*	
Traes_4DL_3140A8240.1	*TaWRKY76*	III						*				
Traes_5AL_B4E8A3115.2	*TaWRKY78*	III			*			*		*	*	*
Traes_5BL_0A3D332A8.1	*TaWRKY84*	III						*	*	*	*	
Traes_5BL_175E7FC38.1	*TaWRKY85*	III								*	*	*
Traes_5DL_C93641E43.1	*TaWRKY92*	III						*		*	*	
Traes_5DL_5C93510D5.1	*TaWRKY95*	III				*	*			*	*	
Traes_6AS_68775100B.1	*TaWRKY101*	III		*						*	*	*
Traes_7DS_01F74C6F3.1	*TaWRKY110*	III						*	*			
Traes_7DL_A9EF00572.1	*TaWRKY112*	III							*			
Traes_2AS_1AFFE8DA6.1	*TaWRKY121*	III							*	*	*	
Traes_2DL_362A1F535.1	*TaWRKY122*	III										
Traes_3B_8B0D448D8.1	*TaWRKY132*	III								*	*	*
Traes_3DL_2551BF2C1.1	*TaWRKY136*	III										
Traes_4AS_0DA136E0E.1	*TaWRKY142*	III								*	*	*
Traes_5DL_09F1F8F79.1	*TaWRKY160*	III		*				*	*	*	*	
Traes_7BL_53AA25AA1.1	*TaWRKY169*	III						*		*	*	

Transcript ID	Proposed name	Group	ARE	GC-motif	ELI-box3	Box-W1	box E	EIRE	AT-rich sequence	W box	box S	GCC box	WUN-motif
			anaerobic induction	anoxic specific inducibility	elicitor-responsive element	fungal elicitor responsive	fungal elicitor	elicitor-responsive element	element for maximal elicitor-mediated activation	wounding and pathogen responsiveness (Binds WRKY type transcription factors)	wounding and pathogen responsiveness	wounding and pathogen responsiveness	wound-responsive
Traes_1AS_F3EAEC435.1	*TaWRKY1*	I	*	*		*				*			
Traes_1BS_EF67E5A24.1	*TaWRKY8*	I	*	*		*				*			
Traes_2AS_C407071E4.2	*TaWRKY23*	I	*	*		*				*	*		
Traes_2AS_0186B9E4F.2	*TaWRKY25*	I		*									
Traes_2AL_409AB7647.1	*TaWRKY26*	I		*					*				
Traes_2BL_2BA3A755A.1	*TaWRKY30*	I							*				
Traes_2BS_380EC4D1E.1	*TaWRKY32*	I	*	*					*		*	*	
Traes_2BS_D435A8999.1	*TaWRKY33*	I	*			*				*			
Traes_2DS_97E3E7CFC.1	*TaWRKY39*	I	*	*							*		
Traes_2DS_F6FBC974C.2	*TaWRKY41*	I						*			*		
Traes_4BL_EFEC50B26.2	*TaWRKY69*	I	*			*	*			*			
Traes_4DS_DC3C9DC42.2	*TaWRKY75*	I	*				*	*				*	
Traes_5AL_ED3ADED51.3	*TaWRKY77*	I	*	*		*	*			*	*		
Traes_5BS_C46781248.1	*TaWRKY82*	I	*					*					
Traes_5BL_A522C62D1.1	*TaWRKY83*	I	*	*		*				*		*	
Traes_5BL_17A712C94.1	*TaWRKY88*	I	*	*		*				*			
Traes_5DS_5DEA5C9E3.1	*TaWRKY94*	I	*	*		*				*			
Traes_5DL_21F7C6BF7.2	*TaWRKY96*	I	*	*		*			*	*	*		
Traes_5DL_46E3AC8D6.1	*TaWRKY98*	I	*			*				*	*		
Traes_5DL_4BA2CC560.2	*TaWRKY99*	I	*			*				*			
Traes_7BL_A46F1A830.2	*TaWRKY109*	I	*			*				*			
Traes_7DS_24C563960.1	*TaWRKY111*	I		*								*	
Traes_1DS_A6733B734.1	*TaWRKY120*	I	*			*				*			
Traes_3B_990298FF5.1	*TaWRKY133*	I	*	*		*				*			
Traes_4AL_2EEECCC4B.1	*TaWRKY141*	I	*			*				*		*	
Traes_5AL_E644A6A0B.1	*TaWRKY145*	I	*			*				*	*		
Traes_5BL_D3C383CF5.1	*TaWRKY152*	I	*	*		*				*			
Traes_7AL_48C81DE03.1	*TaWRKY168*	I											
Traes_7AL_48C81DE031.1	*TaWRKY171*	I											
Traes_5BL_8BEF7F9CD.1	*TaWRKY86*	IIa	*	*									
Traes_5BL_90757F0CC.1	*TaWRKY87*	IIa		*							*		
Traes_7DL_5968FA56C.1	*TaWRKY113*	IIa				*				*	*		
Traes_7DL_B09854286.1	*TaWRKY114*	IIa	*			*				*		*	
Traes_7DL_F849918EA.2	*TaWRKY115*	IIa		*				*					
Traes_6AS_DA75BB1FD.1	*TaWRKY161*	IIa		*		*				*			
TRAES3BF003800010CFD_t1	*TaWRKY47*	IIb	*	*		*				*			*
Traes_4AL_3E11167D9.1	*TaWRKY68*	IIb	*			*				*			
Traes_6AL_0C0899C15.1	*TaWRKY104*	IIb	*			*			*	*			
Traes_7AS_C9DF68E53.2	*TaWRKY108*	IIb		*		*				*	*		
Traes_6DL_AB95B0CE0.1	*TaWRKY164*	IIb	*			*			*	*			
Traes_1AS_1432A2F79.1	*TaWRKY2*	IIc	*	*								*	
Traes_1AL_0404BC790.1	*TaWRKY6*	IIc						*				*	
Traes_1AL_9ADA7A031.1	*TaWRKY7*	IIc	*			*				*			
Traes_1BL_1D865A8CC.1	*TaWRKY11*	IIc				*				*			
Traes_1BL_9AFA4B870.1	*TaWRKY12*	IIc		*		*				*			
Traes_1DL_D550418641.2	*TaWRKY15*	IIc	*										
Traes_1DL_5BAB0B6BC.1	*TaWRKY16*	IIc	*								*		
Traes_1DL_D1EC7DEA6.1	*TaWRKY17*	IIc											
Traes_1DL_46428511F.1	*TaWRKY18*	IIc		*									
Traes_2AS_D0C21ADB5.1	*TaWRKY19*	IIc	*			*				*	*		
Traes_2AL_1B43EA59E.1	*TaWRKY20*	IIc	*	*								*	
Traes_2AL_B1270662B.1	*TaWRKY21*	IIc	*	*									
Traes_2BS_F3097F116.1	*TaWRKY28*	IIc	*			*				*		*	
Traes_2BL_A69F6C5DF.1	*TaWRKY31*	IIc	*	*				*			*		
Traes_2BL_A5BFA97B9.1	*TaWRKY34*	IIc		*									
Traes_2DS_0F2500A60.1	*TaWRKY36*	IIc											
Traes_2DL_4F9F8F1F0.1	*TaWRKY38*	IIc	*	*									
Traes_3AL_67ECA2932.1	*TaWRKY42*	IIc	*										
Traes_3AL_1B73D2C12.1	*TaWRKY43*	IIc				*				*			
TRAES3BF066700160CFD_t1	*TaWRKY45*	IIc	*	*		*				*			
TRAES3BF021300010CFD_t1	*TaWRKY48*	IIc	*	*		*				*			
TRAES3BF267200010CFD_t1	*TaWRKY49*	IIc	*	*								*	
TRAES3BF021100090CFD_t1	*TaWRKY50*	IIc	*	*		*				*			
TRAES3BF058500060CFD_t1	*TaWRKY52*	IIc	*			*				*	*		
TRAES3BF111700140CFD_t1	*TaWRKY53*	IIc		*									
TRAES3BF090100100CFD_t1	*TaWRKY61*	IIc	*	*							*	*	
TRAES3BF045500040CFD_t1	*TaWRKY62*	IIc	*	*								*	
Traes_3DL_DF0D3F3FE.1	*TaWRKY63*	IIc	*	*									
Traes_5AL_E566BD64E.1	*TaWRKY79*	IIc				*				*			
Traes_5BL_C1D6B6B74.2	*TaWRKY90*	IIc		*		*	*			*			

Traes_5DL_7E2053226.2	*TaWRKY97*	IIc	*	*			*		*				
Traes_6AL_BA4636569.1	*TaWRKY102*	IIc	*					*				*	
Traes_6BL_DD840863A.1	*TaWRKY105*	IIc											*
Traes_6DL_D29E210A1.1	*TaWRKY106*	IIc		*							*		
Traes_1AL_F64E07A92.1	*TaWRKY116*	IIc	*	*									
Traes_1BL_B15990028.1	*TaWRKY118*	IIc						*					
Traes_3AL_4769A72F1.1	*TaWRKY125*	IIc	*			*	*			*			
Traes_3AL_AB2BAE660.1	*TaWRKY129*	IIc				*				*			
Traes_3AL_140B829CB.2	*TaWRKY130*	IIc	*	*		*				*			
Traes_4AL_234E1CDF6.1	*TaWRKY140*	IIc	*										
Traes_4DS_CFC487CE5.2	*TaWRKY143*	IIc	*		*							*	
Traes_6DS_BF71C1557.2	*TaWRKY166*	IIc											
Traes_2AL_434E9F101.1	*TaWRKY22*	IId		*		*				*			
Traes_2AL_15A7BB684.1	*TaWRKY27*	IId		*							*		
Traes_2BL_6B75B32E3.1	*TaWRKY35*	IId						*					
Traes_2DL_F600B5FDF.1	*TaWRKY37*	IId	*			*		*		*	*		*
Traes_4AL_98B1C762B.1	*TaWRKY67*	IId	*			*				*			
Traes_4BS_CE839571B.2	*TaWRKY71*	IId		*		*				*			
Traes_4DS_3BE557D5C.2	*TaWRKY73*	IId	*	*		*			*	*			
Traes_5AL_6F7D1D441.1	*TaWRKY80*	IId	*	*	*	*				*		*	
Traes_5BS_E0345D5DF.2	*TaWRKY81*	IId	*	*		*				*		*	
Traes_5BL_8688F70C9.1	*TaWRKY89*	IId		*									
Traes_5BL_E294922A9.2	*TaWRKY91*	IId		*		*				*	*		*
Traes_5DS_D83DEA9B0.1	*TaWRKY93*	IId	*			*				*			
Traes_5BL_AEF9FE805.2	*TaWRKY154*	IId		*			*				*		
Traes_1AL_4E924201A.1	*TaWRKY3*	IIe	*	*		*				*		*	
TRAES3BF180700010CFD_t1	*TaWRKY51*	IIe	*					*					
TRAES3BF073300120CFD_t1	*TaWRKY54*	IIe	*	*									
TRAES3BF029000080CFD_t1	*TaWRKY55*	IIe	*	*		*		*		*		*	*
Traes_5DL_E4A6D1889.2	*TaWRKY100*	IIe				*				*			
Traes_6AL_A5FB7CFA5.1	*TaWRKY103*	IIe	*	*							*		
Traes_6DL_D4F2CDDDC.1	*TaWRKY107*	IIe				*	*			*			
Traes_1BL_794E99FF5.1	*TaWRKY119*	IIe				*				*			
Traes_5BL_B9DD3E76F.1	*TaWRKY155*	IIe	*			*				*			
Traes_1AL_309623B48.1	*TaWRKY4*	III	*		*	*	*			*	*		
Traes_1AL_B24F28600.1	*TaWRKY5*	III	*			*	*			*			
Traes_1BL_46340D685.1	*TaWRKY9*	III	*			*				*	*		
Traes_1BL_B4AFDB663.1	*TaWRKY10*	III		*			*					*	
Traes_1BL_73811B853.1	*TaWRKY13*	III		*									
Traes_1DL_DFE1721E0.1	*TaWRKY14*	III	*	*									
Traes_2AS_6269D889E.1	*TaWRKY24*	III	*	*		*	*			*			
Traes_2BS_B65714572.1	*TaWRKY29*	III	*			*	*			*			
Traes_2DS_AD8820C42.1	*TaWRKY40*	III											
Traes_3AL_2297D6E18.1	*TaWRKY44*	III		*							*	*	
TRAES3BF051200110CFD_t1	*TaWRKY46*	III	*	*		*				*			
TRAES3BF081400030CFD_t1	*TaWRKY56*	III	*			*				*			
TRAES3BF001300030CFD_t1	*TaWRKY57*	III	*			*				*			
TRAES3BF005100010CFD_t1	*TaWRKY58*	III	*	*									
TRAES3BF005100020CFD_t1	*TaWRKY59*	III	*										
TRAES3BF005100030CFD_t1	*TaWRKY60*	III	*										
Traes_3DL_48F7A19D2.1	*TaWRKY64*	III	*	*			*				*	*	
Traes_4AS_70DF607CC.1	*TaWRKY65*	III	*	*		*				*			
Traes_4AL_9E0D1CFA6.1	*TaWRKY66*	III	*			*				*	*		
Traes_4BS_A6D9EB0E5.1	*TaWRKY70*	III	*										
Traes_4BL_A8C6FBEB6.1	*TaWRKY72*	III	*			*				*			
Traes_4DS_FE38A59D0.1	*TaWRKY74*	III	*	*									
Traes_4DL_3140A8240.1	*TaWRKY76*	III	*			*		*		*		*	
Traes_5AL_B4E8A3115.2	*TaWRKY78*	III	*			*				*			
Traes_5BL_0A3D332A8.1	*TaWRKY84*	III	*	*		*				*			
Traes_5BL_175E7FC38.1	*TaWRKY85*	III	*										
Traes_5DL_C93641E43.1	*TaWRKY92*	III	*	*		*				*	*		
Traes_5DL_5C93510D5.1	*TaWRKY95*	III	*										
Traes_6AS_68775100B.1	*TaWRKY101*	III	*	*		*				*			
Traes_7DS_01F74C6F3.1	*TaWRKY110*	III	*			*				*	*		
Traes_7DL_A9EF00572.1	*TaWRKY112*	III	*										
Traes_2AS_1AFFE8DA6.1	*TaWRKY121*	III	*			*				*	*		
Traes_2DL_362A1F535.1	*TaWRKY122*	III											
Traes_3B_8B0D448D8.1	*TaWRKY132*	III				*				*			
Traes_3DL_2551BF2C1.1	*TaWRKY136*	III											
Traes_4AS_0DA136E0E.1	*TaWRKY142*	III	*			*				*		*	
Traes_5DL_09F1F8F79.1	*TaWRKY160*	III	*										
Traes_7BL_53AA25AA1.1	*TaWRKY169*	III		*									

Transcript ID	Proposed name	Group	JERE	LTR	C-repeat/DRE	HSE	MBS	CCAAT-box	MBSI	MRE	G-box
			jasmonate ethylene responsive element; wounding and pathogen responsiveness	low-temperature responsiveness	cold- and dehydration-responsiveness	heat stress responsiveness	MYB binding site involved in drought-inducibility	MYBHv1 binding site	MYB binding site involved in flavonoid biosynthetic genes regulation	MYB binding site involved in light responsiveness	light responsiveness
Traes_1AS_F3EAEC435.1	*TaWRKY1*	I				*	*				*
Traes_1BS_EF67E5A24.1	*TaWRKY8*	I		*		*	*				*
Traes_2AS_C407071E4.2	*TaWRKY23*	I	*	*		*		*			*
Traes_2AS_0186B9E4F.2	*TaWRKY25*	I				*	*				*
Traes_2AL_409AB7647.1	*TaWRKY26*	I					*				*
Traes_2BL_2BA3A755A.1	*TaWRKY30*	I		*							*
Traes_2BS_380EC4D1E.1	*TaWRKY32*	I	*	*			*	*			*
Traes_2BS_D435A8999.1	*TaWRKY33*	I					*				*
Traes_2DS_97E3E7CFC.1	*TaWRKY39*	I		*		*	*				*
Traes_2DS_F6FBC974C.2	*TaWRKY41*	I		*							*
Traes_4BL_EFEC50B26.2	*TaWRKY69*	I				*	*	*			*
Traes_4DS_DC3C9DC42.2	*TaWRKY75*	I		*				*			*
Traes_5AL_ED3ADED51.3	*TaWRKY77*	I		*		*		*			*
Traes_5BS_C46781248.1	*TaWRKY82*	I					*			*	*
Traes_5BL_A522C62D1.1	*TaWRKY83*	I		*		*	*			*	*
Traes_5BL_17A712C94.1	*TaWRKY88*	I					*	*		*	*
Traes_5DS_5DEA5C9E3.1	*TaWRKY94*	I			*	*	*				*
Traes_5DL_21F7C6BF7.2	*TaWRKY96*	I		*			*	*			
Traes_5DL_46E3AC8D6.1	*TaWRKY98*	I		*							*
Traes_5DL_4BA2CC560.2	*TaWRKY99*	I		*		*	*	*			*
Traes_7BL_A46F1A830.2	*TaWRKY109*	I		*			*				*
Traes_7DS_24C563960.1	*TaWRKY111*	I					*				*
Traes_1DS_A6733B734.1	*TaWRKY120*	I				*	*	*			*
Traes_3B_990298FF5.1	*TaWRKY133*	I					*				*
Traes_4AL_2EEECCC4B.1	*TaWRKY141*	I					*	*		*	*
Traes_5AL_E644A6A0B.1	*TaWRKY145*	I		*		*	*				
Traes_5BL_D3C383CF5.1	*TaWRKY152*	I		*			*				*
Traes_7AL_48C81DE03.1	*TaWRKY168*	I									
Traes_7AL_48C81DE031.1	*TaWRKY171*	I									
Traes_5BL_8BEF7F9CD.1	*TaWRKY86*	IIa		*			*				*
Traes_5BL_90757F0CC.1	*TaWRKY87*	IIa					*	*	*		*
Traes_7DL_5968FA56C.1	*TaWRKY113*	IIa		*		*	*				*
Traes_7DL_B09854286.1	*TaWRKY114*	IIa				*	*				*
Traes_7DL_F849918EA.2	*TaWRKY115*	IIa		*		*	*	*			*
Traes_6AS_DA75BB1FD.1	*TaWRKY161*	IIa		*		*	*				*
TRAES3BF003800010CFD_t1	*TaWRKY47*	IIb		*			*				*
Traes_4AL_3E11167D9.1	*TaWRKY68*	IIb					*				*
Traes_6AL_0C0899C15.1	*TaWRKY104*	IIb					*			*	*
Traes_7AS_C9DF68E53.2	*TaWRKY108*	IIb					*	*			*
Traes_6DL_AB95B0CE0.1	*TaWRKY164*	IIb					*			*	*
Traes_1AS_1432A2F79.1	*TaWRKY2*	IIc					*				
Traes_1AL_0404BC790.1	*TaWRKY6*	IIc		*						*	*
Traes_1AL_9ADA7A031.1	*TaWRKY7*	IIc					*				*
Traes_1BL_1D865A8CC.1	*TaWRKY11*	IIc				*	*				*
Traes_1BL_9AFA4B870.1	*TaWRKY12*	IIc		*			*	*			*
Traes_1DL_D550418641.2	*TaWRKY15*	IIc				*					*
Traes_1DL_5BAB0B6BC.1	*TaWRKY16*	IIc					*				*
Traes_1DL_D1EC7DEA6.1	*TaWRKY17*	IIc		*							*
Traes_1DL_46428511F.1	*TaWRKY18*	IIc						*		*	*
Traes_2AS_D0C21ADB5.1	*TaWRKY19*	IIc					*				*
Traes_2AL_1B43EA59E.1	*TaWRKY20*	IIc					*				*
Traes_2AL_B1270662B.1	*TaWRKY21*	IIc				*	*				*
Traes_2BS_F3097F116.1	*TaWRKY28*	IIc									*
Traes_2BL_A69F6C5DF.1	*TaWRKY31*	IIc						*			*
Traes_2BL_A5BFA97B9.1	*TaWRKY34*	IIc					*	*			*
Traes_2DS_0F2500A60.1	*TaWRKY36*	IIc				*					
Traes_2DL_4F9F8F1F0.1	*TaWRKY38*	IIc									*
Traes_3AL_67ECA2932.1	*TaWRKY42*	IIc		*			*				*
Traes_3AL_1B73D2C12.1	*TaWRKY43*	IIc				*	*				
TRAES3BF066700160CFD_t1	*TaWRKY45*	IIc		*			*	*			*
TRAES3BF021300010CFD_t1	*TaWRKY48*	IIc					*				*
TRAES3BF267200010CFD_t1	*TaWRKY49*	IIc					*				*
TRAES3BF021100090CFD_t1	*TaWRKY50*	IIc					*				*
TRAES3BF058500060CFD_t1	*TaWRKY52*	IIc					*				*
TRAES3BF111700140CFD_t1	*TaWRKY53*	IIc						*			*
TRAES3BF090100100CFD_t1	*TaWRKY61*	IIc		*			*	*			*
TRAES3BF045500040CFD_t1	*TaWRKY62*	IIc					*			*	*
Traes_3DL_DF0D3F3FE.1	*TaWRKY63*	IIc					*	*			*
Traes_5AL_E566BD64E.1	*TaWRKY79*	IIc		*			*				*

Traes_5BL_C1D6B6B74.2	*TaWRKY90*	IIc				*	*				*
Traes_5DL_7E2053226.2	*TaWRKY97*	IIc						*			*
Traes_6AL_BA4636569.1	*TaWRKY102*	IIc				*	*	*			*
Traes_6BL_DD840863A.1	*TaWRKY105*	IIc					*				*
Traes_6DL_D29E210A1.1	*TaWRKY106*	IIc						*			*
Traes_1AL_F64E07A92.1	*TaWRKY116*	IIc		*		*	*				*
Traes_1BL_B15990028.1	*TaWRKY118*	IIc									
Traes_3AL_4769A72F1.1	*TaWRKY125*	IIc									
Traes_3AL_AB2BAE660.1	*TaWRKY129*	IIc				*					*
Traes_3AL_140B829CB.2	*TaWRKY130*	IIc		*			*				*
Traes_4AL_234E1CDF6.1	*TaWRKY140*	IIc									
Traes_4DS_CFC487CE5.2	*TaWRKY143*	IIc		*		*	*	*			*
Traes_6DS_BF71C1557.2	*TaWRKY166*	IIc									
Traes_2AL_434E9F101.1	*TaWRKY22*	IId				*	*	*		*	*
Traes_2AL_15A7BB684.1	*TaWRKY27*	IId		*		*		*			*
Traes_2BL_6B75B32E3.1	*TaWRKY35*	IId		*		*		*			
Traes_2DL_F600B5FDF.1	*TaWRKY37*	IId		*			*				*
Traes_4AL_98B1C762B.1	*TaWRKY67*	IId					*				*
Traes_4BS_CE839571B.2	*TaWRKY71*	IId						*			*
Traes_4DS_3BE557D5C.2	*TaWRKY73*	IId									*
Traes_5AL_6F7D1D441.1	*TaWRKY80*	IId				*	*	*			*
Traes_5BS_E0345D5DF.2	*TaWRKY81*	IId		*			*	*			*
Traes_5BL_8688F70C9.1	*TaWRKY89*	IId					*				*
Traes_5BL_E294922A9.2	*TaWRKY91*	IId					*				*
Traes_5DS_D83DEA9B0.1	*TaWRKY93*	IId		*		*	*	*			*
Traes_5BL_AEF9FE805.2	*TaWRKY154*	IId					*				
Traes_1AL_4E924201A.1	*TaWRKY3*	IIe	*	*		*	*				*
TRAES3BF180700010CFD_t1	*TaWRKY51*	IIe								*	*
TRAES3BF073300120CFD_t1	*TaWRKY54*	IIe				*	*			*	*
TRAES3BF029000080CFD_t1	*TaWRKY55*	IIe		*			*				*
Traes_5DL_E4A6D1889.2	*TaWRKY100*	IIe				*	*			*	*
Traes_6AL_A5FB7CFA5.1	*TaWRKY103*	IIe								*	*
Traes_6DL_D4F2CDDDC.1	*TaWRKY107*	IIe		*				*			*
Traes_1BL_794E99FF5.1	*TaWRKY119*	IIe		*		*	*				*
Traes_5BL_B9DD3E76F.1	*TaWRKY155*	IIe				*	*	*			*
Traes_1AL_309623B48.1	*TaWRKY4*	III					*				*
Traes_1AL_B24F28600.1	*TaWRKY5*	III									*
Traes_1BL_46340D685.1	*TaWRKY9*	III		*			*				*
Traes_1BL_B4AFDB663.1	*TaWRKY10*	III				*	*	*		*	*
Traes_1BL_73811B853.1	*TaWRKY13*	III		*							*
Traes_1DL_DFE1721E0.1	*TaWRKY14*	III		*			*				*
Traes_2AS_6269D889E.1	*TaWRKY24*	III		*		*		*		*	*
Traes_2BS_B65714572.1	*TaWRKY29*	III				*	*				*
Traes_2DS_AD8820C42.1	*TaWRKY40*	III				*				*	*
Traes_3AL_2297D6E18.1	*TaWRKY44*	III		*			*	*			*
TRAES3BF051200110CFD_t1	*TaWRKY46*	III					*			*	*
TRAES3BF081400030CFD_t1	*TaWRKY56*	III					*	*			*
TRAES3BF001300030CFD_t1	*TaWRKY57*	III		*			*	*		*	*
TRAES3BF005100010CFD_t1	*TaWRKY58*	III		*	*			*			*
TRAES3BF005100020CFD_t1	*TaWRKY59*	III				*	*				*
TRAES3BF005100030CFD_t1	*TaWRKY60*	III				*	*				*
Traes_3DL_48F7A19D2.1	*TaWRKY64*	III		*			*	*			*
Traes_4AS_70DF607CC.1	*TaWRKY65*	III					*	*			*
Traes_4AL_9E0D1CFA6.1	*TaWRKY66*	III		*			*				*
Traes_4BS_A6D9EB0E5.1	*TaWRKY70*	III		*			*				*
Traes_4BL_A8C6FBEB6.1	*TaWRKY72*	III					*				*
Traes_4DS_FE38A59D0.1	*TaWRKY74*	III		*			*				*
Traes_4DL_3140A8240.1	*TaWRKY76*	III		*			*				*
Traes_5AL_B4E8A3115.2	*TaWRKY78*	III					*	*			*
Traes_5BL_0A3D332A8.1	*TaWRKY84*	III		*			*				*
Traes_5BL_175E7FC38.1	*TaWRKY85*	III		*			*				*
Traes_5DL_C93641E43.1	*TaWRKY92*	III					*	*			*
Traes_5DL_5C93510D5.1	*TaWRKY95*	III		*			*	*			*
Traes_6AS_68775100B.1	*TaWRKY101*	III				*	*	*			*
Traes_7DS_01F74C6F3.1	*TaWRKY110*	III					*	*			*
Traes_7DL_A9EF00572.1	*TaWRKY112*	III					*				*
Traes_2AS_1AFFE8DA6.1	*TaWRKY121*	III					*				*
Traes_2DL_362A1F535.1	*TaWRKY122*	III									
Traes_3B_8B0D448D8.1	*TaWRKY132*	III					*				*
Traes_3DL_2551BF2C1.1	*TaWRKY136*	III									
Traes_4AS_0DA136E0E.1	*TaWRKY142*	III					*		*		*
Traes_5DL_09F1F8F79.1	*TaWRKY160*	III					*				*
Traes_7BL_53AA25AA1.1	*TaWRKY169*	III		*				*			*

Transcript ID	Proposed name	Group	Sp1	G-Box	GAG-motif	I-box	GT1-motif	MNF1	ACE	Box I	Box 4	GATA-motif	GA-motif
			light responsiveness	light responsiveness	light responsiveness	light responsiveness	light responsiveness	light responsiveness	light responsiveness	light responsiveness	light responsiveness	light responsiveness	light responsiveness
Traes_1AS_F3EAEC435.1	*TaWRKY1*	I	*	*	*		*				*		*
Traes_1BS_EF67E5A24.1	*TaWRKY8*	I	*	*	*								
Traes_2AS_C407071E4.2	*TaWRKY23*	I	*	*		*	*		*		*		*
Traes_2AS_0186B9E4F.2	*TaWRKY25*	I	*	*		*					*		*
Traes_2AL_409AB7647.1	*TaWRKY26*	I	*	*	*			*	*	*		*	*
Traes_2BL_2BA3A755A.1	*TaWRKY30*	I	*	*	*		*	*		*			*
Traes_2BS_380EC4D1E.1	*TaWRKY32*	I	*	*			*	*	*			*	
Traes_2BS_D435A8999.1	*TaWRKY33*	I	*	*			*	*					*
Traes_2DS_97E3E7CFC.1	*TaWRKY39*	I	*	*	*		*	*	*	*			
Traes_2DS_F6FBC974C.2	*TaWRKY41*	I	*	*		*		*	*	*	*		
Traes_4BL_EFEC50B26.2	*TaWRKY69*	I		*	*	*				*			*
Traes_4DS_DC3C9DC42.2	*TaWRKY75*	I	*		*	*		*			*	*	*
Traes_5AL_ED3ADED51.3	*TaWRKY77*	I	*	*	*								
Traes_5BS_C46781248.1	*TaWRKY82*	I		*		*	*					*	*
Traes_5BL_A522C62D1.1	*TaWRKY83*	I	*			*			*			*	
Traes_5BL_17A712C94.1	*TaWRKY88*	I	*			*		*		*	*	*	*
Traes_5DS_5DEA5C9E3.1	*TaWRKY94*	I		*	*	*	*			*			*
Traes_5DL_21F7C6BF7.2	*TaWRKY96*	I	*			*							
Traes_5DL_46E3AC8D6.1	*TaWRKY98*	I		*		*			*	*	*		
Traes_5DL_4BA2CC560.2	*TaWRKY99*	I	*		*		*	*					*
Traes_7BL_A46F1A830.2	*TaWRKY109*	I		*	*	*	*		*			*	
Traes_7DS_24C563960.1	*TaWRKY111*	I	*		*	*		*	*	*			*
Traes_1DS_A6733B734.1	*TaWRKY120*	I	*	*	*	*		*					
Traes_3B_990298FF5.1	*TaWRKY133*	I	*	*	*		*		*	*			
Traes_4AL_2EEECCC4B.1	*TaWRKY141*	I		*	*			*					
Traes_5AL_E644A6A0B.1	*TaWRKY145*	I	*	*	*		*	*					*
Traes_5BL_D3C383CF5.1	*TaWRKY152*	I		*			*	*	*		*		
Traes_7AL_48C81DE03.1	*TaWRKY168*	I	*										
Traes_7AL_48C81DE031.1	*TaWRKY171*	I	*										
Traes_5BL_8BEF7F9CD.1	*TaWRKY86*	IIa	*	*				*					
Traes_5BL_90757F0CC.1	*TaWRKY87*	IIa	*		*						*		
Traes_7DL_5968FA56C.1	*TaWRKY113*	IIa	*	*									
Traes_7DL_B09854286.1	*TaWRKY114*	IIa	*			*		*					*
Traes_7DL_F849918EA.2	*TaWRKY115*	IIa	*	*		*						*	*
Traes_6AS_DA75BB1FD.1	*TaWRKY161*	IIa		*		*	*	*	*		*		*
TRAES3BF003800010CFD_t1	*TaWRKY47*	IIb	*	*		*	*		*				
Traes_4AL_3E11167D9.1	*TaWRKY68*	IIb	*	*			*				*		
Traes_6AL_0C0899C15.1	*TaWRKY104*	IIb		*		*	*		*	*	*	*	
Traes_7AS_C9DF68E53.2	*TaWRKY108*	IIb	*	*			*				*		
Traes_6DL_AB95B0CE0.1	*TaWRKY164*	IIb		*			*		*	*	*	*	
Traes_1AS_1432A2F79.1	*TaWRKY2*	IIc	*		*	*	*	*			*		
Traes_1AL_0404BC790.1	*TaWRKY6*	IIc	*	*			*	*	*				
Traes_1AL_9ADA7A031.1	*TaWRKY7*	IIc	*	*	*						*		*
Traes_1BL_1D865A8CC.1	*TaWRKY11*	IIc	*	*		*						*	*
Traes_1BL_9AFA4B870.1	*TaWRKY12*	IIc	*	*		*	*		*				
Traes_1DL_D550418641.2	*TaWRKY15*	IIc	*	*	*	*				*		*	*
Traes_1DL_5BAB0B6BC.1	*TaWRKY16*	IIc	*			*	*					*	
Traes_1DL_D1EC7DEA6.1	*TaWRKY17*	IIc		*						*			
Traes_1DL_46428511F.1	*TaWRKY18*	IIc	*	*			*		*			*	
Traes_2AS_D0C21ADB5.1	*TaWRKY19*	IIc	*	*	*			*		*		*	
Traes_2AL_1B43EA59E.1	*TaWRKY20*	IIc	*	*		*	*				*		
Traes_2AL_B1270662B.1	*TaWRKY21*	IIc	*	*		*		*					
Traes_2BS_F3097F116.1	*TaWRKY28*	IIc	*	*						*			
Traes_2BL_A69F6C5DF.1	*TaWRKY31*	IIc	*	*	*			*			*		
Traes_2BL_A5BFA97B9.1	*TaWRKY34*	IIc	*	*	*	*	*				*		*
Traes_2DS_0F2500A60.1	*TaWRKY36*	IIc	*						*	*			
Traes_2DL_4F9F8F1F0.1	*TaWRKY38*	IIc	*	*			*				*	*	
Traes_3AL_67ECA2932.1	*TaWRKY42*	IIc	*	*	*	*				*			
Traes_3AL_1B73D2C12.1	*TaWRKY43*	IIc	*		*	*				*		*	*
TRAES3BF066700160CFD_t1	*TaWRKY45*	IIc	*	*	*				*				
TRAES3BF021300010CFD_t1	*TaWRKY48*	IIc	*	*	*				*	*	*		
TRAES3BF267200010CFD_t1	*TaWRKY49*	IIc	*	*				*					
TRAES3BF021100090CFD_t1	*TaWRKY50*	IIc	*	*	*				*	*	*		
TRAES3BF058500060CFD_t1	*TaWRKY52*	IIc	*	*			*		*			*	
TRAES3BF111700140CFD_t1	*TaWRKY53*	IIc	*	*	*	*		*		*		*	
TRAES3BF090100100CFD_t1	*TaWRKY61*	IIc	*		*			*					
TRAES3BF045500040CFD_t1	*TaWRKY62*	IIc	*	*		*	*	*	*		*		
Traes_3DL_DF0D3F3FE.1	*TaWRKY63*	IIc	*		*	*	*						*
Traes_5AL_E566BD64E.1	*TaWRKY79*	IIc	*	*		*				*			
Traes_5BL_C1D6B6B74.2	*TaWRKY90*	IIc	*	*	*				*	*		*	
Traes_5DL_7E2053226.2	*TaWRKY97*	IIc	*	*			*	*	*				

Traes_6AL_BA4636569.1	*TaWRKY102*	IIc	*	*	*	*	*						*
Traes_6BL_DD840863A.1	*TaWRKY105*	IIc	*	*					*		*		
Traes_6DL_D29E210A1.1	*TaWRKY106*	IIc	*			*		*		*			
Traes_1AL_F64E07A92.1	*TaWRKY116*	IIc	*	*	*		*		*		*	*	*
Traes_1BL_B15990028.1	*TaWRKY118*	IIc	*						*				
Traes_3AL_4769A72F1.1	*TaWRKY125*	IIc								*			*
Traes_3AL_AB2BAE660.1	*TaWRKY129*	IIc	*	*			*		*			*	
Traes_3AL_140B829CB.2	*TaWRKY130*	IIc	*	*	*	*		*	*		*		
Traes_4AL_234E1CDF6.1	*TaWRKY140*	IIc											
Traes_4DS_CFC487CE5.2	*TaWRKY143*	IIc	*	*	*		*		*		*		*
Traes_6DS_BF71C1557.2	*TaWRKY166*	IIc											
Traes_2AL_434E9F101.1	*TaWRKY22*	IId	*	*		*		*					
Traes_2AL_15A7BB684.1	*TaWRKY27*	IId		*		*					*		
Traes_2BL_6B75B32E3.1	*TaWRKY35*	IId	*			*	*			*	*		
Traes_2DL_F600B5FDF.1	*TaWRKY37*	IId		*	*								
Traes_4AL_98B1C762B.1	*TaWRKY67*	IId	*	*	*	*				*		*	*
Traes_4BS_CE839571B.2	*TaWRKY71*	IId	*	*	*				*				
Traes_4DS_3BE557D5C.2	*TaWRKY73*	IId	*	*	*			*		*			
Traes_5AL_6F7D1D441.1	*TaWRKY80*	IId	*	*		*		*	*				
Traes_5BS_E0345D5DF.2	*TaWRKY81*	IId	*	*				*				*	
Traes_5BL_8688F70C9.1	*TaWRKY89*	IId	*	*	*			*	*				*
Traes_5BL_E294922A9.2	*TaWRKY91*	IId	*	*	*	*	*		*	*			*
Traes_5DS_D83DEA9B0.1	*TaWRKY93*	IId	*	*	*			*	*	*		*	
Traes_5BL_AEF9FE805.2	*TaWRKY154*	IId	*										
Traes_1AL_4E924201A.1	*TaWRKY3*	IIe	*	*	*	*	*	*			*	*	*
TRAES3BF180700010CFD_t1	*TaWRKY51*	IIe	*	*	*	*		*	*	*	*		
TRAES3BF073300120CFD_t1	*TaWRKY54*	IIe	*	*		*						*	
TRAES3BF029000080CFD_t1	*TaWRKY55*	IIe	*	*	*						*		*
Traes_5DL_E4A6D1889.2	*TaWRKY100*	IIe	*	*	*								
Traes_6AL_A5FB7CFA5.1	*TaWRKY103*	IIe	*					*	*				
Traes_6DL_D4F2CDDDC.1	*TaWRKY107*	IIe	*	*			*				*		
Traes_1BL_794E99FF5.1	*TaWRKY119*	IIe	*		*	*	*			*	*	*	
Traes_5BL_B9DD3E76F.1	*TaWRKY155*	IIe	*	*			*						
Traes_1AL_309623B48.1	*TaWRKY4*	III	*	*	*		*		*	*			*
Traes_1AL_B24F28600.1	*TaWRKY5*	III	*	*		*	*	*		*	*	*	
Traes_1BL_46340D685.1	*TaWRKY9*	III	*	*	*	*			*	*			
Traes_1BL_B4AFDB663.1	*TaWRKY10*	III	*	*		*	*	*			*	*	
Traes_1BL_73811B853.1	*TaWRKY13*	III	*	*				*	*		*		
Traes_1DL_DFE1721E0.1	*TaWRKY14*	III	*		*	*				*			*
Traes_2AS_6269D889E.1	*TaWRKY24*	III			*		*	*				*	*
Traes_2BS_B65714572.1	*TaWRKY29*	III	*	*		*	*				*		
Traes_2DS_AD8820C42.1	*TaWRKY40*	III	*				*		*			*	*
Traes_3AL_2297D6E18.1	*TaWRKY44*	III	*	*		*		*			*		
TRAES3BF051200110CFD_t1	*TaWRKY46*	III	*				*	*		*			
TRAES3BF081400030CFD_t1	*TaWRKY56*	III		*			*	*		*			
TRAES3BF001300030CFD_t1	*TaWRKY57*	III	*	*		*	*		*				
TRAES3BF005100010CFD_t1	*TaWRKY58*	III	*	*	*	*			*				
TRAES3BF005100020CFD_t1	*TaWRKY59*	III	*		*			*		*		*	*
TRAES3BF005100030CFD_t1	*TaWRKY60*	III	*		*			*		*		*	*
Traes_3DL_48F7A19D2.1	*TaWRKY64*	III	*	*		*		*			*	*	
Traes_4AS_70DF607CC.1	*TaWRKY65*	III	*	*	*		*						
Traes_4AL_9E0D1CFA6.1	*TaWRKY66*	III	*	*	*		*	*				*	
Traes_4BS_A6D9EB0E5.1	*TaWRKY70*	III	*				*		*	*			
Traes_4BL_A8C6FBEB6.1	*TaWRKY72*	III	*	*	*		*			*			
Traes_4DS_FE38A59D0.1	*TaWRKY74*	III	*				*						
Traes_4DL_3140A8240.1	*TaWRKY76*	III	*	*	*								
Traes_5AL_B4E8A3115.2	*TaWRKY78*	III	*	*			*				*		
Traes_5BL_0A3D332A8.1	*TaWRKY84*	III	*		*	*		*		*	*	*	
Traes_5BL_175E7FC38.1	*TaWRKY85*	III	*	*		*				*		*	*
Traes_5DL_C93641E43.1	*TaWRKY92*	III	*	*	*	*	*	*					
Traes_5DL_5C93510D5.1	*TaWRKY95*	III	*			*			*			*	
Traes_6AS_68775100B.1	*TaWRKY101*	III	*	*							*		
Traes_7DS_01F74C6F3.1	*TaWRKY110*	III	*	*	*	*			*	*		*	
Traes_7DL_A9EF00572.1	*TaWRKY112*	III	*	*			*	*	*	*		*	
Traes_2AS_1AFFE8DA6.1	*TaWRKY121*	III	*	*			*			*	*		
Traes_2DL_362A1F535.1	*TaWRKY122*	III											
Traes_3B_8B0D448D8.1	*TaWRKY132*	III	*	*	*	*	*		*	*			
Traes_3DL_2551BF2C1.1	*TaWRKY136*	III											
Traes_4AS_0DA136E0E.1	*TaWRKY142*	III	*	*					*	*	*		*
Traes_5DL_09F1F8F79.1	*TaWRKY160*	III	*	*	*	*		*		*		*	*
Traes_7BL_53AA25AA1.1	*TaWRKY169*	III	*	*	*	*			*				

Transcript ID	Proposed name	Group	CATT-motif	AE-box	TCT-motif	TCCC-motif	ATCT-motif	3-AF1 binding site	rbcS-CMA7a	LAMP-element	ATC-motif	Box III	box II
			light responsiveness	light responsiveness	light responsiveness	light responsiveness	light responsiveness	light responsiveness	light responsiveness	light responsiveness	light responsiveness	light responsiveness	light responsiveness
Traes_1AS_F3EAEC435.1	*TaWRKY1*	I		*									
Traes_1BS_EF67E5A24.1	*TaWRKY8*	I			*	*			*				
Traes_2AS_C407071E4.2	*TaWRKY23*	I	*						*	*			
Traes_2AS_0186B9E4F.2	*TaWRKY25*	I	*		*			*					
Traes_2AL_409AB7647.1	*TaWRKY26*	I		*	*			*		*			
Traes_2BL_2BA3A755A.1	*TaWRKY30*	I		*	*					*			
Traes_2BS_380EC4D1E.1	*TaWRKY32*	I										*	
Traes_2BS_D435A8999.1	*TaWRKY33*	I											
Traes_2DS_97E3E7CFC.1	*TaWRKY39*	I		*						*			
Traes_2DS_F6FBC974C.2	*TaWRKY41*	I					*		*				
Traes_4BL_EFEC50B26.2	*TaWRKY69*	I				*							
Traes_4DS_DC3C9DC42.2	*TaWRKY75*	I							*				
Traes_5AL_ED3ADED51.3	*TaWRKY77*	I											
Traes_5BS_C46781248.1	*TaWRKY82*	I					*						
Traes_5BL_A522C62D1.1	*TaWRKY83*	I	*	*					*				
Traes_5BL_17A712C94.1	*TaWRKY88*	I					*						
Traes_5DS_5DEA5C9E3.1	*TaWRKY94*	I		*	*								
Traes_5DL_21F7C6BF7.2	*TaWRKY96*	I											
Traes_5DL_46E3AC8D6.1	*TaWRKY98*	I				*		*					
Traes_5DL_4BA2CC560.2	*TaWRKY99*	I		*	*								
Traes_7BL_A46F1A830.2	*TaWRKY109*	I				*							
Traes_7DS_24C563960.1	*TaWRKY111*	I	*		*								
Traes_1DS_A6733B734.1	*TaWRKY120*	I	*		*			*			*	*	
Traes_3B_990298FF5.1	*TaWRKY133*	I					*						
Traes_4AL_2EEECCC4B.1	*TaWRKY141*	I				*							
Traes_5AL_E644A6A0B.1	*TaWRKY145*	I	*	*	*								
Traes_5BL_D3C383CF5.1	*TaWRKY152*	I				*			*				
Traes_7AL_48C81DE03.1	*TaWRKY168*	I				*							
Traes_7AL_48C81DE031.1	*TaWRKY171*	I				*							
Traes_5BL_8BEF7F9CD.1	*TaWRKY86*	IIa		*							*		
Traes_5BL_90757F0CC.1	*TaWRKY87*	IIa	*										
Traes_7DL_5968FA56C.1	*TaWRKY113*	IIa										*	
Traes_7DL_B09854286.1	*TaWRKY114*	IIa		*			*						
Traes_7DL_F849918EA.2	*TaWRKY115*	IIa				*					*		
Traes_6AS_DA75BB1FD.1	*TaWRKY161*	IIa		*			*			*	*		
TRAES3BF003800010CFD_t1	*TaWRKY47*	IIb				*		*			*		*
Traes_4AL_3E11167D9.1	*TaWRKY68*	IIb			*				*				
Traes_6AL_0C0899C15.1	*TaWRKY104*	IIb					*	*				*	
Traes_7AS_C9DF68E53.2	*TaWRKY108*	IIb			*	*	*						
Traes_6DL_AB95B0CE0.1	*TaWRKY164*	IIb						*					
Traes_1AS_1432A2F79.1	*TaWRKY2*	IIc								*			
Traes_1AL_0404BC790.1	*TaWRKY6*	IIc			*		*						
Traes_1AL_9ADA7A031.1	*TaWRKY7*	IIc	*										
Traes_1BL_1D865A8CC.1	*TaWRKY11*	IIc	*	*	*								
Traes_1BL_9AFA4B870.1	*TaWRKY12*	IIc			*							*	*
Traes_1DL_D550418641.2	*TaWRKY15*	IIc	*		*		*						
Traes_1DL_5BAB0B6BC.1	*TaWRKY16*	IIc	*	*	*		*					*	
Traes_1DL_D1EC7DEA6.1	*TaWRKY17*	IIc											
Traes_1DL_46428511F.1	*TaWRKY18*	IIc	*										
Traes_2AS_D0C21ADB5.1	*TaWRKY19*	IIc				*						*	*
Traes_2AL_1B43EA59E.1	*TaWRKY20*	IIc	*				*	*					
Traes_2AL_B1270662B.1	*TaWRKY21*	IIc											
Traes_2BS_F3097F116.1	*TaWRKY28*	IIc	*			*							
Traes_2BL_A69F6C5DF.1	*TaWRKY31*	IIc	*			*							
Traes_2BL_A5BFA97B9.1	*TaWRKY34*	IIc	*	*									
Traes_2DS_0F2500A60.1	*TaWRKY36*	IIc					*						
Traes_2DL_4F9F8F1F0.1	*TaWRKY38*	IIc			*	*					*		
Traes_3AL_67ECA2932.1	*TaWRKY42*	IIc											*
Traes_3AL_1B73D2C12.1	*TaWRKY43*	IIc											
TRAES3BF066700160CFD_t1	*TaWRKY45*	IIc		*									*
TRAES3BF021300010CFD_t1	*TaWRKY48*	IIc		*									
TRAES3BF267200010CFD_t1	*TaWRKY49*	IIc										*	
TRAES3BF021100090CFD_t1	*TaWRKY50*	IIc		*									
TRAES3BF058500060CFD_t1	*TaWRKY52*	IIc		*									
TRAES3BF111700140CFD_t1	*TaWRKY53*	IIc			*								
TRAES3BF090100100CFD_t1	*TaWRKY61*	IIc			*								
TRAES3BF045500040CFD_t1	*TaWRKY62*	IIc	*					*		*			
Traes_3DL_DF0D3F3FE.1	*TaWRKY63*	IIc	*	*								*	
Traes_5AL_E566BD64E.1	*TaWRKY79*	IIc								*	*		
Traes_5BL_C1D6B6B74.2	*TaWRKY90*	IIc	*				*		*				
Traes_5DL_7E2053226.2	*TaWRKY97*	IIc	*			*							

Traes_6AL_BA4636569.1	*TaWRKY102*	IIc	*		*								
Traes_6BL_DD840863A.1	*TaWRKY105*	IIc						*	*	*			
Traes_6DL_D29E210A1.1	*TaWRKY106*	IIc	*	*	*		*	*	*				
Traes_1AL_F64E07A92.1	*TaWRKY116*	IIc											
Traes_1BL_B15990028.1	*TaWRKY118*	IIc											
Traes_3AL_4769A72F1.1	*TaWRKY125*	IIc										*	
Traes_3AL_AB2BAE660.1	*TaWRKY129*	IIc	*			*							
Traes_3AL_140B829CB.2	*TaWRKY130*	IIc	*			*							
Traes_4AL_234E1CDF6.1	*TaWRKY140*	IIc											
Traes_4DS_CFC487CE5.2	*TaWRKY143*	IIc											*
Traes_6DS_BF71C1557.2	*TaWRKY166*	IIc											
Traes_2AL_434E9F101.1	*TaWRKY22*	IId		*									
Traes_2AL_15A7BB684.1	*TaWRKY27*	IId		*		*							
Traes_2BL_6B75B32E3.1	*TaWRKY35*	IId											
Traes_2DL_F600B5FDF.1	*TaWRKY37*	IId			*	*	*						
Traes_4AL_98B1C762B.1	*TaWRKY67*	IId	*	*			*						
Traes_4BS_CE839571B.2	*TaWRKY71*	IId		*									
Traes_4DS_3BE557D5C.2	*TaWRKY73*	IId		*									*
Traes_5AL_6F7D1D441.1	*TaWRKY80*	IId	*							*	*	*	*
Traes_5BS_E0345D5DF.2	*TaWRKY81*	IId	*			*							
Traes_5BL_8688F70C9.1	*TaWRKY89*	IId											*
Traes_5BL_E294922A9.2	*TaWRKY91*	IId			*								
Traes_5DS_D83DEA9B0.1	*TaWRKY93*	IId					*						
Traes_5BL_AEF9FE805.2	*TaWRKY154*	IId											
Traes_1AL_4E924201A.1	*TaWRKY3*	IIe							*		*		
TRAES3BF180700010CFD_t1	*TaWRKY51*	IIe				*						*	
TRAES3BF073300120CFD_t1	*TaWRKY54*	IIe		*									*
TRAES3BF029000080CFD_t1	*TaWRKY55*	IIe			*	*	*						
Traes_5DL_E4A6D1889.2	*TaWRKY100*	IIe		*		*	*	*					
Traes_6AL_A5FB7CFA5.1	*TaWRKY103*	IIe							*				
Traes_6DL_D4F2CDDDC.1	*TaWRKY107*	IIe	*				*						
Traes_1BL_794E99FF5.1	*TaWRKY119*	IIe			*	*			*		*		
Traes_5BL_B9DD3E76F.1	*TaWRKY155*	IIe				*	*	*					
Traes_1AL_309623B48.1	*TaWRKY4*	III	*	*			*						
Traes_1AL_B24F28600.1	*TaWRKY5*	III			*	*		*		*			
Traes_1BL_46340D685.1	*TaWRKY9*	III		*						*	*		
Traes_1BL_B4AFDB663.1	*TaWRKY10*	III	*	*									
Traes_1BL_73811B853.1	*TaWRKY13*	III	*										
Traes_1DL_DFE1721E0.1	*TaWRKY14*	III		*									
Traes_2AS_6269D889E.1	*TaWRKY24*	III											
Traes_2BS_B65714572.1	*TaWRKY29*	III							*				
Traes_2DS_AD8820C42.1	*TaWRKY40*	III								*			
Traes_3AL_2297D6E18.1	*TaWRKY44*	III	*		*		*						
TRAES3BF051200110CFD_t1	*TaWRKY46*	III		*	*	*							
TRAES3BF081400030CFD_t1	*TaWRKY56*	III											
TRAES3BF001300030CFD_t1	*TaWRKY57*	III					*	*	*				
TRAES3BF005100010CFD_t1	*TaWRKY58*	III	*										
TRAES3BF005100020CFD_t1	*TaWRKY59*	III			*	*							
TRAES3BF005100030CFD_t1	*TaWRKY60*	III			*	*							
Traes_3DL_48F7A19D2.1	*TaWRKY64*	III	*		*		*						
Traes_4AS_70DF607CC.1	*TaWRKY65*	III		*									
Traes_4AL_9E0D1CFA6.1	*TaWRKY66*	III	*	*	*					*			
Traes_4BS_A6D9EB0E5.1	*TaWRKY70*	III			*		*	*					
Traes_4BL_A8C6FBEB6.1	*TaWRKY72*	III		*		*							
Traes_4DS_FE38A59D0.1	*TaWRKY74*	III			*		*	*					
Traes_4DL_3140A8240.1	*TaWRKY76*	III	*	*	*			*				*	
Traes_5AL_B4E8A3115.2	*TaWRKY78*	III		*						*			
Traes_5BL_0A3D332A8.1	*TaWRKY84*	III	*	*	*						*		
Traes_5BL_175E7FC38.1	*TaWRKY85*	III				*							*
Traes_5DL_C93641E43.1	*TaWRKY92*	III			*				*		*		
Traes_5DL_5C93510D5.1	*TaWRKY95*	III		*	*						*		
Traes_6AS_68775100B.1	*TaWRKY101*	III									*		
Traes_7DS_01F74C6F3.1	*TaWRKY110*	III					*						
Traes_7DL_A9EF00572.1	*TaWRKY112*	III	*				*	*				*	
Traes_2AS_1AFFE8DA6.1	*TaWRKY121*	III	*										
Traes_2DL_362A1F535.1	*TaWRKY122*	III											
Traes_3B_8B0D448D8.1	*TaWRKY132*	III	*			*	*						
Traes_3DL_2551BF2C1.1	*TaWRKY136*	III											
Traes_4AS_0DA136E0E.1	*TaWRKY142*	III											
Traes_5DL_09F1F8F79.1	*TaWRKY160*	III											*
Traes_7BL_53AA25AA1.1	*TaWRKY169*	III											

Transcript ID	Proposed name	Group	TGG-motif	chs-Unit 1 m1	chs-CMA2a	Box II	L-box	GTGGC-motif	CG-motif	C-box	Gap-box	CAG-motif	AAAC-motif
			light responsiveness	light responsiveness	light responsiveness	light responsiveness	light responsiveness	light responsiveness	light responsiveness	light responsiveness	part of a light responsive element	part of a light response element	light responsiveness
Traes_1AS_F3EAEC435.1	*TaWRKY1*	I								*			
Traes_1BS_EF67E5A24.1	*TaWRKY8*	I											
Traes_2AS_C407071E4.2	*TaWRKY23*	I	*										
Traes_2AS_0186B9E4F.2	*TaWRKY25*	I											
Traes_2AL_409AB7647.1	*TaWRKY26*	I			*								
Traes_2BL_2BA3A755A.1	*TaWRKY30*	I			*								
Traes_2BS_380EC4D1E.1	*TaWRKY32*	I											
Traes_2BS_D435A8999.1	*TaWRKY33*	I										*	
Traes_2DS_97E3E7CFC.1	*TaWRKY39*	I	*										
Traes_2DS_F6FBC974C.2	*TaWRKY41*	I											
Traes_4BL_EFEC50B26.2	*TaWRKY69*	I											
Traes_4DS_DC3C9DC42.2	*TaWRKY75*	I											
Traes_5AL_ED3ADED51.3	*TaWRKY77*	I											
Traes_5BS_C46781248.1	*TaWRKY82*	I											
Traes_5BL_A522C62D1.1	*TaWRKY83*	I											
Traes_5BL_17A712C94.1	*TaWRKY88*	I											
Traes_5DS_5DEA5C9E3.1	*TaWRKY94*	I											
Traes_5DL_21F7C6BF7.2	*TaWRKY96*	I	*										
Traes_5DL_46E3AC8D6.1	*TaWRKY98*	I	*										
Traes_5DL_4BA2CC560.2	*TaWRKY99*	I											
Traes_7BL_A46F1A830.2	*TaWRKY109*	I									*		
Traes_7DS_24C563960.1	*TaWRKY111*	I											
Traes_1DS_A6733B734.1	*TaWRKY120*	I						*					
Traes_3B_990298FF5.1	*TaWRKY133*	I											
Traes_4AL_2EEECCC4B.1	*TaWRKY141*	I						*				*	
Traes_5AL_E644A6A0B.1	*TaWRKY145*	I											
Traes_5BL_D3C383CF5.1	*TaWRKY152*	I											
Traes_7AL_48C81DE03.1	*TaWRKY168*	I											
Traes_7AL_48C81DE031.1	*TaWRKY171*	I											
Traes_5BL_8BEF7F9CD.1	*TaWRKY86*	IIa											*
Traes_5BL_90757F0CC.1	*TaWRKY87*	IIa											
Traes_7DL_5968FA56C.1	*TaWRKY113*	IIa		*									
Traes_7DL_B09854286.1	*TaWRKY114*	IIa		*									
Traes_7DL_F849918EA.2	*TaWRKY115*	IIa											
Traes_6AS_DA75BB1FD.1	*TaWRKY161*	IIa				*	*					*	
TRAES3BF003800010CFD_t1	*TaWRKY47*	IIb				*				*			
Traes_4AL_3E11167D9.1	*TaWRKY68*	IIb											
Traes_6AL_0C0899C15.1	*TaWRKY104*	IIb											
Traes_7AS_C9DF68E53.2	*TaWRKY108*	IIb									*		
Traes_6DL_AB95B0CE0.1	*TaWRKY164*	IIb											
Traes_1AS_1432A2F79.1	*TaWRKY2*	IIc											
Traes_1AL_0404BC790.1	*TaWRKY6*	IIc										*	
Traes_1AL_9ADA7A031.1	*TaWRKY7*	IIc											
Traes_1BL_1D865A8CC.1	*TaWRKY11*	IIc											
Traes_1BL_9AFA4B870.1	*TaWRKY12*	IIc				*							
Traes_1DL_D550418641.2	*TaWRKY15*	IIc											
Traes_1DL_5BAB0B6BC.1	*TaWRKY16*	IIc											
Traes_1DL_D1EC7DEA6.1	*TaWRKY17*	IIc											
Traes_1DL_46428511F.1	*TaWRKY18*	IIc											
Traes_2AS_D0C21ADB5.1	*TaWRKY19*	IIc				*			*				
Traes_2AL_1B43EA59E.1	*TaWRKY20*	IIc											
Traes_2AL_B1270662B.1	*TaWRKY21*	IIc		*									
Traes_2BS_F3097F116.1	*TaWRKY28*	IIc											
Traes_2BL_A69F6C5DF.1	*TaWRKY31*	IIc		*									
Traes_2BL_A5BFA97B9.1	*TaWRKY34*	IIc							*				
Traes_2DS_0F2500A60.1	*TaWRKY36*	IIc											
Traes_2DL_4F9F8F1F0.1	*TaWRKY38*	IIc											
Traes_3AL_67ECA2932.1	*TaWRKY42*	IIc							*				
Traes_3AL_1B73D2C12.1	*TaWRKY43*	IIc			*								
TRAES3BF066700160CFD_t1	*TaWRKY45*	IIc	*										
TRAES3BF021300010CFD_t1	*TaWRKY48*	IIc		*		*		*					
TRAES3BF267200010CFD_t1	*TaWRKY49*	IIc						*					
TRAES3BF021100090CFD_t1	*TaWRKY50*	IIc		*		*		*					
TRAES3BF058500060CFD_t1	*TaWRKY52*	IIc											
TRAES3BF111700140CFD_t1	*TaWRKY53*	IIc											
TRAES3BF090100100CFD_t1	*TaWRKY61*	IIc	*	*									
TRAES3BF045500040CFD_t1	*TaWRKY62*	IIc			*				*				
Traes_3DL_DF0D3F3FE.1	*TaWRKY63*	IIc											
Traes_5AL_E566BD64E.1	*TaWRKY79*	IIc											
Traes_5BL_C1D6B6B74.2	*TaWRKY90*	IIc											
Traes_5DL_7E2053226.2	*TaWRKY97*	IIc											

Traes_6AL_BA4636569.1	*TaWRKY102*	IIc										
Traes_6BL_DD840863A.1	*TaWRKY105*	IIc										
Traes_6DL_D29E210A1.1	*TaWRKY106*	IIc	*									
Traes_1AL_F64E07A92.1	*TaWRKY116*	IIc					*					
Traes_1BL_B15990028.1	*TaWRKY118*	IIc										
Traes_3AL_4769A72F1.1	*TaWRKY125*	IIc		*								
Traes_3AL_AB2BAE660.1	*TaWRKY129*	IIc										
Traes_3AL_140B829CB.2	*TaWRKY130*	IIc				*	*	*				
Traes_4AL_234E1CDF6.1	*TaWRKY140*	IIc										
Traes_4DS_CFC487CE5.2	*TaWRKY143*	IIc								*		
Traes_6DS_BF71C1557.2	*TaWRKY166*	IIc										
Traes_2AL_434E9F101.1	*TaWRKY22*	IId										
Traes_2AL_15A7BB684.1	*TaWRKY27*	IId										
Traes_2BL_6B75B32E3.1	*TaWRKY35*	IId										
Traes_2DL_F600B5FDF.1	*TaWRKY37*	IId										
Traes_4AL_98B1C762B.1	*TaWRKY67*	IId										
Traes_4BS_CE839571B.2	*TaWRKY71*	IId										
Traes_4DS_3BE557D5C.2	*TaWRKY73*	IId										
Traes_5AL_6F7D1D441.1	*TaWRKY80*	IId										
Traes_5BS_E0345D5DF.2	*TaWRKY81*	IId					*					*
Traes_5BL_8688F70C9.1	*TaWRKY89*	IId										
Traes_5BL_E294922A9.2	*TaWRKY91*	IId					*					
Traes_5DS_D83DEA9B0.1	*TaWRKY93*	IId					*					
Traes_5BL_AEF9FE805.2	*TaWRKY154*	IId										
Traes_1AL_4E924201A.1	*TaWRKY3*	IIe					*					
TRAES3BF180700010CFD_t1	*TaWRKY51*	IIe										
TRAES3BF073300120CFD_t1	*TaWRKY54*	IIe					*	*				
TRAES3BF029000080CFD_t1	*TaWRKY55*	IIe										
Traes_5DL_E4A6D1889.2	*TaWRKY100*	IIe										
Traes_6AL_A5FB7CFA5.1	*TaWRKY103*	IIe		*								
Traes_6DL_D4F2CDDDC.1	*TaWRKY107*	IIe										
Traes_1BL_794E99FF5.1	*TaWRKY119*	IIe									*	
Traes_5BL_B9DD3E76F.1	*TaWRKY155*	IIe										
Traes_1AL_309623B48.1	*TaWRKY4*	III										
Traes_1AL_B24F28600.1	*TaWRKY5*	III										
Traes_1BL_46340D685.1	*TaWRKY9*	III										
Traes_1BL_B4AFDB663.1	*TaWRKY10*	III										
Traes_1BL_73811B853.1	*TaWRKY13*	III										
Traes_1DL_DFE1721E0.1	*TaWRKY14*	III										
Traes_2AS_6269D889E.1	*TaWRKY24*	III			*							
Traes_2BS_B65714572.1	*TaWRKY29*	III			*							
Traes_2DS_AD8820C42.1	*TaWRKY40*	III			*							
Traes_3AL_2297D6E18.1	*TaWRKY44*	III	*	*								
TRAES3BF051200110CFD_t1	*TaWRKY46*	III		*			*					
TRAES3BF081400030CFD_t1	*TaWRKY56*	III										
TRAES3BF001300030CFD_t1	*TaWRKY57*	III										
TRAES3BF005100010CFD_t1	*TaWRKY58*	III										
TRAES3BF005100020CFD_t1	*TaWRKY59*	III			*							*
TRAES3BF005100030CFD_t1	*TaWRKY60*	III			*							*
Traes_3DL_48F7A19D2.1	*TaWRKY64*	III										
Traes_4AS_70DF607CC.1	*TaWRKY65*	III	*									
Traes_4AL_9E0D1CFA6.1	*TaWRKY66*	III			*						*	
Traes_4BS_A6D9EB0E5.1	*TaWRKY70*	III										
Traes_4BL_A8C6FBEB6.1	*TaWRKY72*	III	*									
Traes_4DS_FE38A59D0.1	*TaWRKY74*	III										
Traes_4DL_3140A8240.1	*TaWRKY76*	III	*									
Traes_5AL_B4E8A3115.2	*TaWRKY78*	III										
Traes_5BL_0A3D332A8.1	*TaWRKY84*	III										
Traes_5BL_175E7FC38.1	*TaWRKY85*	III				*						
Traes_5DL_C93641E43.1	*TaWRKY92*	III										
Traes_5DL_5C93510D5.1	*TaWRKY95*	III										
Traes_6AS_68775100B.1	*TaWRKY101*	III										
Traes_7DS_01F74C6F3.1	*TaWRKY110*	III							*			
Traes_7DL_A9EF00572.1	*TaWRKY112*	III						*				
Traes_2AS_1AFFE8DA6.1	*TaWRKY121*	III								*		
Traes_2DL_362A1F535.1	*TaWRKY122*	III										
Traes_3B_8B0D448D8.1	*TaWRKY132*	III										
Traes_3DL_2551BF2C1.1	*TaWRKY136*	III										
Traes_4AS_0DA136E0E.1	*TaWRKY142*	III										
Traes_5DL_09F1F8F79.1	*TaWRKY160*	III				*						
Traes_7BL_53AA25AA1.1	*TaWRKY169*	III										

Transcript ID	Proposed name	Group	4cl-CMA2b	ATCC-motif	GATT-motif	chs-CMA2c	Pc-CMA2c	chs-CMA1a	chs-CMA2b	LS7	CGT-motif	as-2-box
			light responsiveness	light responsiveness	light responsiveness	light responsiveness	light responsiveness	part of a light response element	light responsiveness	light responsiveness	light responsiveness	shoot-specific expression and light responsiveness
Traes_1AS_F3EAEC435.1	*TaWRKY1*	I										
Traes_1BS_EF67E5A24.1	*TaWRKY8*	I										
Traes_2AS_C407071E4.2	*TaWRKY23*	I										*
Traes_2AS_0186B9E4F.2	*TaWRKY25*	I										
Traes_2AL_409AB7647.1	*TaWRKY26*	I								*		
Traes_2BL_2BA3A755A.1	*TaWRKY30*	I										
Traes_2BS_380EC4D1E.1	*TaWRKY32*	I										
Traes_2BS_D435A8999.1	*TaWRKY33*	I		*								*
Traes_2DS_97E3E7CFC.1	*TaWRKY39*	I										
Traes_2DS_F6FBC974C.2	*TaWRKY41*	I										
Traes_4BL_EFEC50B26.2	*TaWRKY69*	I										
Traes_4DS_DC3C9DC42.2	*TaWRKY75*	I										
Traes_5AL_ED3ADED51.3	*TaWRKY77*	I										*
Traes_5BS_C46781248.1	*TaWRKY82*	I										*
Traes_5BL_A522C62D1.1	*TaWRKY83*	I										
Traes_5BL_17A712C94.1	*TaWRKY88*	I					*					
Traes_5DS_5DEA5C9E3.1	*TaWRKY94*	I										
Traes_5DL_21F7C6BF7.2	*TaWRKY96*	I										
Traes_5DL_46E3AC8D6.1	*TaWRKY98*	I										
Traes_5DL_4BA2CC560.2	*TaWRKY99*	I										
Traes_7BL_A46F1A830.2	*TaWRKY109*	I										
Traes_7DS_24C563960.1	*TaWRKY111*	I										
Traes_1DS_A6733B734.1	*TaWRKY120*	I										
Traes_3B_990298FF5.1	*TaWRKY133*	I										
Traes_4AL_2EEECCC4B.1	*TaWRKY141*	I										
Traes_5AL_E644A6A0B.1	*TaWRKY145*	I							*			
Traes_5BL_D3C383CF5.1	*TaWRKY152*	I										*
Traes_7AL_48C81DE03.1	*TaWRKY168*	I										
Traes_7AL_48C81DE031.1	*TaWRKY171*	I										
Traes_5BL_8BEF7F9CD.1	*TaWRKY86*	IIa										
Traes_5BL_90757F0CC.1	*TaWRKY87*	IIa										
Traes_7DL_5968FA56C.1	*TaWRKY113*	IIa										
Traes_7DL_B09854286.1	*TaWRKY114*	IIa										
Traes_7DL_F849918EA.2	*TaWRKY115*	IIa										
Traes_6AS_DA75BB1FD.1	*TaWRKY161*	IIa										
TRAES3BF003800010CFD_t1	*TaWRKY47*	IIb										
Traes_4AL_3E11167D9.1	*TaWRKY68*	IIb										
Traes_6AL_0C0899C15.1	*TaWRKY104*	IIb										
Traes_7AS_C9DF68E53.2	*TaWRKY108*	IIb										
Traes_6DL_AB95B0CE0.1	*TaWRKY164*	IIb										
Traes_1AS_1432A2F79.1	*TaWRKY2*	IIc										
Traes_1AL_0404BC790.1	*TaWRKY6*	IIc										
Traes_1AL_9ADA7A031.1	*TaWRKY7*	IIc										
Traes_1BL_1D865A8CC.1	*TaWRKY11*	IIc										
Traes_1BL_9AFA4B870.1	*TaWRKY12*	IIc										
Traes_1DL_D550418641.2	*TaWRKY15*	IIc										
Traes_1DL_5BAB0B6BC.1	*TaWRKY16*	IIc										*
Traes_1DL_D1EC7DEA6.1	*TaWRKY17*	IIc		*								
Traes_1DL_46428511F.1	*TaWRKY18*	IIc							*			
Traes_2AS_D0C21ADB5.1	*TaWRKY19*	IIc										
Traes_2AL_1B43EA59E.1	*TaWRKY20*	IIc										
Traes_2AL_B1270662B.1	*TaWRKY21*	IIc										
Traes_2BS_F3097F116.1	*TaWRKY28*	IIc										
Traes_2BL_A69F6C5DF.1	*TaWRKY31*	IIc										
Traes_2BL_A5BFA97B9.1	*TaWRKY34*	IIc										
Traes_2DS_0F2500A60.1	*TaWRKY36*	IIc										
Traes_2DL_4F9F8F1F0.1	*TaWRKY38*	IIc										
Traes_3AL_67ECA2932.1	*TaWRKY42*	IIc									*	
Traes_3AL_1B73D2C12.1	*TaWRKY43*	IIc										
TRAES3BF066700160CFD_t1	*TaWRKY45*	IIc										
TRAES3BF021300010CFD_t1	*TaWRKY48*	IIc			*							
TRAES3BF267200010CFD_t1	*TaWRKY49*	IIc										
TRAES3BF021100090CFD_t1	*TaWRKY50*	IIc			*							
TRAES3BF058500060CFD_t1	*TaWRKY52*	IIc										
TRAES3BF111700140CFD_t1	*TaWRKY53*	IIc										
TRAES3BF090100100CFD_t1	*TaWRKY61*	IIc										
TRAES3BF045500040CFD_t1	*TaWRKY62*	IIc										
Traes_3DL_DF0D3F3FE.1	*TaWRKY63*	IIc										*
Traes_5AL_E566BD64E.1	*TaWRKY79*	IIc					*					
Traes_5BL_C1D6B6B74.2	*TaWRKY90*	IIc										
Traes_5DL_7E2053226.2	*TaWRKY97*	IIc										*

Traes_6AL_BA4636569.1	*TaWRKY102*	IIc							
Traes_6BL_DD840863A.1	*TaWRKY105*	IIc							*
Traes_6DL_D29E210A1.1	*TaWRKY106*	IIc							
Traes_1AL_F64E07A92.1	*TaWRKY116*	IIc							
Traes_1BL_B15990028.1	*TaWRKY118*	IIc							
Traes_3AL_4769A72F1.1	*TaWRKY125*	IIc							
Traes_3AL_AB2BAE660.1	*TaWRKY129*	IIc							
Traes_3AL_140B829CB.2	*TaWRKY130*	IIc			*				
Traes_4AL_234E1CDF6.1	*TaWRKY140*	IIc							
Traes_4DS_CFC487CE5.2	*TaWRKY143*	IIc							*
Traes_6DS_BF71C1557.2	*TaWRKY166*	IIc							
Traes_2AL_434E9F101.1	*TaWRKY22*	IId							
Traes_2AL_15A7BB684.1	*TaWRKY27*	IId				*			*
Traes_2BL_6B75B32E3.1	*TaWRKY35*	IId				*			*
Traes_2DL_F600B5FDF.1	*TaWRKY37*	IId							
Traes_4AL_98B1C762B.1	*TaWRKY67*	IId						*	
Traes_4BS_CE839571B.2	*TaWRKY71*	IId							
Traes_4DS_3BE557D5C.2	*TaWRKY73*	IId							
Traes_5AL_6F7D1D441.1	*TaWRKY80*	IId							
Traes_5BS_E0345D5DF.2	*TaWRKY81*	IId	*						
Traes_5BL_8688F70C9.1	*TaWRKY89*	IId							
Traes_5BL_E294922A9.2	*TaWRKY91*	IId							
Traes_5DS_D83DEA9B0.1	*TaWRKY93*	IId	*						
Traes_5BL_AEF9FE805.2	*TaWRKY154*	IId							
Traes_1AL_4E924201A.1	*TaWRKY3*	IIe							
TRAES3BF180700010CFD_t1	*TaWRKY51*	IIe							
TRAES3BF073300120CFD_t1	*TaWRKY54*	IIe				*			*
TRAES3BF029000080CFD_t1	*TaWRKY55*	IIe							
Traes_5DL_E4A6D1889.2	*TaWRKY100*	IIe							
Traes_6AL_A5FB7CFA5.1	*TaWRKY103*	IIe							
Traes_6DL_D4F2CDDDC.1	*TaWRKY107*	IIe							
Traes_1BL_794E99FF5.1	*TaWRKY119*	IIe							
Traes_5BL_B9DD3E76F.1	*TaWRKY155*	IIe							
Traes_1AL_309623B48.1	*TaWRKY4*	III							
Traes_1AL_B24F28600.1	*TaWRKY5*	III							
Traes_1BL_46340D685.1	*TaWRKY9*	III					*		
Traes_1BL_B4AFDB663.1	*TaWRKY10*	III							
Traes_1BL_73811B853.1	*TaWRKY13*	III							*
Traes_1DL_DFE1721E0.1	*TaWRKY14*	III							
Traes_2AS_6269D889E.1	*TaWRKY24*	III							*
Traes_2BS_B65714572.1	*TaWRKY29*	III							
Traes_2DS_AD8820C42.1	*TaWRKY40*	III							
Traes_3AL_2297D6E18.1	*TaWRKY44*	III							
TRAES3BF051200110CFD_t1	*TaWRKY46*	III	*						
TRAES3BF081400030CFD_t1	*TaWRKY56*	III							*
TRAES3BF001300030CFD_t1	*TaWRKY57*	III							
TRAES3BF005100010CFD_t1	*TaWRKY58*	III							
TRAES3BF005100020CFD_t1	*TaWRKY59*	III							
TRAES3BF005100030CFD_t1	*TaWRKY60*	III							
Traes_3DL_48F7A19D2.1	*TaWRKY64*	III							
Traes_4AS_70DF607CC.1	*TaWRKY65*	III							
Traes_4AL_9E0D1CFA6.1	*TaWRKY66*	III							
Traes_4BS_A6D9EB0E5.1	*TaWRKY70*	III							
Traes_4BL_A8C6FBEB6.1	*TaWRKY72*	III							
Traes_4DS_FE38A59D0.1	*TaWRKY74*	III						*	
Traes_4DL_3140A8240.1	*TaWRKY76*	III							
Traes_5AL_B4E8A3115.2	*TaWRKY78*	III							*
Traes_5BL_0A3D332A8.1	*TaWRKY84*	III							
Traes_5BL_175E7FC38.1	*TaWRKY85*	III							
Traes_5DL_C93641E43.1	*TaWRKY92*	III							
Traes_5DL_5C93510D5.1	*TaWRKY95*	III							
Traes_6AS_68775100B.1	*TaWRKY101*	III							
Traes_7DS_01F74C6F3.1	*TaWRKY110*	III		*					*
Traes_7DL_A9EF00572.1	*TaWRKY112*	III							
Traes_2AS_1AFFE8DA6.1	*TaWRKY121*	III							
Traes_2DL_362A1F535.1	*TaWRKY122*	III							
Traes_3B_8B0D448D8.1	*TaWRKY132*	III							
Traes_3DL_2551BF2C1.1	*TaWRKY136*	III							
Traes_4AS_0DA136E0E.1	*TaWRKY142*	III							
Traes_5DL_09F1F8F79.1	*TaWRKY160*	III							
Traes_7BL_53AA25AA1.1	*TaWRKY169*	III							
