# Supplementary material for: Genome-wide analysis of WRKY transcription factors in wheat (Triticum aestivum L.) and differential expression under water deficit condition
Source: PeerJ. 2017 May 4;5:e3232. doi: 10.7717/peerj.3232 (PMC5420200; doi:10.7717/peerj.3232)
Supplement: Table S6 [file peerj-05-3232-s008.pdf]

**Supplemental Table S6. Drought responsive *AtWRKYs* in in *Arabidopsis* and their putative orthologous *TaWRKYs* in wheat.**

| Arabidopsis | | Wheat | | |
|---|---|---|---|---|
| **Transcript ID** | **Number** | **Transcript ID** | **Proposed name** | **Number of amino acids (aa)** |
| AT2G38470.1 | AtWRKY33 | Traes_1AS_F3EAEC435.1 | TaWRKY1 | 440 |
| | | Traes_1BS_EF67E5A24.1 | TaWRKY8 | 458 |
| | | Traes_1DS_A6733B734.1 | TaWRKY120 | 464 |
| | | Traes_3B_990298FF5.1 | TaWRKY133 | 559 |
| AT1G69310.1 | AtWRKY57 | Traes_2AL_1B43EA59E.1 | TaWRKY20 | 228 |
| | | Traes_2BL_A69F6C5DF.1 | TaWRKY31 | 285 |
| | | Traes_5BL_C1D6B6B74.2 | TaWRKY90 | 310 |
| | | Traes_5DL_7E2053226.2 | TaWRKY97 | 208 |
| | | Traes_2DL_04535D371.1 | TaWRKY123 | 285 |
| | | Traes_5AL_06A6F9328.2 | TaWRKY149 | 193 |
| AT1G66600.1 | AtWRKY63 | Traes_7DL_A9EF00572.1 | TaWRKY112 | 226 |
| | | Traes_2DL_362A1F535.1 | TaWRKY122 | 70 |
| | | Traes_4AS_0DA136E0E.1 | TaWRKY142 | 135 |
| | | Traes_7BL_53AA25AA1.1 | TaWRKY169 | 55 |
